# Gap Junction Proteins in the Blood-Brain Barrier Control Nutrient-Dependent Reactivation of *Drosophila* Neural Stem Cells

**DOI:** 10.1016/j.devcel.2014.05.021

**Published:** 2014-08-11

**Authors:** Pauline Spéder, Andrea H. Brand

**Affiliations:** 1 The Gurdon Institute and Department of Physiology, Development and Neuroscience, University of Cambridge, Tennis Court Road, Cambridge CB2 1QN, UK

## Abstract

Neural stem cells in the adult brain exist primarily in a quiescent state but are reactivated in response to changing physiological conditions. How do stem cells sense and respond to metabolic changes? In the *Drosophila* CNS, quiescent neural stem cells are reactivated synchronously in response to a nutritional stimulus. Feeding triggers insulin production by blood-brain barrier glial cells, activating the insulin/insulin-like growth factor pathway in underlying neural stem cells and stimulating their growth and proliferation. Here we show that gap junctions in the blood-brain barrier glia mediate the influence of metabolic changes on stem cell behavior, enabling glia to respond to nutritional signals and reactivate quiescent stem cells. We propose that gap junctions in the blood-brain barrier are required to translate metabolic signals into synchronized calcium pulses and insulin secretion.

## Highlights

• Blood-brain barrier gap junctions are required for neural stem cell reactivation
• Gap junctions control both insulin transcription and secretion
• Calcium oscillations in the blood-brain barrier depend on gap junctions and nutrition
• Blood-brain barrier membrane polarization links calcium to insulin secretion

## Graphical Abstract

**Figure:**
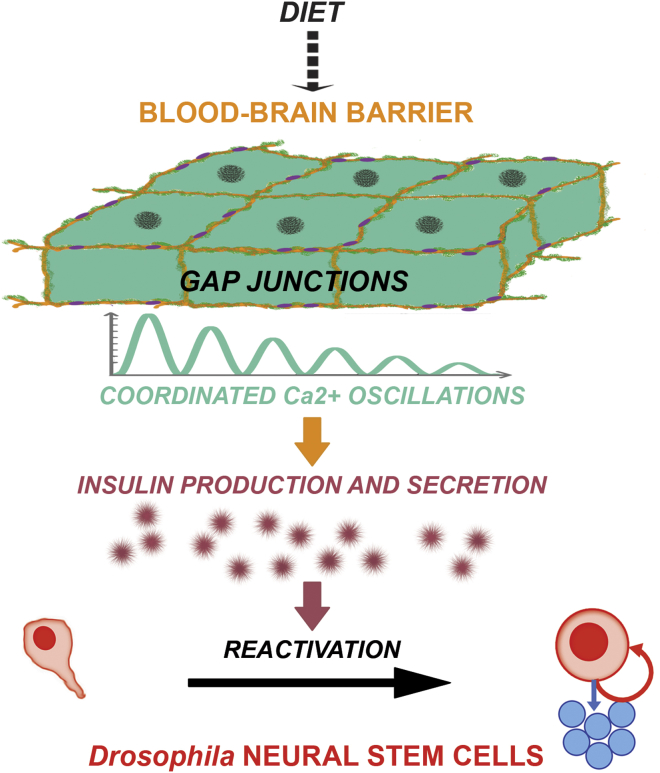

## Teaser

Spéder and Brand provide evidence that gap junctions in the blood-brain barrier act as a relay that converts nutritional signals into local insulin secretion via synchronized calcium oscillations for neural stem cell reactivation. Calcium oscillations are coordinated via gap junction proteins, which control the production and secretion of insulin-like peptides.

## Introduction

Changes in environmental conditions can have a significant impact on the development and function of the brain. Neural stem cells (NSCs) integrate both local and systemic signals to modulate their rate and extent of proliferation to meet the needs of the organism (Kokovay et al., 2008). Most NSCs in the vertebrate adult brain exist in a mitotically dormant state. These quiescent NSCs are reactivated in response to a variety of metabolic stimuli (Rafalski and Brunet, 2011). Understanding how systemic and metabolic signals are sensed by the brain and converted into specific neural stem cell behaviors is essential to deciphering how the brain adapts to a changing environment.

**Figure 1:**
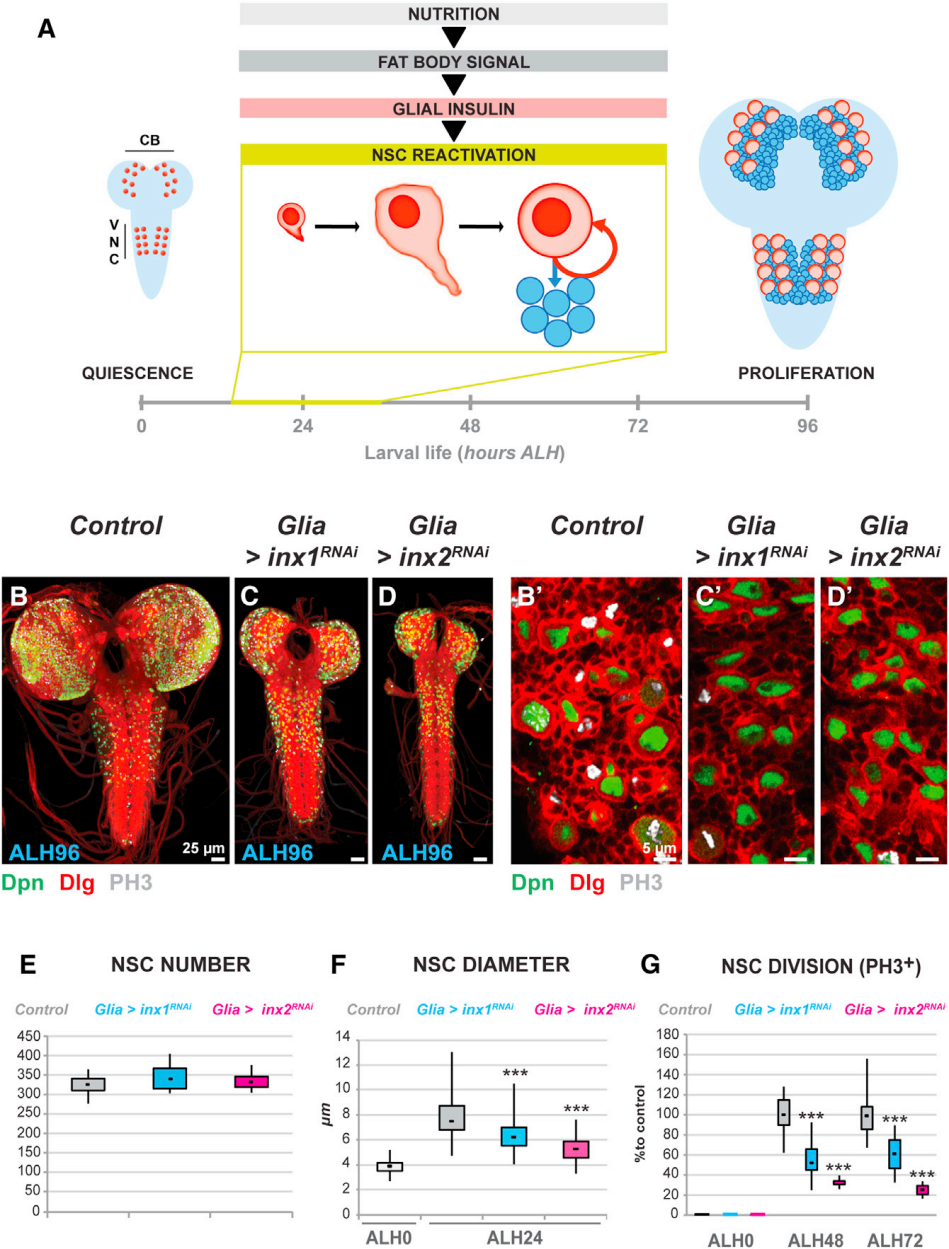
Glial Gap Junctions Are Required for Nutrient-Dependent Reactivation of NSCs (A) *Drosophila* quiescent NSCs are reactivated within a 24 hr time window in response to nutrition. CB, central brain; VNC, ventral nerve cord; ALH, hours after larval hatching. (B–D) Total brain size, NSC diameter and NSC proliferation are highly reduced after *inx1* or *inx2* RNAi in glia. (B′–D′) Higher magnification ventral views of the VNCs in (B–D). (E–G) Quantification of NSC (E) number, (F) diameter, and (G) proliferation in *inx1* or *inx2* knockdown in glia. (E) ^∗∗∗^p < 0.05. Two-sided Student’s t test. Average and SD were calculated from two biological replicates. Control n = 8 VNCs; *Glia > inx1*^*RNAi*^ n = 11 VNCs; *Glia > inx2*^*RNAi*^ n = 7 VNCs. *Glia > inx1*^*RNAi*^ p = 0.14. *Glia > inx2*^*RNAi*^ p = 0.53. (F) ^∗∗∗^p < 0.05. Two-sided Student’s t test. Average and SD were calculated from two biological replicates. Control ALH0 n = 224 NSCs (8 VNC); Control ALH24 n = 241 NSCs (15 VNCs); *Glia > inx1*^*RNAi*^ ALH24 n = 257 NSCs (11 VNCs); *Glia > inx2*^*RNAi*^ ALH24 n = 264 NSCs (11 VNCs). For ALH24: *Glia > inx1*^*RNAi*^ p = 1.23 × 10^−28^; *Glia > inx2*^*RNAi*^ p = 2.47 × 10^−78^. (G) ^∗∗∗^p < 0.05. Two-sided Student’s t test. Average and SD were calculated from two biological replicates. AH48. Control n = 17 VNCs; *Glia > inx1*^*RNAi*^ n = 18 VNCs; *Glia > inx2*^*RNAi*^ n = 8 VNCs. *Glia > inx1*^*RNAi*^ p = 3.52 × 10^−7^. *Glia > inx2*^*RNAi*^ p = 2.00 × 10^−8^. AH72. Control n = 14 VNCs; *Glia > inx1*^*RNAi*^ n = 13 VNCs; *Glia > inx2*^*RNAi*^ n = 4 VNCs. *Glia > inx1*^*RNAi*^ p = 1.11 × 10^−4^. *Glia > inx2*^*RNAi*^ p = 1.40 × 10^−5^. All images are anterior up, dorsal view unless stated otherwise. NSC nuclei, green (Deadpan, Dpn); Cell cortices, red (Discs Large, Dlg); PH3, gray (phospho-histone H3). See also Figure S1 and Table S1.

In *Drosophila*, NSCs enter quiescence at the end of embryogenesis and are reactivated during early larval life in response to feeding (Britton and Edgar, 1998, Truman and Bate, 1988; Figure 1A). Amino acid availability is sensed by the fat body, the functional equivalent of the mammalian liver and adipose tissue (Andersen et al., 2013, Colombani et al., 2003). The fat body sends an as-yet-unidentified signal, or signals, to the brain to induce the production and secretion of insulin-like peptides (dIlps) by blood-brain barrier (BBB) glial cells. dIlps act locally to trigger the insulin/insulin-like growth factor receptor pathway in underlying NSCs (Chell and Brand, 2010, Sousa-Nunes et al., 2011). Consequently, the NSCs enlarge and re-enter the cell cycle.

NSC reactivation occurs synchronously in all neurogenic zones of the CNS, suggesting that BBB glial cells and/or NSCs are linked by an intercellular signaling mechanism. Gap junctions are intercellular channels formed by the juxtaposition of connexin hexamers (Segretain and Falk, 2004). They enable the propagation and amplification of signals within or between cell populations. Gap junctions are found throughout the mammalian brain and are important regulators of stem cell behavior, controlling self-renewal, survival, and aging (Kar et al., 2012, Wong et al., 2008). Here we show that gap junction proteins play a key role in the nutrient-dependent reactivation of dormant neural stem cells in the *Drosophila* brain. Interestingly, gap junction proteins are required in the BBB glia, but not in neural stem cells, for reactivation. We show that gap junction proteins coordinate nutrient-dependent calcium oscillations within the BBB and are required for the production and secretion of insulin-like peptides. Gap junction proteins thus enable the synchronous reactivation of quiescent stem cells throughout the CNS.

## Results

### Inx1 and Inx2 Are Required for Normal Brain Development

To assess whether gap junctions play a role in NSC reactivation, we systematically targeted each of the eight members of the innexin (Inx) family (Bauer et al., 2005), the *Drosophila* functional equivalents of connexins and pannexins (Phelan, 2005; Figure S1A available online), by RNAi in either NSCs or glia (Table S1). Interestingly, we observed no detectable phenotype when innexins were knocked down in NSCs. However, knockdown of *innexin 1* (*inx1*) or *innexin 2* (*inx2*) in glia gave a striking phenotype in which brain size is dramatically reduced (Figures 1B–1D′) without affecting overall body size (data not shown). This suggests that the *inx* phenotype is not the result of a systemic growth defect but that *inx1* and *inx2* have a specific role in the brain. We checked the specificy of *inx1*^*RNAi*^ and *inx2*^*RNAi*^ using in silico methods (Naito et al., 2005), which predict no off-targets. Our data are consistent with the recent results of Holcroft et al., 2013, who showed that targeted RNAi against *inx1* (*ogre*) or *inx2* in glia disrupts development of the larval nervous system and leads to adult behavioral phenotypes. We demonstrate that innexins are not required to link NSCs either to each other or to glial cells. Instead, Inx1 and Inx2 are required within the glial population alone for brain development.

### Gap Junction Proteins Are Required in Glia for NSC Exit from Quiescence

To understand how glial gap junctions regulate growth in the CNS, we first examined NSC behavior after *inx1* or *inx2* knockdown at different time points during the process of NSC reactivation. Knockdown of *inx1* or *inx2* in glia did not affect the number of NSCs in the ventral nerve cord (VNC; Figure 1E), demonstrating that the phenotype is not due to the loss of NSCs prior to NSC reactivation (0 hr after larval hatching, ALH0). Next we assessed cell diameter because one of the earliest events in NSC exit from quiescence is cell enlargement (Chell and Brand, 2010, Sousa-Nunes et al., 2011). We found that NSC diameter is markedly reduced (ALH24) after *inx1* or *inx2* knockdown in glia (Figure 1F). Finally, we assessed NSC proliferation after *inx* knockdown. We assayed the mitotic marker phosphohistone H3 (PH3) before NSC reactivation (ALH0), just after reactivation (ALH48) and at a time when wild-type NSCs are cycling actively (ALH72). Knockdown of either *inx1* or *inx2* in glial cells resulted in a severe reduction in the number of dividing NSCs at all times (Figure 1G). We found that NSC enlargement and entry into mitosis were also dramatically impaired in *inx1* and *inx2* mutants (*inx1*^*ogrejNL3*^ and *inx2*^*G0036*^, respectively, see Figures S1B and S1C), and that reactivation could be rescued in *inx2* mutants by glial expression of *inx2* (Figures S1D–S1E′). We conclude that *inx1* and *inx2* are required in the glia for NSC exit from quiescence.

### Inx1 and Inx2 Form Heteromeric Complexes

**Figure 2:**
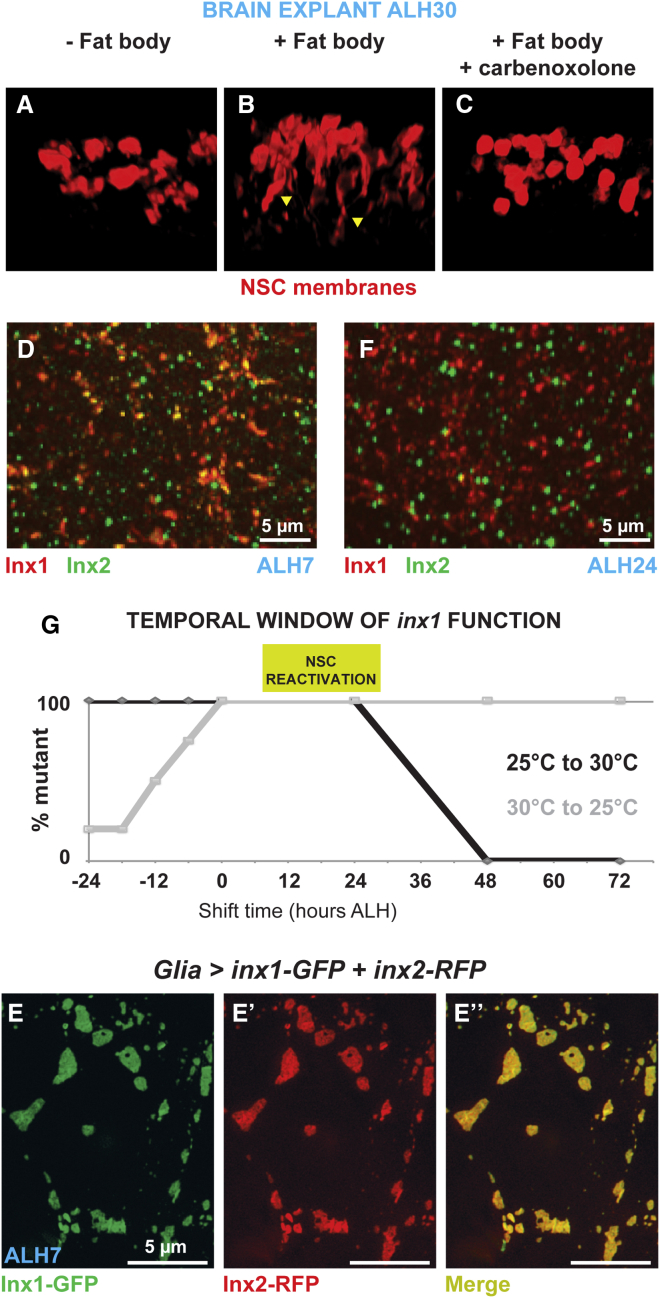
Inx1 and Inx2 Form Heteromeric and Temporally Regulated Channels in Glia (A–C) Brain explant culture experiment. Brains from ALH0 larvae were cultured either on standard medium (A, − fat body), supplemented with fat body extract (B, + fat body) or supplemented with fat body extract and carbenoxolone (C, + fat body + carbenoxolone). Each image represents a three-dimensional reconstruction of a part of the VNC. Arrowheads indicate processes that NSCs extend during reactivation. See text and Supplemental Experimental Procedures for details of the protocol. NSC membrane, red (*grh-GAL4, UAS-myrRFP*). (D) Confocal images showing colocalization of Inx1 (red) and Inx2 (green) in the VNC before NSCs enlarge. Extended projection. (E–E′′) Inx1-GFP (green) and Inx2-RFP (red), colocalize in plaques in glia (super resolution image). (F) After NSCs exit quiescence Inx1 (red) and Inx2 (green) no longer colocalize. (G) Temporal requirement of *inx1* activity. Temperature shifts of *tub-Gal80*^*ts*^*, UAS-inx1*^*RNAi*^*; repo-GAL4* flies, from the permissive (25°C) to the restrictive temperature (30°C; black) and vice versa (gray) identify the temperature sensitive period as between 0 and 24 hr ALH. The mutant phenotype was scored based on a reduction in brain volume. For each time point, 12 brains were analyzed. ALH, after larval hatching (at 29°C). See also Figure S2 and Table S2.

Gap junction proteins (connexins, pannexins, or innexins) are classically involved in forming intercellular channels or hemi-channels, which enable exchange between the cytoplasm and the extracellular medium. Evidence also exists for channel-independent roles, such as cell adhesion and direct gene regulation (reviewed in Dbouk et al., 2009, Elias and Kriegstein, 2008). To test if channel function is important for NSC reactivation, we treated brains in culture with carbenoxolone, a classic blocker of gap junction channels and hemi-channels (Giaume and Theis, 2010; see Supplemental Experimental Procedures). Carbenoxolone completely blocked NSC reactivation (Figures 2A–2C), implying a channel role for Inx1 and/or Inx2, the only innexins required for NSC reactivation. We also found that protein fusions that interfere with the folding of the innexin N-terminal domain (GFP-Inx1 and RFP-Inx2), which is essential for channel formation (Nakagawa et al., 2010), act as dominant-negative mutants (Figures S2A–S2C; data not shown for Inx2). This suggests that the function of Inx1 and Inx2 in glia is channel-based.

Inx1 and Inx2 could be part of the same channel or form two distinct channels, performing different functions that are both required for NSC reactivation. Gap junction channels are formed by the apposition of connexons (innexons in *Drosophila*) on adjacent cells. Connexons can be homomeric, formed from six molecules of a single subtype of connexin, or heteromeric, formed from different subtypes. In the larval VNC prior to NSC reactivation (ALH7), Inx1 and Inx2 were strongly expressed in glia and colocalized in plaques typical of gap junctions (Segretain and Falk, 2004; Figure 2D). Super-resolution microscopy (Dobbie et al., 2011) further demonstrated the tight association of Inx1 and Inx2, using tagged fusion proteins (Figures 2E–2E′′). This close association is present from hatching and is not lost under starvation conditions (Figures S2D and S2E), demonstrating that formation of the complex is not driven by nutrition.

We found that most Inx1 staining was lost after knockdown of *inx2* in glia, and vice versa. This suggests that Inx1 and Inx2 localization is interdependent (Figures S2F–S2H) and that Inx1 and Inx2 form heteromeric innexons (Segretain and Falk, 2004) rather than independent gap junction channels (Lehmann et al., 2006). Inx1 and Inx2 have been shown to form functional heteromeric channels in paired *Xenopus* oocytes (Holcroft et al., 2013). We conclude, therefore, that Inx1 and Inx2 form heteromeric channels or hemi-channels in the glia.

### Inx1 and Inx2 Channels Are Developmentally Regulated

Interestingly, we observed a change in Inx1/Inx2 colocalization over time. By ALH24, when reactivation has taken place, Inx1 and Inx2 are still expressed but they no longer colocalize (Figure 2F), suggesting that formation of Inx1/Inx2 channels is temporally regulated. Consistent with this observation, we discovered that the temporal requirement for *inx1* and *inx2* function in NSC reactivation is between ALH0 and ALH24 (Figure 2G, Table S2, and data not shown). Therefore, the formation and maintenance of Inx1 and Inx2 heteromeric channels are developmentally regulated and coincide with the time when the innexins are required for NSC reactivation.

### Inx1/Inx2 Gap Junctions Are Required in the BBB Glia for NSC Reactivation

**Figure 3:**
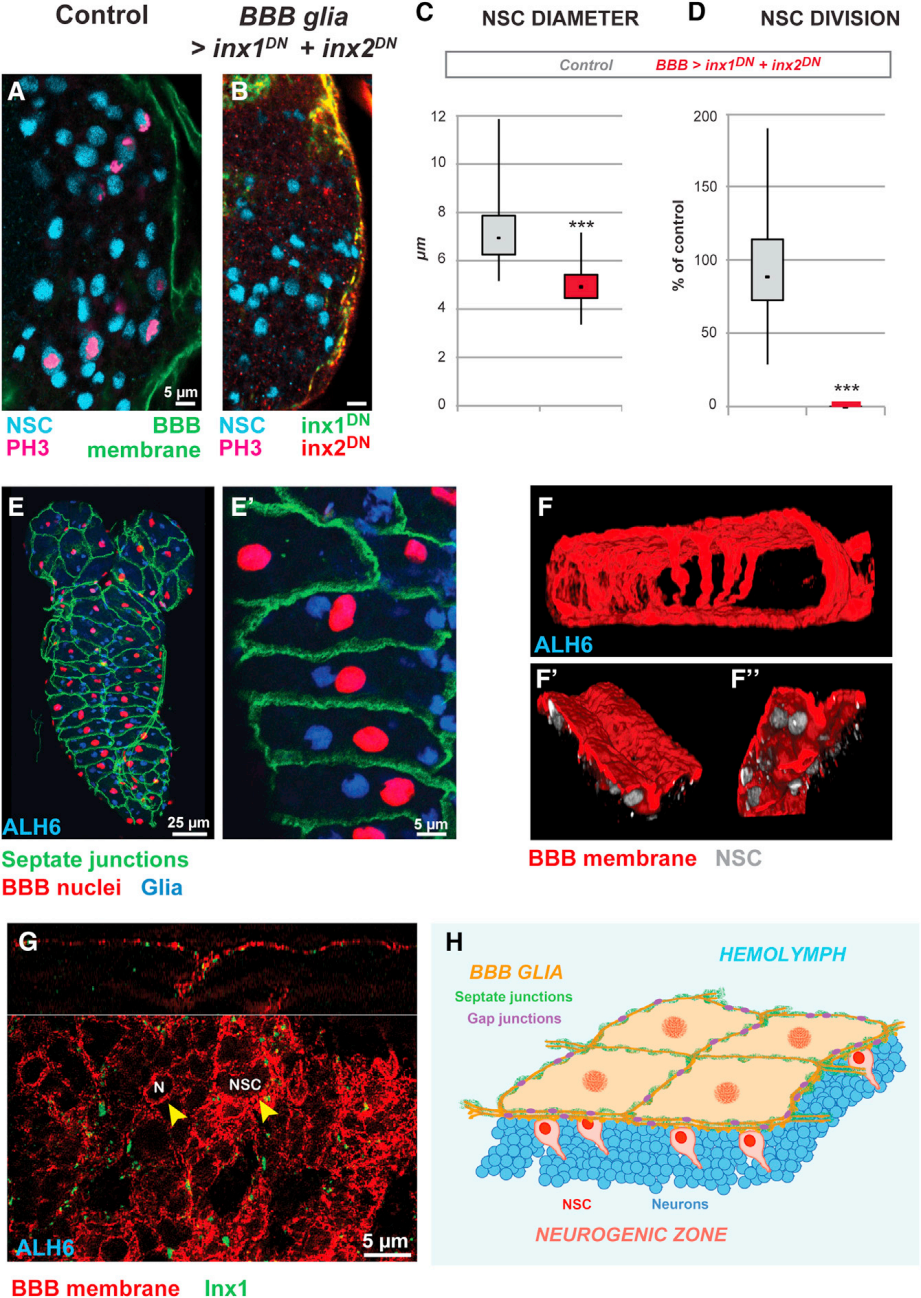
Inx1 and Inx2 Are Required in the BBB Glia for NSC Reactivation (A–D) Blocking *inx1* and *inx2* function in BBB glia phenocopies pan-glial knockdown. Confocal images of VNC NSCs in (A) control brain (*mdr65-GAL4, UAS-mCD8-GFP*) and (B) brain where dominant-negative forms of Inx1 and Inx2 have been driven in the subperineurial glia only (*mdr65-GAL4* driving *inx1*^*DN*^ and *inx2*^*DN*^). NSC, cyan (Dpn); Phospho-histone H3, magenta; BBB membrane, green (GFP) or inx1^DN^ (GFP-Inx1), green and inx2^DN^ (RFP-Inx2), red. Quantification of NSC (C) diameter and (D) proliferation. (C) ^∗∗∗^p < 0.05. Two-sided Student’s t test. Average and standard deviation were calculated from two biological replicates. Control n = 148 NSCs (11 VNC); *BBB Glia > inx1*^*DN*^*+ inx2*^*DN*^ n = 143 NSCs (10 VNCs). p = 4.19 × 10^−53^. (D) ^∗∗∗^p < 0.05. Two-sided Student’s t test. Average and standard deviation were calculated from two biological replicates. Control n = 11 VNCs; *BBB Glia > inx1*^*DN*^*+ inx2*^*DN*^ n = 10 VNCs. p = 4.44 × 10^−4^. (E and E′) Organization of early BBB glial cells. (E′) Close up of (E). Septate junctions, green (Lachesin-GFP); BBB nuclei, red (*moody-GAL4*, *UAS-Histone-RFP*); glial nuclei, blue (Repo). Anterior up, ventral view. (F–F′′) Three-dimensional reconstruction of a part of the BBB membrane, red (*moody-GAL4*, *UAS-mCD8-RFP*). Ventral up, dorsal down. (F′ and F′′) Apical and basal views of ventral BBB membrane, respectively. NSC, gray (Dpn). (G) Super resolution image of BBB glial membrane. Inx1, green; membrane, red (*Moody-GAL4, UAS-mCD8-RFP*). Top: orthogonal section; bottom panel, single focal plane of the ventral BBB glia. The “holes” (see arrowheads) in the BBB membrane are created by the NSCs or neurons (N) upon which the BBB glia rest like a sheet, resulting in small invaginations. (H) Schematics of the BBB. See also Figure S3 and Table S3.

Inx1/Inx2 channels are required in glia to transmit nutritional stimuli to quiescent NSCs, They are likely to be found, therefore, in cells situated between the NSCs and the exterior of the brain. To determine in which glial cells Inx1/Inx2 are required, we knocked down Inx1/Inx2 in different glial populations using subtype-restricted GAL4 drivers to drive RNAi or express dominant-negative constructs (Table S3). We found that *inx* function is necessary within the subperineurial glia because knockdown in this glial subtype alone phenocopies knockdown in the entire glial population (Table S3 and Figures S3A and S3B), preventing NSC reactivation (Figures 3A–3D).

The subperineurial glia and the perineurial glia constitute the *Drosophila* BBB (Stork et al., 2008). In vertebrates, the BBB consists of a single layer of vascular endothelium closely associated with astrocytic glia (Daneman, 2012). The BBB shields the brain from the external environment owing to tight junctions between endothelial cells. It acts as a selective sieve to reject potentially neurotoxic factors but allow the passage of nutrients, ions, or other signals to maintain brain homeostasis (Daneman, 2012).

The *Drosophila* BBB exhibits similar neuroprotective strategies to its vertebrate counterpart, including a layer that limits the diffusion of neurotoxic factors, and an array of conserved transporters that regulates BBB permeability (Daneman and Barres, 2005, DeSalvo et al., 2011, Mayer et al., 2009). The subperineurial glia are large, flat polyploid (Unhavaithaya and Orr-Weaver, 2012) cells (Figures 3E–3E′) that envelop the brain (Figure 3F) and are closely apposed to the NSCs (Figures 3F′ and 3F′′). They isolate the brain from the hemolymph (the *Drosophila* equivalent of blood) by virtue of lateral septate junctions (Figures 3E and 3E′; Schwabe et al., 2005, Stork et al., 2008).

Knockdown of *inx* did not disrupt the septate junctions (Figures S3C and S3D), and we were not able to see a change in Dextran dye penetration (data not shown; Pinsonneault et al., 2011, Schwabe et al., 2005). Although weak permeability defects are difficult to detect at this stage and cannot be excluded, they do not prevent NSC reactivation (*moody*^Δ*17*^ mutant (Bainton et al., 2005), data not shown). These data suggest that the *inx* mutant phenotype is not due to an impaired, leaky BBB. Using super-resolution microscopy, we detected Inx1 (and Inx2, data not shown) along the BBB membranes and the septate junctions lining the lateral cell membranes (Figures 3G and S3E). We conclude that Inx1/Inx2 channels are required autonomously in the BBB glial cells for NSC reactivation (Figure 3H).

### Inx1/Inx2 Gap Junctions Are Required for Insulin Signaling

NSC reactivation requires the expression and secretion of insulin-like peptides, dIlps, by BBB glial cells (Chell and Brand, 2010). Of the eight identified insulin-like peptides in *Drosophila*, *dIlp6* transcription was shown to increase dramatically in the CNS upon feeding. Furthermore, when larvae were starved, forced expression of *dIlp6* in the glia was able to rescue NSC reactivation (Chell and Brand, 2010). dIlp6 binds to the insulin receptor (InR) on NSCs, activating the PI3K/Akt pathway and inducing exit from quiescence (Chell and Brand, 2010).

**Figure 4:**
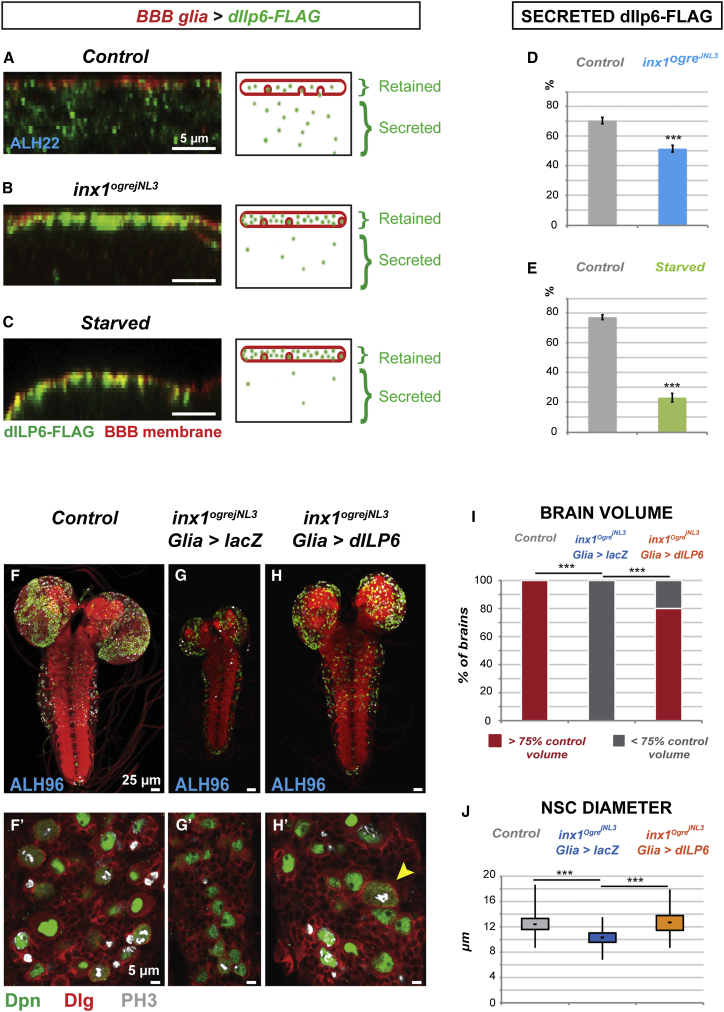
Gap Junctions Control NSC Reactivation through the Insulin Pathway (A–D) dIlp6 secretion depends on gap junction and nutrition. dIlp6 secretion was assayed by expressing a tagged version in the BBB glia only (*moody-GAL4, UAS-mCD8-RFP, UAS-dIlp6-FLAG,*) and assessing what is found out of the BBB glia. Cross-section of one BBB glial cell and its schematic for (A) control, (B) *inx1*^*ogrejNL3*^ mutant, and (C) starved. dIlp6-FLAG, green (FLAG); BBB membrane, red (RFP). (D and E) Secreted dIlp6-FLAG (%) was measured as the ratio between secreted dIlp6-FLAG and total dIlp6-FLAG. See Experimental Procedures for details. ^∗∗∗^p < 0.05. Two-sided Student’s test. (D) Control n = 38 VNCs; *inx1*^*ogrejNL3*^ n = 35 VNCs. p = 7.21 × 10^−8^. (E) Fed n = 19 VNCs; starved n = 18 VNCs. p = 4.56 × 10^−18^. Bar graphs represent mean ± SEM. (F–J) Rescue of NSC reactivation in *inx1* mutants by glial expression of *dIlp6*. Anterior up, dorsal view. (F′–H′) Higher magnification. Ventral views. NSC nuclei, green (Deadpan); Cell cortices, red (Discs Large); Phosphohistone H3, gray. (I) Quantification of brain volume rescue. A one-way Anova test was performed. ^∗∗∗^p < 0.05. Control n = 10 brains; *inx1*^*ogrejNL3*^*; glia > lacZ* n = 12 brains; *inx1*^*ogrejNL3*^*; glia > dIlp6* n = 10 brains. For control versus mutant, p = 8.68 × 10^−12^. For mutant versus rescue, p = 2.01 × 10^−2^. For control versus rescue, p = 0.12. (J) Quantification of NSC diameter rescue. A one-way Anova test was performed. ^∗∗∗^p < 0.05. Control n = 182 NSCs (10 VNCs); *inx1*^*ogrejNL3*^*; glia > lacZ*; n = 198 NSCs (12 VNCs); *inx1*^*ogrejNL3*^*; glia > dIlp6*; n = 219 NSCs (10 VNCs). For control versus mutant, p = 2.30 × 10^−42^. For mutant versus rescue, p = 1.84 × 10^−42^. For control versus rescue, p = 0.21. See also Figure S4.

We assayed whether gap junction proteins within the BBB are required for insulin signaling. We found a significant decrease in *dIlp6* transcription after knocking down both *inx1* and *inx2* in the glia (Figure S4A). We next assayed dIlp6 secretion. In the absence of an effective dIlp6 antiserum, we expressed a tagged, functional version of dIlp6 (dIlp6-FLAG) in the BBB glia. We found that dIlp6 secretion from the BBB glia was strongly impaired in *inx1* loss-of-function mutants (Figures 4A, 4B, and 4D). Secretion of dIlp6 was similarly impaired upon starvation (Figures 4A, 4C, and 4E). Therefore, both the expression and secretion of dIlp6 are regulated by nutrition and depend on gap junction proteins.

NSC reactivation in *inx* mutants was rescued by forced expression of *dIlp6* in glia (Figures 4F–4H′, see arrowheads), as shown by the recovery of brain volume (80% of the brains, Figure 4I) and of NSC diameter (Figure 4J). Direct activation of the PI3K/Akt pathway in NSCs also resulted in rescue of brain volume and NSC enlargement and entry into mitosis (Figures S4B–S4C′). We conclude that gap junction proteins in the BBB glia are required to activate insulin signaling and induce NSC reactivation.

### Gap Junctions Coordinate Calcium Oscillations in the BBB Glia

Secretion of insulin by the pancreas is induced by glucose, leading to synchronized calcium oscillations within gap junction-coupled beta cells and insulin exocytosis (MacDonald and Rorsman, 2006). Gap junctions enable the passage of secondary messengers that either trigger the release of calcium from intracellular stores or the influx of calcium from the extracellular environment (Laude and Simpson, 2009, Leybaert and Sanderson, 2012, Orellana et al., 2012, Skupin and Thurley, 2012). Blocking gap junctions inhibits coordinated intercellular calcium signaling (Leybaert and Sanderson, 2012, Orellana et al., 2012). Gap junction proteins are thus an important means of transmitting calcium waves.

**Figure 5:**
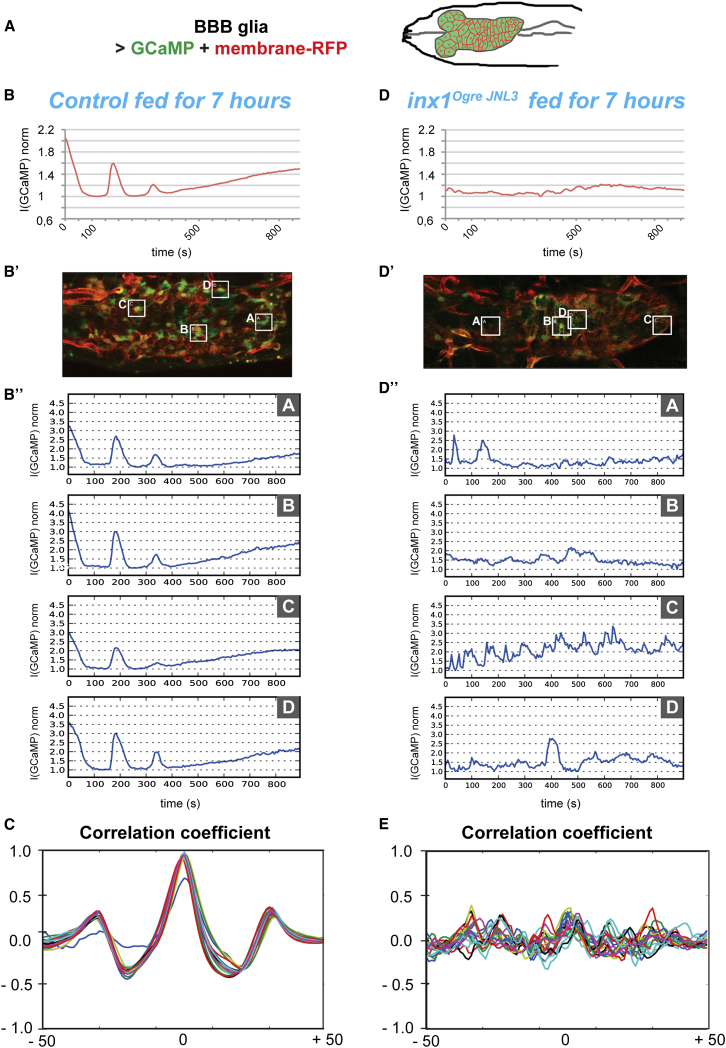
Coordinated Calcium Oscillations in the BBB Glia Depend on Gap Junctions (A) GCaMP3 and a membrane marker were driven specifically in the BBB glia (*moody-Gal4*, *UAS-mCD8-RFP*, *UAS-GCaMP3*). (B–B′′) Calcium oscillation in BBB glia at ALH7 in one control brain. (B) GCaMP3 normalized mean intensity over time for the entire plane. (B′) Regions of interest (ROI, A–D) at time 0. (B′′) GCaMP3 mean intensity over time for four different ROIs across the VNC. (C) Graph of the correlation coefficient for a fed larva before NSC enlargement. Twenty ROIs were chosen at random within the BBB glia and the plots of their normalized calcium intensity were correlated against each other. A correlation coefficient of 1 indicates perfect correlation. (D–D′′) Calcium oscillation in BBB glia of *inx1* mutant (*inx1*^*ogrejNL3*^) in one brain. (D) GCaMP3 normalized mean intensity over time for the entire plane. (D′) ROIs (A–D) at time 0. (D′′) GCaMP3 mean intensity over time for four different ROIS. (E) Graph of the correlation coefficient for an *inx1*^*ogrejNL3*^ mutant. Twenty ROIs were chosen at random within the BBB glia. See Experimental Procedures for details of the imaging and data processing. Anterior to the left. See also Figure S5, and Movies S1 and S2.

To investigate whether calcium signaling plays a role in gap junction-mediated NSC reactivation, we expressed a calcium sensor, GCaMP3 (Tian et al., 2009), in the BBB glia (Figure 5A; Figures S5A–S5C) of living larvae. Before reactivation (ALH7) the BBB glia of feeding larvae exhibited clear calcium oscillations (Movie S1; Figure 5B). The BBB glia pulsed simultaneously, suggesting that calcium oscillations are coordinated across the entire CNS. Individual cell tracking showed that glial calcium oscillations exhibited striking synchrony (Figures 5B′ and 5B′′) in all brains analyzed (n = 15, six are displayed; Figure S5D).

To further assess the extent of calcium oscillation coordination within the BBB glia under these conditions, we performed correlation analysis (see Experimental Procedures) for 20 regions of interest (ROI) chosen at random within the BBB glial layer (Figures 5C, S5E, and S5E′). In fed larvae before reactivation, the central correlation peak (coefficient 1) demonstrates the synchronicity of calcium oscillations within the BBB layer. Additional peaks on each side reveal that this synchronicity repeats (see Experimental Procedures for details).

Next, we monitored calcium dynamics in *inx1* mutants. None of the mutant brains (n = 11) showed coordinated calcium oscillations (Movie S2; Figure 5D; Figure S5F). Instead, BBB glial cells pulse independently, with no coordination between neighboring cells (Figures 5D′ and 5D′′). We conclude that Inx1/Inx2 gap junctions are required to coordinate synchronous calcium oscillations within the BBB glia. In accordance with our observation, the graph of correlation coefficient for *inx1* mutants established the total absence of synchronicity between BBB glial cells (Figures 5E, S5G, and S5G′), showing that gap junctions are required for propagating calcium oscillations within the BBB.

### Calcium Oscillations in the BBB Glia Respond to Nutritional Status

**Figure 6:**
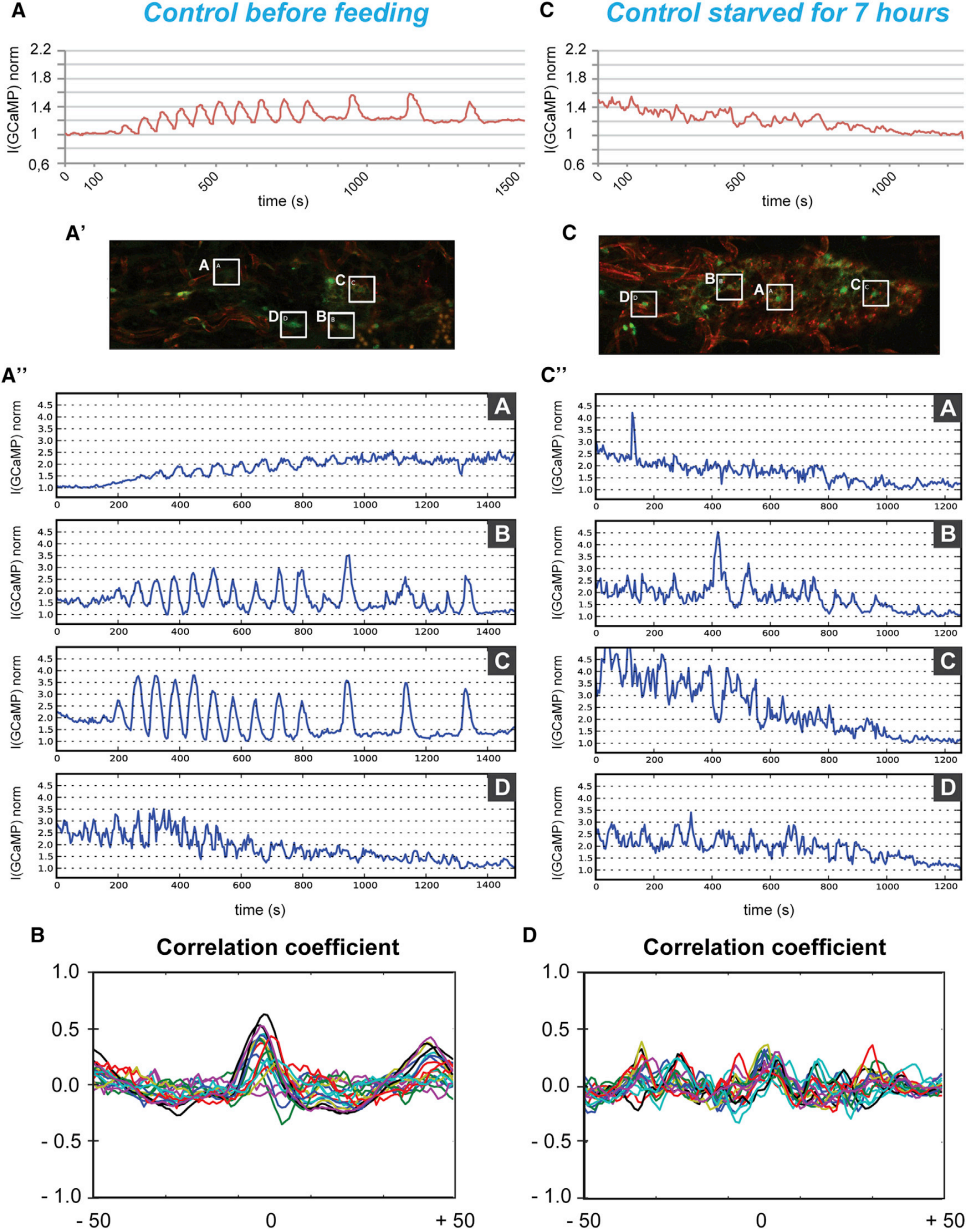
Coordinated Calcium Oscillations in the BBB Glia Depend on Nutritional State (A–A′′) Calcium oscillation in BBB glia of a newly hatched control larva (ALH0). (A) GCaMP3 normalized mean intensity over time for the entire plane. (A′) ROIs (A–D) at time 0. (A′′) GCaMP3 mean intensity over time for four different ROIs. (B) Graph of the correlation coefficient for a newly hatched control larva (ALH0). Twenty ROIs were chosen at random within the BBB glia. (C–C′′) Calcium oscillation in BBB glia in the brain of a starved larva. (C) GCaMP3 normalized mean intensity over time for the entire plane. (C′) ROIs (A–D) at time 0. (C′′) GCaMP3 mean intensity over time for four different ROIs. (D) Graph of the correlation coefficient for a starved larva. Twenty ROIs were chosen at random within the BBB glia. See Experimental Procedures for details of the imaging and data processing. Anterior to the left. See also Figure S6, and Movies S3 and S4.

To assess whether calcium oscillations in the BBB are induced by a nutritional stimulus, we first assayed calcium dynamics in the BBB glia of newly hatched larvae (ALH0), before they started to feed. The calcium oscillations differed both in extent and frequency from those seen in fed larvae (n = 9; Movie S3; Figures 6A–6A′′; Figure S6A). Correlation analysis revealed a partial coordination within the BBB glia (central correlation peak with a coefficient of 0.5, Figures 6B, S6B, and S6B′), strengthening the idea that nutrition is important for extending and establishing robust calcium synchronicity in the BBB.

Next, we assessed calcium dynamics after starvation, specifically the absence of essential amino acids. Calcium dynamics in the BBB of starved larvae resembled *inx1* mutant brains (Movie S4; Figure 6C). Synchronous calcium oscillations were completely abolished in all brains examined (n = 19, Figure S6C). Individual BBB cells displayed some calcium pulses (Figures 6C′ and 6C′′), but with different profiles to those seen in fed larvae. In addition, correlation analysis of starved larvae showed very weak synchronicity (Figures 6D, S6D, and S6D′). This suggests that the synchronous, nutrition-dependent, calcium oscillations are lost upon starvation. Importantly, neither Inx1 nor Inx2 is lost under starvation conditions (Figure S2E). We conclude that nutrition, in particular essential amino acids, shape calcium dynamics. Upon feeding, calcium oscillations are amplified and synchronized across the BBB.

### The IP3 Pathway and BBB Membrane Polarization Control Neural Stem Cell Reactivation

**Figure 7:**
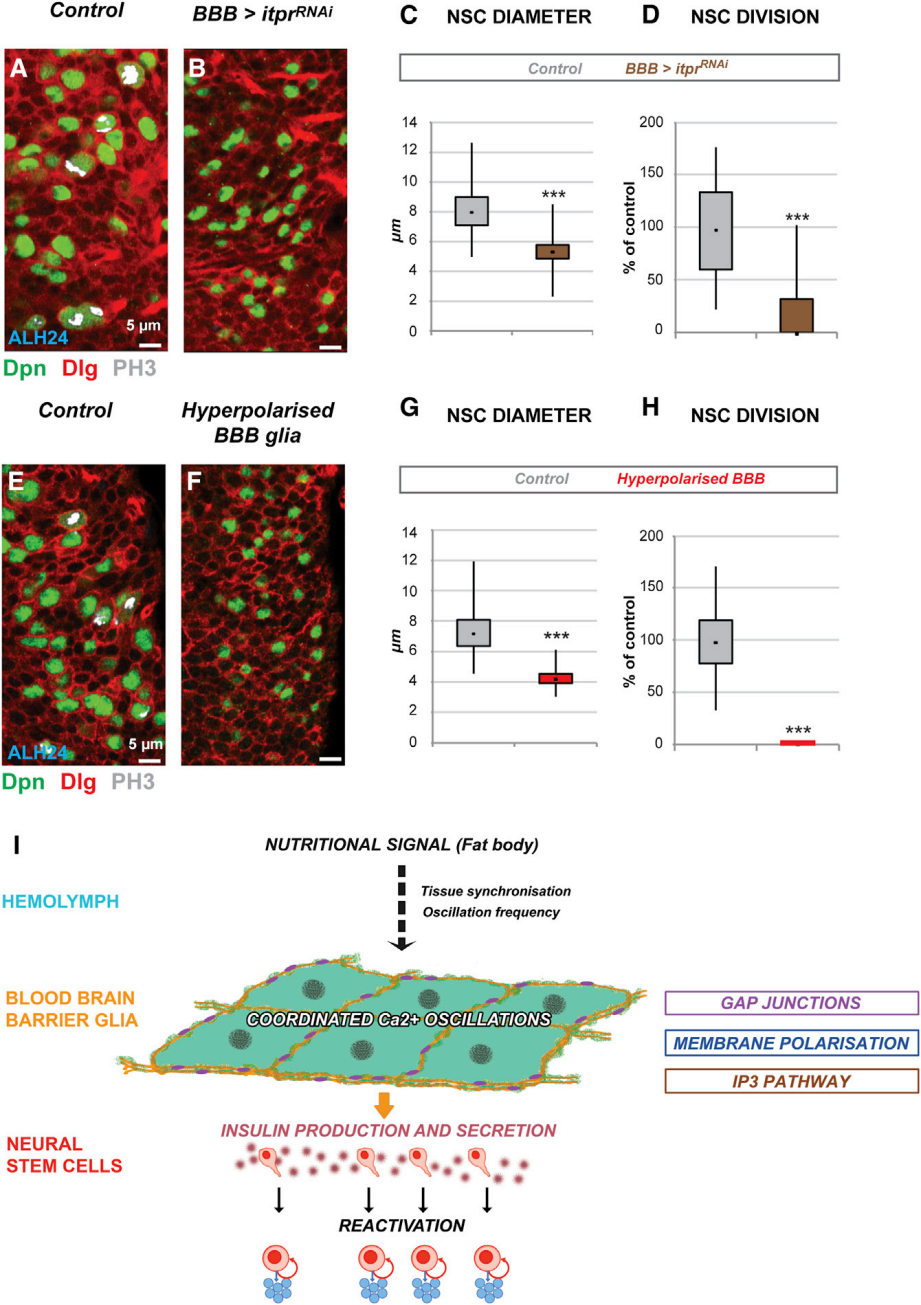
Calcium Signaling in the BBB Glia Is Required for NSC Reactivation (A–D) NSC reactivation depends on the insitol-triphosphate pathway. Knocking down the insitol-triphosphate receptor in the BBB glia only (*mdr65-GAL4 > itpr*^*RNAi*^) impairs NSC enlargement and proliferation (compare B to A). (C and D) Quantification of NSC (C) diameter and (D) proliferation; whisker plots. (C) ^∗∗∗^p < 0.05. Two-sided Student’s test. Average and standard deviation were calculated from two biological replicates. Control n = 352 NSCs (22 VNC); *BBB glia > itpr*^*RNAi*^ n = 358 NSCs (22 VNC); p = 2.31 × 10^−116^. (D) ^∗∗∗^p < 0.05. Two-sided Student’s t test. Average and SD were calculated from two biological replicates. Control n = 22 VNCs; *BBB glia > itpr*^*RNAi*^ n = 22 VNCs. p = 1.03 × 10^−7^ (unequal variance test). (E–H) NSC enlargement and proliferation are blocked upon BBB membrane hyperpolarization (*BBB glia > UAS-kir2.1*.) (compare F to E). (G and H) Quantification of NSC (G) diameter and (H) proliferation; whisker plots. (G) ^∗∗∗^p < 0.05. Two-sided Student’s t test. Average and SD were calculated from two biological replicates. Control n = 466 NSCs (22 VNC); BBB hyperpolarization n = 366 NSCs (16 VNC). p = 3.30 × 10^−110^. (H) ^∗∗∗^p < 0.05. Two-sided Student’s test. Average and SD were calculated from three biological replicates. Control n = 33 VNCs; BBB hyperpolarization n = 26 VNCs. p = 7.88 × 10^−23^ (unequal variance test). All images are anterior up, ventral view. NSC nuclei, green (Deadpan, Dpn); Cell cortices, red (Discs Large, Dlg); PH3, gray (phosphohistone H3). (I) Model of the role of the BBB glia in nutrition-dependent NSC reactivation. Nutrition induces calcium oscillations, which encode the nutritional message required for NSC reactivation, in the BBB. Inx1/Inx2 heteromeric channels are necessary to synchronize calcium oscillations throughout the BBB and regulate insulin production and secretion. See also Figure S7.

We assayed whether glial calcium oscillations arise from the release of intracellular calcium, or from the influx of extracellular calcium, and how these influence NSC reactivation. First, we assessed the importance of the inositol-triphosphate (IP3) pathway. The IP3 pathway triggers calcium release from intracellular stores (Berridge, 2009). Stimulation of G-coupled receptors by a wide range of signals activates phospholipase C, leading to the production of IP3 from cleaved PIP2. IP3 then binds to its receptor (Ins3PR), a ligand-gated Ca^2+^ channel found on the surface of the ER, releasing intracellular calcium. We knocked down Ins3PR (*itpr* in *Drosophila*) in the BBB glia by RNAi (Chorna and Hasan, 2012). Both NSC enlargement and proliferation were strongly impaired (Figures 7A–7D). We conclude that NSC reactivation depends on IP3-mediated release of calcium from intracellular stores.

Next, we assessed the importance of calcium influx. Membrane depolarization triggers the entry of extracellular calcium via voltage-gated calcium channels (Catterall, 2011, Leybaert and Sanderson, 2012), whereas hyperpolarization prevents it. We hyperpolarized BBB membranes by expressing the inward-rectifying potassium channel, kir2.1 (Baines et al., 2001). BBB hyperpolarization blocks NSC reactivation dramatically, as revealed by the complete failure of both enlargement and mitotic re-entry (Figures 7E–7H). Interestingly, the mushroom body neuroblasts, a small group of central brain NSCs that do not undergo quiescence and reactivation, are not affected by BBB hyperpolarization, suggesting that nutrition-dependent NSC reactivation is specifically affected (Figures S7A and S7B, see arrowheads). BBB hyperpolarization both decreases *dIlp6* mRNA levels (Figure S7C) and dIlp6 secretion (Figures S7D and S7E), similar to what is seen during starvation or gap junction loss-of-function (see Figure S4A and Figures 4A–4E). In support of the role of calcium oscillations in reactivating NSCs, we found that overexpressing the calcium-binding protein, calmodulin, prevents NSC reactivation (Figures S7C–S7F). Our results show that intracellular and extracellular calcium both contribute to NSC reactivation.

## Discussion

### Gap Junctions Are Required in the BBB for NSC Reactivation

The nutrient-dependent reactivation of NSCs in the *Drosophila* brain demonstrates how NSCs can adapt to environmental changes to fulfil the needs of the organism. Here, we show that gap junction proteins within the BBB glia are required for insulin expression and secretion, a prerequisite for NSC exit from quiescence (Figure 7I). We demonstrate that gap junction proteins coordinate glial calcium oscillations that are required for NSC reactivation. Both intracellular calcium stores and calcium influx contribute to reactivation. Membrane depolarization is known to regulate exocytosis via calcium signaling (Fridlyand and Philipson, 2011, Stojilkovic, 2012, Südhof, 2012), which controls stimulus-secretion coupling in secretory cells, such as in endocrine cells (Dolenšek et al., 2011, Géminard et al., 2009, Stojilkovic, 2012, Südhof, 2012). We show that conditions that block calcium oscillation in the BBB glia (the loss of gap junction proteins, starvation) also impair insulin secretion.

### Mechanisms of Insulin Production and Secretion in *Drosophila* and Vertebrates

The sequence of events leading to glial secretion of insulin bears a striking resemblance to the diet-induced release of insulin by the beta cells of the pancreas (MacDonald and Rorsman, 2006). In the pancreas, a nutritional stimulus is sensed by gap junction-coupled beta cells, inducing depolarization resulting in synchronized calcium oscillation and insulin secretion. Loss of gap junction coupling results in uncoordinated calcium pulses. In *Drosophila inx* loss of function mutants, individual subperineurial BBB glial cells oscillate independently of one another. Compared to starvation, in which the nutritional signal is absent and NSC reactivation cannot occur, the scattered signals from individual BBB cells are able to induce delayed, asynchronous, reactivation in a small number of NSCs (see PH3 positive NSCs in Figures 1C′–1D′). We propose that gap junction function within the BBB enables glial insulin release to reach a threshold high enough to trigger NSC reactivation throughout the central nervous system.

In both beta cells and BBB cells, membrane depolarization is crucial for generating calcium oscillations. Failure to depolarize or an active block to depolarization prevents insulin release. However, sustained depolarization of β cells can lead to desensitization and a decline in insulin release (Willenborg et al., 2012). Interestingly, we find that forced depolarization of BBB glia only mildly enhances NSC reactivation (data not shown). This could be due to desensitization or it may be that the system is already maximally active.

Insulin mRNA levels are decreased after gap junction knockdown, both in pancreatic islets (Bosco et al., 2011) and in the BBB glia. In both cases it remains to be determined if calcium oscillations can directly affect gene expression, as has been shown in other systems (Alonso and García-Sancho, 2011).

Insulin produced by the pancreas is distributed via the circulatory system, whereas glial insulin is secreted locally, directly to underlying NSCs. Glial insulin signaling is thus contained within the brain, enabling local, differential regulation of this organ. The BBB acts both as a niche and as a protective barrier, providing specific factors directly to the stem cell while shielding the brain from unwelcome systemic regulation. In the context of NSC reactivation, these two roles are conveniently complementary. In the vertebrate BBB, similar functions may be split between endothelial cells and astrocytic glia. The vascular endothelium provides the barrier function, while astrocytic glia have a regulatory role in sensing and adjusting barrier permeability to various stimuli (Daneman, 2012). BBB endothelial cells can secrete cytokines, chemokines, and prostaglandins, suggesting that the BBB behaves like an endocrine tissue (Banks, 2012). Interestingly, calcium oscillations have been observed in cultured vertebrate BBB endothelial cells, but their function is largely unknown (De Bock et al., 2013).

### Gap Junction Function in Neural Stem Cells

Gap junction communication can influence stem cell behavior by directly coupling stem cells to each other or to supporting cells, such as found in a stem cell niche. In the brain, connexon-mediated communication has been reported to occur between progenitor cells, within astrocytic networks, and between radial glia and neurons or progenitor cells and astrocytes (Giaume et al., 2010, Lacar et al., 2011, Nakase and Naus, 2004, Lo Turco and Kriegstein, 1991). The proliferation of neural progenitors and the formation of cortical layers in the mouse brain depend on an intercellular gap junction network (Malmersjö et al., 2013), and grafted human NSCs integrate into organotypic cultures through connexin coupling (Jäderstad et al., 2010). Here, we show that gap junction function within a niche, the BBB, can also influence NSC behavior.

The *Drosophila* BBB is a protective and selective barrier as well as a signaling center that orchestrates major developmental and physiological events. Here we show that gap junction communication enables cells within the BBB to act as a concerted unit, leading to coordinated calcium signaling and insulin release. Similarities between the BBB in vertebrates and invertebrates suggest that our findings are likely to have broader significance.

## Experimental Procedures

### Genetics

The RNAi and GAL4 drivers used in this study are listed in Tables S1 and S3. *inx1*^*ogrejNL3*^ and *moody-GAL4* were kind gifts of P. Phelan and R. Bainton, respectively. All RNAi experiments were conducted at 29°C. All glial knockdowns were performed with *repo-GAL4* as driver, unless stated otherwise. The BBB driver was either *moody-GAL4* or *mdr65-GAL4* (see for each experiment).

### Statistics

Bar graphs were generated using the mean and SEM for each sample. Error bars represent SEM. Center values are averages. Student’s t test was performed using a threshold of p < 0.05 (confidence interval of 95%), represented by ^∗∗∗^. All tests performed in this study were two-sided and with samples of same variance, unless stated otherwise.

For rescue of *inx1* mutant by glial expression of *dIlp6*, we used a one-way Anova test, with p < 0.05 represented by ^∗∗∗^. Whisker plots were drawn using the minimum, quartile 1, median, quartile 3, and maximum of each condition’s sample. In addition, a Student’s t test based on a sample’s average and SD was used to generate p values with the same criteria as above. *n* refers to the total number of samples for all biological or technical replicates. NSC numbers were determined using measurement scripts in Volocity software (PerkinElmer).

### Temperature Shift Experiments

We used *tub-Gal80*^*ts*^*, inx1*^*RNA*i^ *; repo-GAL4* to achieve conditional expression of *inx1*. At 25°C (permissive temperature for *Gal80*^*ts*^), larvae showed a wild-type phenotype, but at 30°C (restrictive temperature for *Gal80*^*ts*^), all larvae showed a mutant phenotype: lack of NSC reactivation and clear reduction in brain volume. In further experiments, we scored mutant phenotypes by brain volume only in third instar larvae. Twelve larvae were scored for each condition. In single temperature-shift experiments, 1 hr embryos or 1 hr first instar larvae were grown at a given temperature and shifted once. We tested 6 or 24 hr temporal windows.

### dIlp6 Secretion Experiments

We coexpressed a tagged version of dIlp6 (dIlp6-FLAG) and a membrane marker (mCD8-RFP) in the BBB glia alone, in controls (genotype *moody-GAL4, UAS-mCD8-RFP, UAS-dIlp6-FLAG*) and *inx1* mutants (genotype *inx1*^*ogrejNL3*^*/Y; moody-GAL4, UAS-mCD8-RFP, UAS-dIlp6-FLAG*). Larvae were starved as described in the Supplemental Experimental Procedures. All brains were imaged using the same confocal settings for dIlp6-FLAG. For each VNC, the total dIlp6 intensity (T) was calculated using Volocity software, using a fixed intensity threshold. Retained dIlp6 intensity (R) was then measured as the dIlp6 signal (same threshold as previously) colocalizing with the BBB membrane (RFP signal). The threshold for the RFP signal was determined manually for each brain, to ensure selection of the whole membrane. Due to the flatness of the BBB glia, the membrane signal represents most of the cells, although some cytoplasmic signal could be missed. The percentage of secreted dIlp6 was calculated as percent secreted dIlp6 = (1 − R/T) × 100.

### Membrane Polarization Experiments

*moody-GAL4*, *UAS-kir2.1-GFP; tubulin-GAL80*^*ts*^ flies were grown at permissive temperature until hatching, then switched to 30°C from ALH0 to ALH24.

### Calcium Imaging

Two different conditions were analyzed: control (genotype *FM7*, *Dfd GMR YFP/Y; moody-GAL4*, *UAS-mCD8-RFP*, *UAS-GCaMP3*) and *inx1* mutant (genotype *inx1*^*ogrejNL3*^*/Y; moody-GAL4*, *UAS-mCD8-RFP*, *UAS-GCaMP3*). Larvae were raised and staged at 29°C, then fed or starved as described in the Supplemental Experimental Procedures. Larvae were placed in a drop of Voltalef oil on a 22 mm diameter Welco dish, ventral side down. A 13 mm diameter round coverslip was placed tilted on top and lowered until the larva was immobilized.

Mounted larvae were imaged with a 40× oil immersion objective on an Olympus inverted FV1000. One focal plane encompassing as much as possible of the GCaMP signal was imaged. GCaMP and mCD8-RFP were imaged simultaneously because we did not detect any bleed-through.

### Calcium Data Processing

To measure the mean intensity of the whole plane over time, movies were analyzed in Volocity. The mean intensity was measured for each point both for GCAMP3 (channel 1, C1) and mCD8-RFP signals (channel 2, C2). We normalized changes in GCaMP3 intensity against changes in mCD8-RFP intensities (accounting mainly for loss of focal plane due to larval movement), and then normalized the ratio by its minimum over the whole movie, so that the baseline for all movies was always 1. The normalized GCaMP3 signal is then: I(GCaMP) norm = (C1/C2) / (C1/C2)_min_. This normalized ratio was plotted over time using spreadsheet software.

### Calcium Tracking

To follow the behavior of calcium in different regions of the brain to check for oscillation coordination, we tracked selected ROIs independently.

For this specific purpose, a tailored python script was developed (F.N. Murphy). ROIs were selected manually in the first frame of the movie, and their central coordinates recorded. The script then tracked the ROIs as they moved during the movie. A 64 × 64 pixel tracking window was centered on each ROI, and a 32 × 32 pixel subwindow in the middle of each tracking window was used for measuring intensity values. The tracking operations were performed on the relatively stable red channel (cell membrane) rather than the more dynamic green channel (the calcium signal to be measured). The script moves the tracking window by calculating the correlation between the window region from one frame to the next. The location of the peak of the correlation function indicates how far and in which direction to move the tracking window to keep it centered on the ROI. To reduce tracking drift due to rounding errors, times-two upsampling was used. To further improve tracking accuracy, the image was low pass filtered to remove noise. The upsampling and filtering were accomplished simultaneously by taking the product of the fast Fourier transforms of the tracking window and extracting the low-frequency coefficients then zero padding before taking the inverse fast Fourier transforms. The intensity measurements were performed by summing the pixel values within the 32 × 32 pixel measuring window for both the red and green channels. Normalization was then applied as described above.

### Correlation Analysis

The intensity signal for each ROI (computed and normalized as described above) is processed to remove baseline wander. The baseline is calculated using a 50 sample running average filter and then subtracted out from the signal. The remaining signal is then scaled so that the sum of the squares of the samples is one. The signal with the largest positive excursion is selected as a reference. Each remaining ROI signal is correlated with the reference for shifts of −50 to +50 samples. The result is a correlation coefficient indicating the similarity of the signals. If a signal is identical to the reference, the correlation coefficient will peak at +1 for a shift of 0. Multiple peaks will be observed if the signal is periodic. If a signal is uncorrelated with the reference, then the correlation coefficient will take small random values.

## Supplemental Information

Document S1. Supplemental Experimental Information, Figures S1–S7, and Tables S1–S3

Movie S1. The Blood-Brain Barrier Glia Show Spontaneous Synchronized Calcium Oscillations, Related to Figure 5 Live imaging of the BBB of a young (ALH7) fed control larva. Calcium, green (GCaMP); membrane, red (mCD8-RFP). A duration of 1 s in the movie corresponds to 70 s during the experiment. The total length of the recording is approximately 15 min.

Movie S2. Coordinated Calcium Oscillations Are Lost When BBB Gap Junctions Are Disrupted, Related to Figure 5 Live imaging of the BBB of a young (ALH7) *inx1*^*ogre jNL3*^ larva. Calcium, green (GCaMP); membrane, red (mCD8-RFP). A duration of 1 s in the movie corresponds to 70 s during the experiment. The total length of the recording is approximately 15 min.

Movie S3. Nutrition Shapes and Synchronizes Calcium Oscillations in the Blood-Brain Barrier Glia, Related to Figure 6 Live imaging of the BBB of a just-hatched (ALH0) control larva. Calcium, green (GCaMP); membrane, red (mCD8-RFP). A duration of 1 s in the movie corresponds to 70 s during the experiment. The total length of the recording is approximately 25 min.

Movie S4. Starvation Prevents the Synchronization of Calcium Oscillations in the Blood-Brain Barrier Glia, Related to Figure 6 Live imaging of the BBB of a young (ALH7) starved control larva. Calcium, green (GCaMP); membrane, red (mCD8-RFP). A duration of 1 s in the movie corresponds to 70 s during the experiment. The total length of the recording is approximately 21 min.

Document S2. Article plus Supplemental Information
