# Supplementary material for: Gap Junction Proteins in the Blood-Brain Barrier Control Nutrient-Dependent Reactivation of Drosophila Neural Stem Cells
Source: Dev Cell. 2014 Aug 11;30(3):309–21. doi: 10.1016/j.devcel.2014.05.021 (PMC4139190; doi:10.1016/j.devcel.2014.05.021)
Supplement: Document S1. Supplemental Experimental Information, Figures S1–S7, and Tables S1–S3 [file mmc1.pdf]

Developmental Cell, Volume *30*

Supplemental Information

Gap Junction Proteins in the Blood-Brain Barrier Control Nutrient-Dependent Reactivation of *Drosophila* Neural Stem Cells

Pauline Spéder and Andrea H. Brand

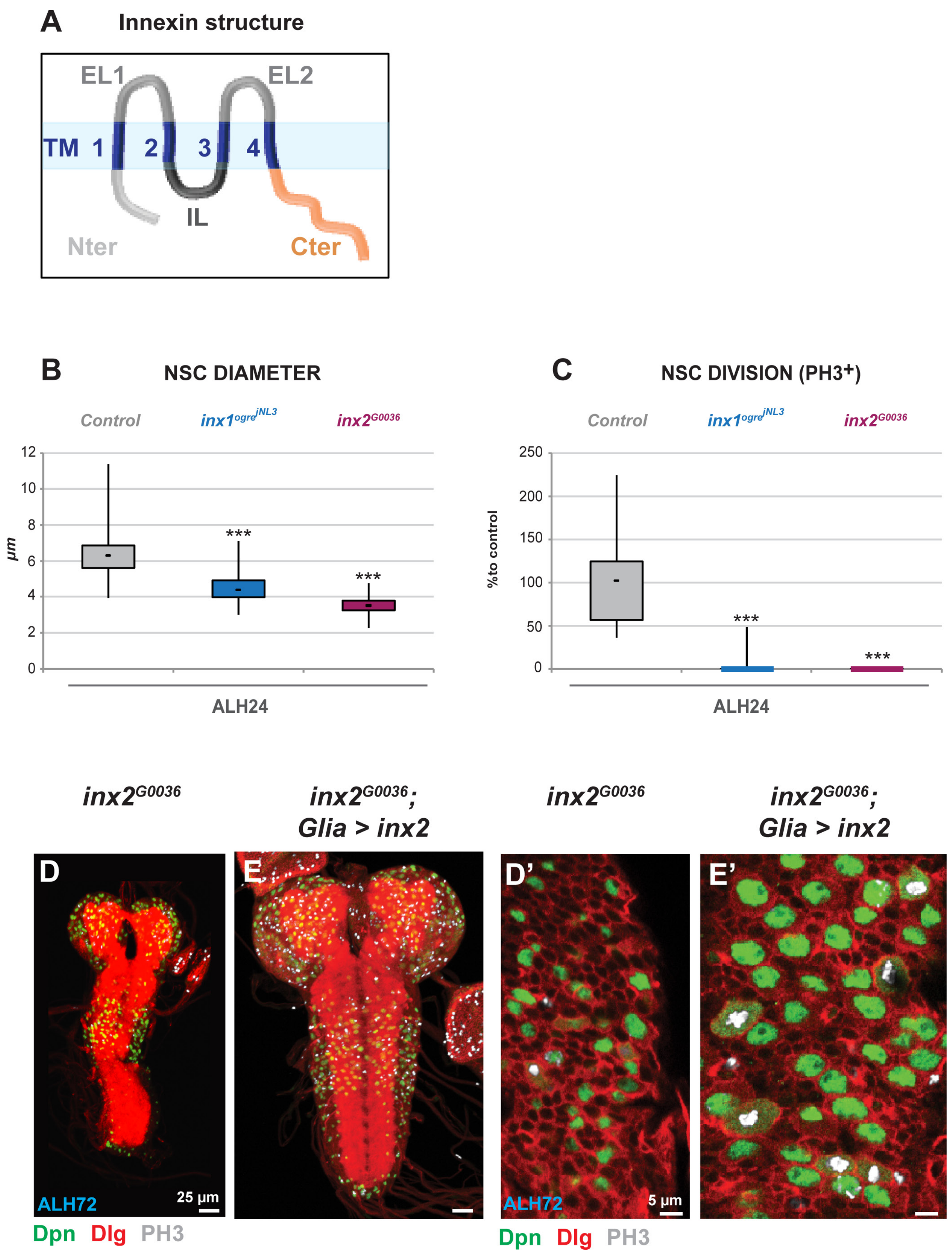
A
Innexin structure
EL1
EL2
TM 1
2
3
4
IL
Nter
Cter
B
NSC DIAMETER
Control
inx1ogre jNL3
inx2G0036
µm
12
10
8
6
4
2
0
***
***
ALH24
C
NSC DIVISION (PH3+)
Control
inx1ogre jNL3
inx2G0036
%to control
250
200
150
100
50
0
***
***
ALH24
inx2G0036
inx2G0036; Glia > inx2
inx2G0036
inx2G0036; Glia > inx2
D
E
D'
E'
ALH72
25 µm
ALH72
5 µm
Dpn Dlg PH3
Dpn Dlg PH3

Figure S1, related to Figure 1. Innexins are gap junction proteins required for NSC reactivation. (A) Schematic of Innexins. Inxs are comprised of a cytoplasmic N-terminal (Nter) tail, a C-terminal (Cter) tail, four transmembrane domains (TM1 to 4), two extracellular loops (EL1 and EL2) and one intracellulare loop (IL). **(B-C)** Quantification of NSC (B) diameter and (C) proliferation in *inx1* and *inx2* mutants. (B) ••• $p < 0.05$. Two-sided Student's test. Average and standard deviation were calculated from two biological replicates. Control n = 306 NSCs (16 VNC); $inx1^{ogrejNL3}$ n= 305 NSCs (16 VNCs); $inx2^{G0036}$ n = 214 NSCs (10 VNCs). $inx1^{ogrejNL3}$ $p = 1.23 \times 10^{-28}$; $inx2^{G0036}$ $p = 2.47 \times 10^{-78}$. (C) ••• $p < 0.05$. Two-sided Student's test. Average and standard deviation were calculated from two biological replicates. Control n = 16 VNCs; $inx1^{ogrejNL3}$ n =16 VNCs *;* $inx2^{G0036}$ n = 10 VNCs*.* $inx1^{ogrejNL3}$ $p = 7.78 \times 10^{-7}$. $inx2^{G0036}$ $p = 7.65 \times 10^{-7}$. (**D-E'**) Rescue of NSC reactivation in $inx2^{G0036}$ mutants by glial expression of *inx2*. (B-C) Anterior up, dorsal view. (B'-C') Higher magnification. Ventral views. NSC nuclei, green (Deadpan); Cell cortices, red (Discs Large); Phospho-histone H3, grey.

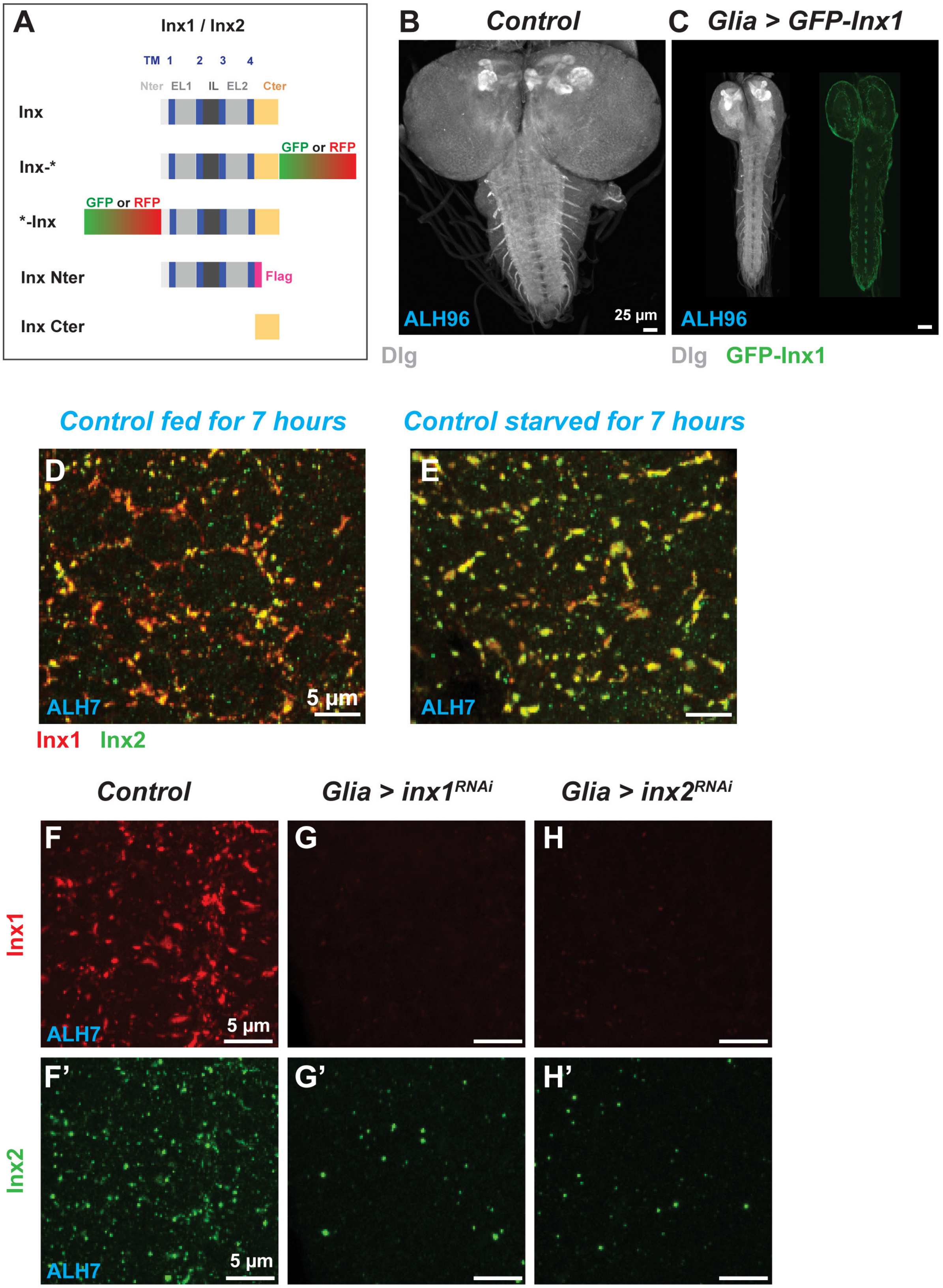
A
Inx1 / Inx2
TM 1 2 3 4
Nter EL1 IL EL2 Cter
Inx
Inx-*
GFP or RFP
*-Inx
GFP or RFP
Inx Nter
Flag
Inx Cter
B
Control
ALH96
25 µm
C
Glia > GFP-Inx1
ALH96
Dlg
Dlg GFP-Inx1
Control fed for 7 hours
Control starved for 7 hours
D
ALH7
5 µm
E
ALH7
Inx1 Inx2
Control
Glia > inx1RNAi
Glia > inx2RNAi
F
G
H
Inx1
ALH7
5 µm
F'
G'
H'
Inx2
ALH7
5 µm

Figure S2, related to Figure 2. Inx1 and Inx2 form heteromeric channels. (**A)** Five different constructs have been generated for Inx1 and Inx2. * represents GFP for Inx1 and RFP for Inx2. (**B-C**) Nter fusion acts as a dominant negative. Membrane, grey (Dlg); GFP-Inx1 , green. (**D-E**) Confocal images showing that Inx1 and Inx2 co-localisation is not lost under starvation. Inx1, red; Inx2 , green. Extended projection. (**F-H'**) Localisation of Inx1 (red) and Inx 2 (green) were assayed in glia when the reciprocal *inx* was knocked down by RNAi. Compare G and H with F and between them, and compare G' and H' with F' and between them. Notice the similarity between G and H, as well as between G' and H'.

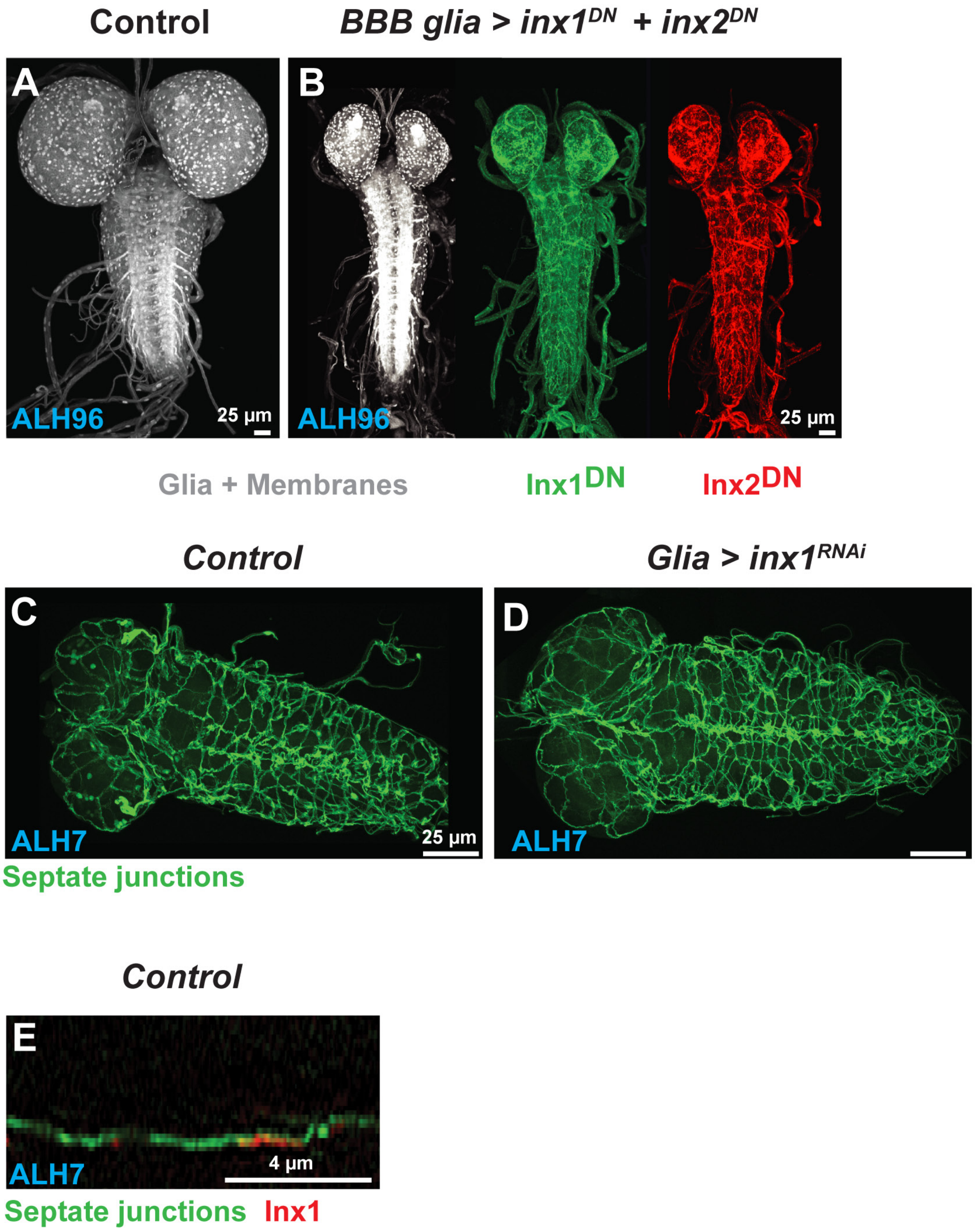
Control
BBB glia > inx1DN + inx2DN
A
ALH96
25 µm
B
ALH96
25 µm
Glia + Membranes
Inx1DN
Inx2DN
Control
Glia > inx1RNAi
C
ALH7
25 µm
D
ALH7
Septate junctions
Control
E
ALH7
4 µm
Septate junctions
Inx1

Figure S3, related to Figure 3. Inx1/Inx2 channels are required in the BBB glia for NSC reactivation. (A-B) Blocking *inx1* and *inx2* function in blood-brain barrier (BBB) glia phenocopies pan-glial knockdown. Glial nuclei, grey (Repo) ; Cell cortices, grey (Dlg); $Inx1^{DN}$ (GFP-Inx1), green ; $Inx2^{DN}$ (RFP-Inx2), red. **(C-D)** Septate junctions of the BBB glia are not affected in *inx1* knock down. Septate junction stainings (green) in the whole brain of control (**C**, *Lachesin ::GFP ; repo-GAL4*) and RNAi knockdown conditions (**D**, *Lachesin ::GFP,* $inx1^{RNAi}$*; repo-GAL4*). Maximal projection. (**E**) High-resolution (OMX) close-up of a septate junction in a control brain. Orthogonal section. Septate junction, green (*Lachesin::GFP*); Inx1, red.

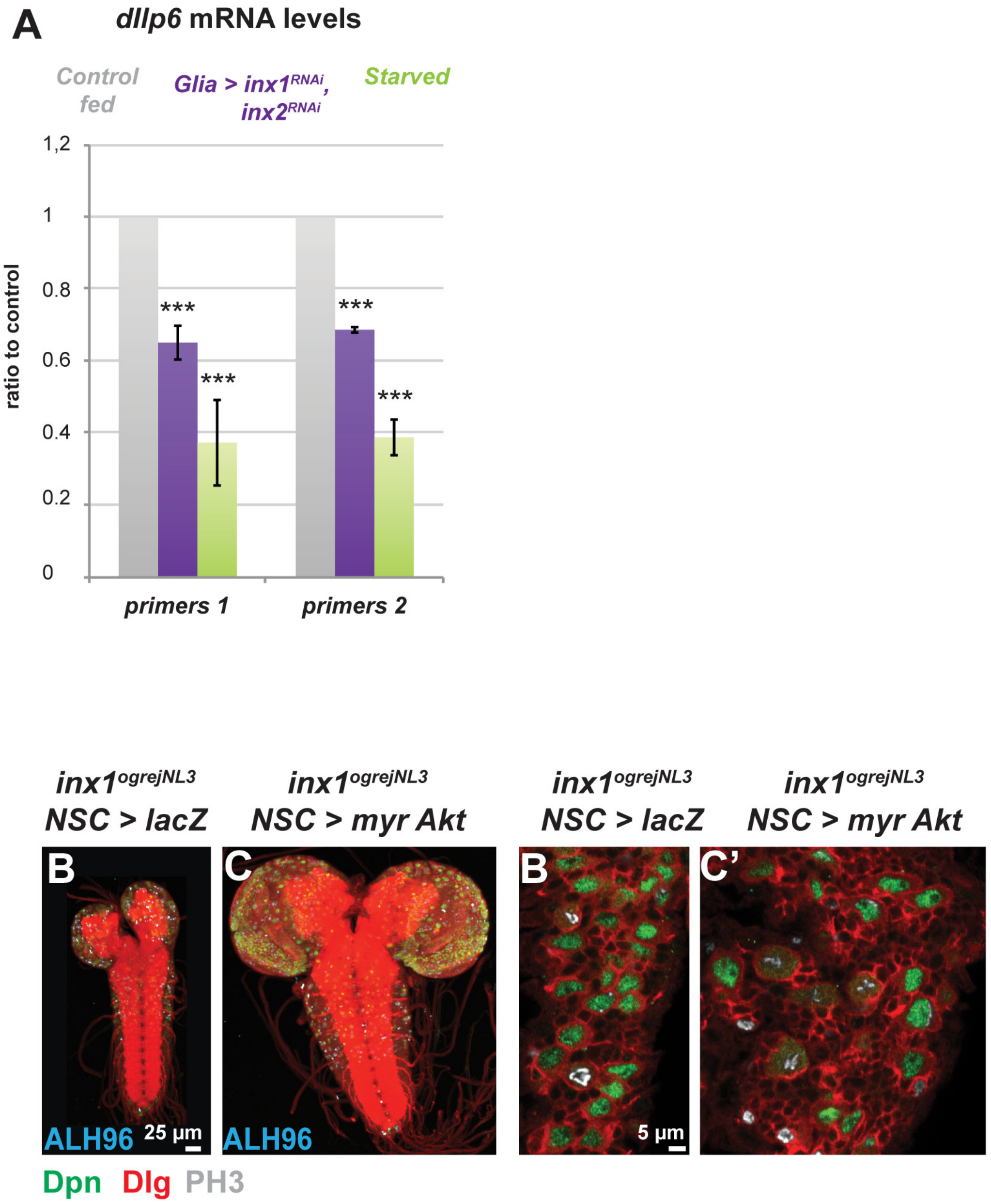
A
dIlp6 mRNA levels
Control fed
Glia > inx1RNAi, inx2RNAi
Starved
1,2
1
0.8
0.6
0.4
0.2
0
ratio to control
***
***
***
***
primers 1
primers 2
inx1ogrejNL3 NSC > lacZ
inx1ogrejNL3 NSC > myr Akt
inx1ogrejNL3 NSC > lacZ
inx1ogrejNL3 NSC > myr Akt
B
C
B
C'
ALH96
25 µm
ALH96
5 µm
Dpn Dlg PH3

Figure S4, related to Figure 4. Gap junctions control NSC reactivation through the insulin pathway. (**A**) Quantification of *dIlp6* transcript levels by q-pcr in glial knockdown of both *inx1* and *inx2*, compared to fed or starved controls. Two pairs of *dIlp6* primers were used. Three biological replicates. See Extended Experimental procedures for details. ••• $p < 0.05$. Two-sided Student's test. Primers 1: *Glia > inx1*^{RNAi}*, inx2*^{RNAi} : $p = 1.74 \times 10^{-3}$; control starved : $p = 7.96 \times 10^{-4}$. Primers 2: *Glia > inx1*^{RNAi}*, inx2*^{RNAi} : $p = 2.08 \times 10^{-6}$; control starved : $p = 2.83 \times 10^{-5}$. Bar graphs represent mean ± SEM. (**B-C**) Rescue of NSC reactivation in *inx1* mutants by ectopic expression of a constitutive activated form of Akt (myr-Akt) in NSCs. Anterior up, dorsal view (**B'-C'**) Higher magnification. Ventral views. NSC nuclei, green (Deadpan); Cell cortices, red (Discs Large); Phospho-histone H3, grey.

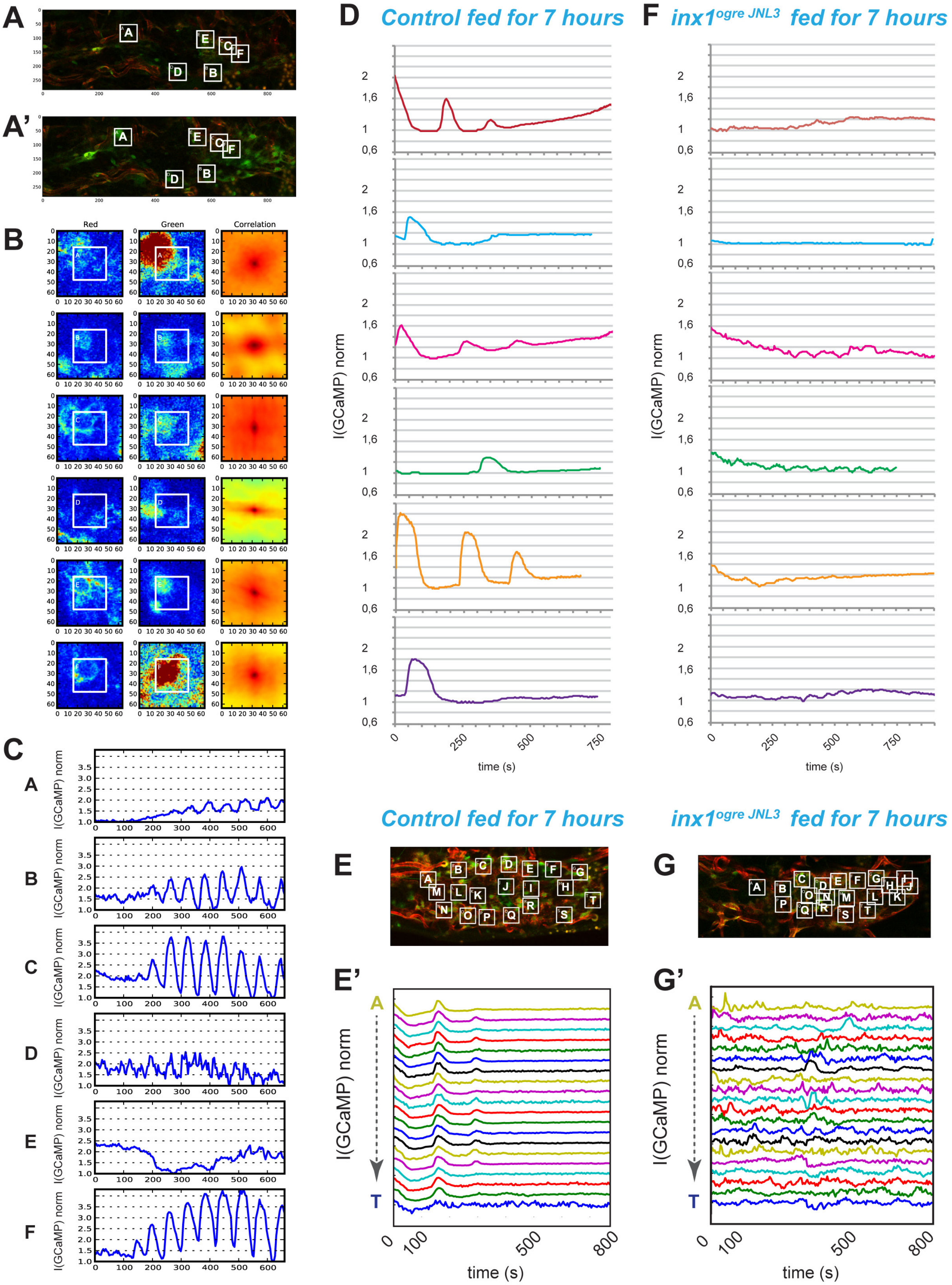
A
A'
B
Red
Green
Correlation
C
I(GCaMP) norm
D
Control fed for 7 hours
F
inx1ogre JNL3 fed for 7 hours
I(GCaMP) norm
time (s)
E
Control fed for 7 hours
G
inx1ogre JNL3 fed for 7 hours
E'
G'
A
T
time (s)

Figure S5, related to Figure 5. Analysis of calcium oscillations in control and *inx1* mutant conditions. (A-C) Calcium tracking script. (A-A'), Snapshots of the ROI selector at time 0 (A) and 160 frames after (A'). Each selected ROI is represented by a square. (B) Snapshot of the correlation window. (C) Snapshot of live plots of normalised GCaMP3 intensities over time. (**D**) Normalised GCaMP3 intensities plotted over time for 6 brains of control fed for 7 hours. **(E-E')** Correlation analysis for a fed larva before NSC enlargement. (E) Map of the 20 ROIs at time 0. (E') Normalised calcium intensity over time for the 20 ROIs. **(F)** Normalised GCaMP3 intensities plotted over time for 6 brains of $inx1^{ogrejNL3}$ fed for 7 hours. **(G-G')** Correlation analysis for an $inx1^{ogrejNL3}$ mutant. (G) Map of the 20 ROIs at time 0. (G') Normalised calcium intensity over time for the 20 ROIs.

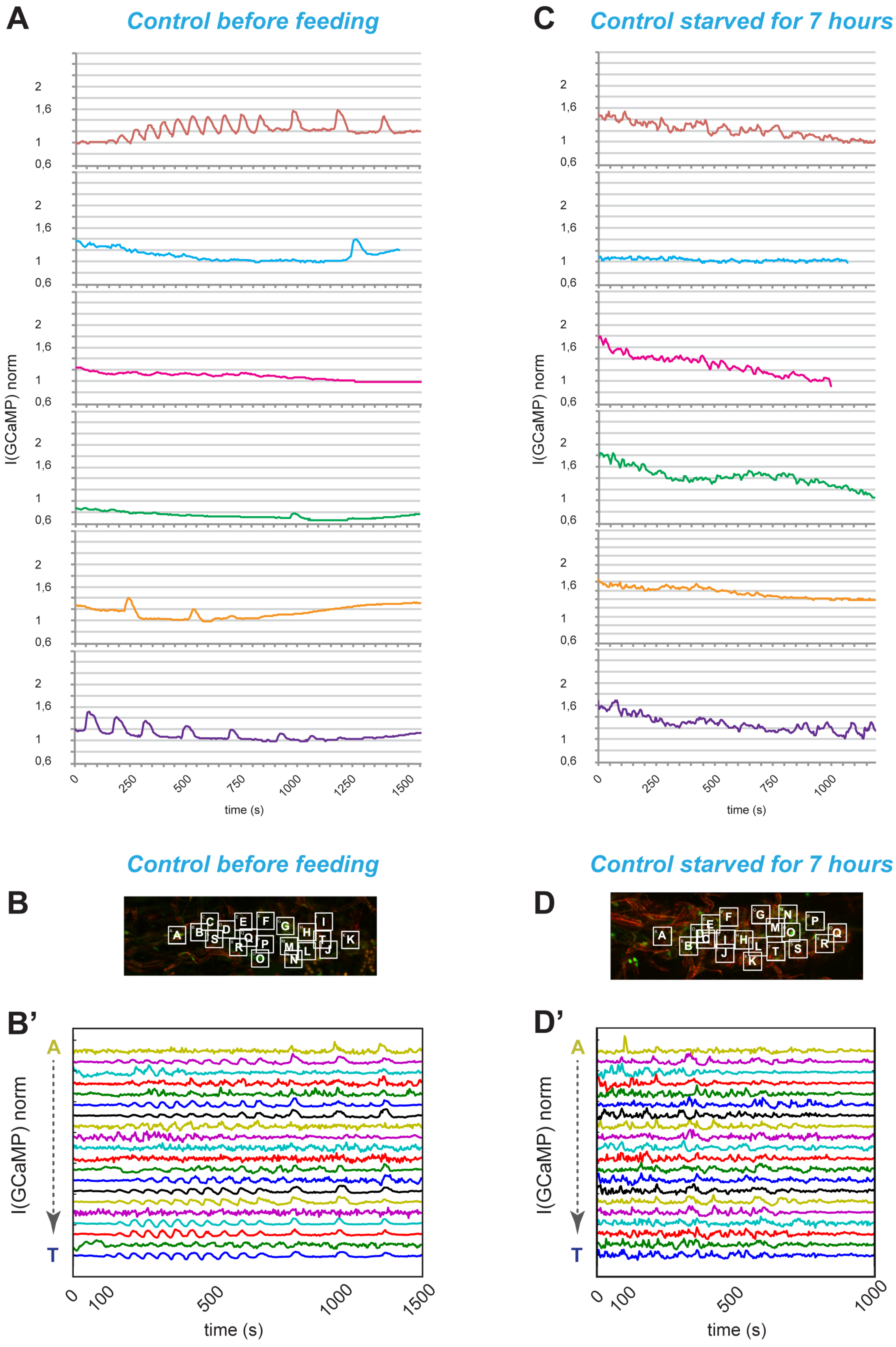
A
Control before feeding
C
Control starved for 7 hours
2
1,6
1
0,6
I(GCaMP) norm
0
250
500
750
1000
1250
1500
time (s)
B
Control before feeding
D
Control starved for 7 hours
B'
D'
A
T
I(GCaMP) norm
0
100
500
1000
1500
time (s)

Figure S6, related to Figure 6. Analysis of calcium oscillations in control conditions before feeding and after starvation. (**A**) Normalised GCaMP3 intensities plotted over time for 6 brains of control before feeding. **(B-B')** Correlation analysis for a newly hatched control larva (ALH0). (B) Map of the 20 ROIs at time 0. (B') Normalised calcium intensity over time for the 20 ROIs. **(C)** Normalised GCaMP3 intensities plotted over time for 6 brains of control starved for 7 hours. **(D-D')** Correlation analysis for a starved larva. (D) Map of the 20 ROIs at time 0. (D') Normalised calcium intensity over time for the 20 ROIs.

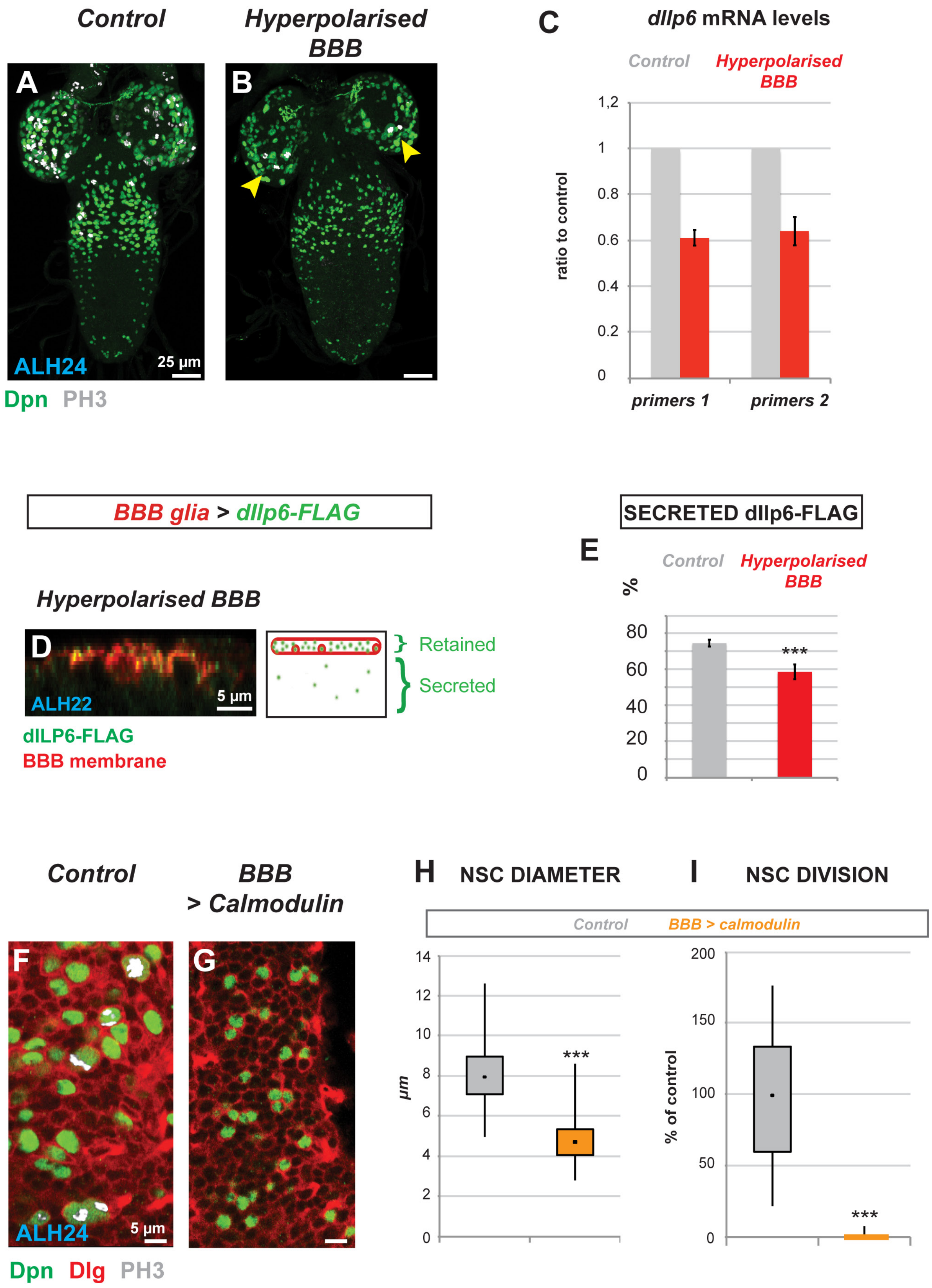

Control
Hyperpolarised BBB
A
B
ALH24
25 µm
Dpn PH3
C
dIlp6 mRNA levels
Control
Hyperpolarised BBB
ratio to control
1,2
1
0.8
0.6
0.4
0.2
0
primers 1
primers 2
BBB glia > dIlp6-FLAG
Hyperpolarised BBB
D
ALH22
5 µm
Retained
Secreted
dILP6-FLAG
BBB membrane
SECRETED dIlp6-FLAG
E
Control
Hyperpolarised BBB
%
80
60
40
20
0
***
Control
BBB > Calmodulin
F
G
ALH24
5 µm
Dpn Dlg PH3
H NSC DIAMETER
I NSC DIVISION
Control BBB > calmodulin
µm
14
12
10
8
6
4
2
0
***
% of control
200
150
100
50
0
***

Figure S7, related to Figure 7. Calcium signalling is required for NSC reactivation. (**A-B**) Nutrient-dependent NSC enlargement and division are blocked when the BBB glia is hyperpolarised, but the mushroom body neuroblasts, which do not enter quiescence, still divide (see arrowheads in B). NSC nuclei, green (Deadpan); Phospho-histone H3, grey. (**C**) Quantification of *dIlp6* transcript levels by q-pcr under BBB hyperpolarisation. Two pairs of *dIlp6* primers were used. Two biological replicates. See Extended Experimental procedures for details. ••• $p < 0.05$. Two-sided Student's test. Primers 1: $p=3.42 \times 10^{-4}$. Primers 2: $p = 4.40 \times 10^{-3}$. Bar graphs represent mean ± SEM. (**D-E**) Measure of dIlp6-FLAG secretion from the BBB glia upon BBB glia hyperpolarisation (*UAS-kir2.1-GFP; mdr65-GAL4, dIlp6-FLAG*), compared to control (*UAS-mCD8-GFP; mdr65-GAL4, dIlp6-FLAG*). (D) Cross section of one BBB glial cell and its schematic for hyperpolarisation condition. dIlp6-FLAG, green (FLAG); BBB membrane, red (RFP). (E) Measure of secreted dIlp6-FLAG (%). See Experimental procedures for details. ••• $p < 0.05$. Two-sided Student's test. Control n = 16 VNCs; hyperpolarised BBB n= 18 VNCs. $p = 1.73 \times 10^{-3}$ (unequal variance). Bar graphs represent mean ± SEM. (**F-I**) Calmodulin overexpression (*mdr65-GAL4, UAS-calmodulin*) blocks NSC reactivation (compare G to F). All images are anterior up, ventral view. NSC nuclei, green (Deadpan, Dpn); Cell cortices, red (Discs Large, Dlg); PH3, grey (phospho-histone H3). (H-I) Quantification of NSC (H) diameter and (I) proliferation; whisker plots. (H) ••• $p < 0.05$. Two-sided Student's test. Average and standard deviation were calculated from two biological replicates. Control n = 352 NSCs (22 VNC); *BBB glia > calmodulin* n = 328 NSCs (21 VNC); $p = 4.91 \times 10^{-163}$. (I) ••• $p < 0.05$. Two-sided Student's test. Average and standard deviation were calculated from two biological replicates. Control n = 22 VNCs; *BBB glia > calmodulin* n= 21 VNCs. $p = 2.19 \times 10^{-9}$ (unequal variance test).

Supplementary ables

**Gene**	**RNAi lines**	**Brain phenotype with *tub-GAL80^{ts}; insc-GAL4* (NSC knockdown)**	**Brain phenotype with *repo-GAL4* (glial knockdown)**
inx1 (ogre)	VDRC 7136 (GD library)	-	+++
	VDRC 103816 (KK library)	-	+++
	TRiP JF02595 (BL-27283)	-	++
inx2	***TRiP JF02446 (BL-29306)***	?	+++
inx3	VDRC 44767 (GD library)	-	-
	VDRC 39094(GD library)	-	-
	VDRC 39095(GD library)	-	-
	TRiP HM05245 (BL-30501)	-	-
inx4 (zpg)	VDRC 33277 (GD library)	?	-
inx5	VDRC 6950 (GD library)	-	-
	VDRC 6951 (GD library)	-	-
inx6	VDRC 8638 (GD library)	-	-
	VDRC 8639 (GD library)	-	-
	VDRC 46398 (GD library)	-	-
	VDRC 46399 (GD library)	-	-
inx7	VDRC 22948 (GD library)	-	-
	VDRC 22949 (GD library)	-	-
inx8 (ShakB)	VDRC 26801 (GD library)	-	-
	VDRC 26802 (GD library)	-	-

Table S1, related to Figure 1. *inxs* knockdown experiments. Neural defects were screened for overall brain volume and defects in an anti-Chaoptin staining. -, no obvious phenotype detected ; +, phenotype detected (the number of crosses represents the intensity of the phenotype); ?, small defects, not investigated further. The RNAi lines for *inx1* and *inx2* used in this study are shown as $inx1^{RNAi}$ and $inx2^{RNAi}$, in bold and italic.

	Shift 25 to 30°C		Shift 30 to 25°C	
ALH	% of brains with volume < 75% of control average	% of brains with volume > 75% of control average	% of brains with volume < 75% of control average	% of brains with volume > 75% of control average
-24	100	0	25	75
-18	100	0	25	75
-12	100	0	50	50
-6	100	0	75	25
0	100	0	100	0
24	100	0	100	0
48	0	100	100	0
72	0	100	100	0

No shift, 30°C from egg laying to analysis
No shift, 25°C from egg laying to analysis

Table S2, related to Figure 2. Temporal mapping of *inx1* function. For each temperature shift, brains with volume either under or over 75% of average volume of control were counted towards mutant or wild-type phenotype, respectively.

GAL4 driver	Reference	Brain expression	X *UAS-inx1*^{RNAi}, *UAS-inx2*^{RNAi}		X *UAS-inx1*^{DN}, *UAS-inx2*^{DN}	
			Brain phenotype	Other	Brain phenotype	Other
repo-GAL4	(Sepp et al., 2001)	all glia	+++	lethal 3rd instar	+++	
gcm-GAL4	(Chotard et al., 2005)	all glia	+++	lethal 3rd instar	+++	
moody-GAL4	(Schwabe et al., 2005)	subperineurial glia	++	phenotype at 29°C (elongated VNC)	+++	no phenotype per se at 25°C
moody-GAL4 ; UAS-GAL4	This study	subperineurial glia	++	phenotype at 29°C (elongated VNC)	ND	no phenotype per se at 25°C
NP2276	(Awasaki et al., 2008)	subperineurial glia	-		ND	
mdr65-GAL4	(Pfeiffer et al., 2008)	subperineurial glia	+++	phenotype at 29°C (elongated VNC)	+++	phenotype at 29°C (elongated VNC)
NP5606 (svp-GAL4)	(Awasaki et al., 2008)	subperineurial glia + neurons	++		+++	
Mz820-GAL4	(Ito et al., 1995)	channel glia	-		ND	
nrv2-GAL4	(Sun et al., 1999)	cortex glia	-		-	
NP2222-GAL4	(Awasaki et al., 2008)	cortex glia	-		-	
NP0577-GAL4	(Awasaki et al., 2008)	cortex glia	-		-	
moody-GAL4, nrv2-GAL4	This study	cortex + subperineurial glia	-		ND	
dILP6-GAL6	(Chell and Brand, 2010)	dILP6 glia	-		ND	
NP1079-GAL4	(Sousa-Nunes et al., 2011)	dILP6 glia	+		ND	
bnl-GAL4	(Kamimura et al., 2006)	glial subpopulation	-		ND	
serrate-GAL4	(Hukriede et al., 1997)	glial subpopulation	-		ND	
NP6293-GAL4	(Awasaki et al., 2008)	perineurial glia	-		ND	
c527-GAL4	(Hummel et al., 2002)	perineurial + subperineurial glia	-		ND	
NP3233-GAL4	(Awasaki et al., 2008)	astrocyte-like glia	-		ND	
NP1243-GAL4	(Awasaki et al., 2008)	astrocyte-like glia	-		ND	
alrm-GAL4	(Doherty et al., 2009)	astrocyte-like glia	-		ND	
NP6520-GAL4	(Awasaki et al., 2008)	ensheathing glia	-		ND	
Mz97-GAL4	(Hummel et al., 2002)	wrapping glia	-		ND	
c855a-GAL4	(Manseau et al., 1997)	neuroepithelium	-		ND	
NP6990-GAL4	(Awasaki et al., 2008)	neuroepithelium	-		ND	
NP0076	This study	*inx1* pattern	+		ND	
Mz1407 (inscuteable-GAL4)	(Betschinger et al., 2006)	NSC	-		ND	
grh-GAL4	(Chell and Brand, 2010)	NSC	-		ND	
prospero-GAL4	(Kambadur et al., 1998)	NSC + some GMC/neurons	-		ND	
grh-GAL4; repo-GAL4	This study	NSC + glia	+++	lethal 3rd instard	ND	
c155 (elav-GAL4)	(Lin and Goodman, 1994)	neurons	-		ND	
e22c-GAL4	(Brand and Perrimon, 1993)	epidermis	-	embryonic lethal	ND	
69B-GAL4	(Brand and Perrimon, 1993)	epidermis	-	semi-lethal	ND	
armadillo-GAL4	(Sanson et al., 1996)	ubiquitous	-	viable	ND	
tubulin-GAL4	(Lee and Luo, 1999)	ubiquitous	++	lethal 3rd instar	ND	

Table S3, related to Figure 3. List of all the GAL4 drivers used in this study, and associated phenotypes when driving different *inx* knockdown tools. ND, Not Determined. +++: 100% of the brains examined displayed a clear reduction in brain size. ++: at least 50% of the brains examined displayed a clear reduction in brain size. +: at least 25% of the brains examined displayed a clear reduction in brain size. -: no phenotype. Reductions in brain size were determined by the investigator after visual inspection under a confocal microscope.

Supplementary movie legends

Movie S1, related to Figure 5. The blood-brain barrier glia show spontaneous synchronised calcium oscillations. Live-imaging of the BBB of a young (ALH7) fed control larva. Calcium, green (GCaMP); membrane, red (mCD8-RFP). A duration of 1s in the movie corresponds to 70s during the experiment. The total length of the recording is approximately 15 min.

Movie S2, related to Figure 5. Coordinated calcium oscillations are lost when BBB gap junctions are disrupted. Live-imaging of the BBB of a young (ALH7) $inx1^{ogre\ jNL3}$ larva. Calcium, green (GCaMP); membrane, red (mCD8-RFP). A duration of 1s in the movie corresponds to 70s during the experiment. The total length of the recording is approximately 15 min.

Movie S3, related to Figure 6. Nutrition shapes and synchronises calcium oscillations in the blood-brain barrier glia. Live-imaging of the BBB of a just hatched (ALH0) control larva. Calcium, green (GCaMP); membrane, red (mCD8-RFP). A duration of 1s in the movie corresponds to 70s during the experiment. The total length of the recording is approximately 25 min.

Movie S4, related to Figure 6. Starvation prevents the synchronisation of calcium oscillations in the blood-brain barrier glia. Live-imaging of the BBB of a young (ALH7) starved control larva. Calcium, green (GCaMP); membrane, red (mCD8-RFP). A duration of 1s in the movie corresponds to 70s during the experiment. The total length of the recording is approximately 21 min.

Supplementary Experimental Procedures

Antibodies: Rabbit and guinea pig Inx1 antibodies were made using a C-terminal peptide (EFAKQVEPSKHDRAK), following the procedure described previously (Watanabe and Kankel, 1992).

DNA subcloning: UAS-Inx1 (full length amino acid (aa) 1-362), UAS-Inx1 Nter (aa 1-287), UAS-Inx1 Cter (aa 288-362), UAS-Inx1-GFP (aa 1-362) and UAS-GFP-Inx1 (aa 2-362) were cloned in the Gateway Carnegie collection, as were UAS-Inx2 (aa 1-367), UAS-Inx2 Nter (aa 1-287), UAS-Inx2 Cter (aa 288-367), UAS-Inx2-RFP (aa 1-367) and UAS-RFP-Inx2 (aa 2-367). Domains were determined according to previous studies (Bauer et al., 2005). For each construct, several independent transgenic lines were generated and tested. *UAS-dIlp6-FLAG* was generated by cloning the full length dIlp6 (aa 1-107) in frame with the pTWF vector of the Gateway Carnegie collection.

Larval culture: Larvae that hatched within a 60 min window (defined as after larval hatching 0h, or ALH0) were transferred to fresh yeast on standard fly food. Starvation was achieved by transferring larvae to a solution of 20% sucrose in PBS.

Immunohistochemistry: Larval brains were dissected according to standard procedures. Primary antibodies used were: Chicken anti-GFP (1/2000, 06-896, Upstate), rabbit anti-RFP (1/1000, ab62341, Abcam), rabbit or guinea pig anti-Inx1 (1/200, this study), rabbit anti-Inx2 (1/30, kind gift of M. Hoch and R. Bauer), mouse anti-Discs Large (1/100, 4F3 c, DSHB), Guinea Pig anti-Deadpan (1/5000, Brand lab), mouse anti-Repo (1/100, c 8D12, DSHB), rabbit anti-PH3 (1/100, 06-570, Millipore), mouse anti-FLAG (1/1000, M2, Sigma), mouse anti-Chaoptin (1/10, 24B10, DSHB). Samples were imaged on an Olympus Upright or Inverted FV1000 confocal microscope, or with a DeltaVision OMX microscope.

Image processing: Volocity or Fiji were used to process confocal data. Adobe Photoshop and Illustrator were used to assemble figures.

Quantitative Real-Time PCR: Total RNA was extracted from 60-75 VNCs per condition using TriZol reagent (Invitrogen). cDNA was prepared using the QuantiTect Reverse Transcription Kit (Qiagen). Q-pcr was performed on an Applied Biosystems StepOnePlus™ Real-Time PCR System machine), using SYBR green (Fast SYBR® Green Master Mix, Qiagen). Results were analysed using the Pfaffl method (Pfaffl, 2001), normalising against the geometric mean of three housekeeping genes: *GAPDH1*, *Act5C* and *rp49*. The primers were all used at 60°C annealing temperature, and their efficiencies were assayed on standard curves. Primers are listed below (F, forward primer; R, reverse primer):

Primers	Sequence 5’ to 3’	Amplicon (bp)	Efficiency (60°C)	Design
dILP6 Pair1 F	TGGCCCTTGGCGATGTATTTCC	84	1,87467	Quant Prime
dILP6 Pair1 R	ACTTGCAGCACAAATCGGTTACG			
dILP6 Pair2 F	CGATGTATTTCCCAACAGTTTCG	63	1,85241	(Grönke et al., 2010)
dILP6 Pair2 R	AAATCGGTTACGTTCTGCAAGTC			
GAPDH F	ATTTCGCTGAACGATAAGTTCGT	76	1,87403	(Chell and Brand, 2010)
GAPDH R	CGATGACGCGGTTGGAGTA			
Act5c F	AAGTTGCTGCTCTGGTTGTCG	280	1,83709	Quant Prime
Act5c R	GCCACACGCAGCTCATTGTAG			
rp49 F	CCAAGATCGTGAAGAAGCG	143	1,85149	Quant Prime
rp49 R	GTTGGGCATCAGATACTGTC			

Each condition was assayed with three or two biological replicates, each of them subjected to two independent reverse transcription reactions (RT). Each RT was tested in triplicate.

Dextran permeability: We followed previously published procedures (Hatan et al., 2011), with the following modifications: the permeant dye was 10-kDa Dextran (Texas Red, lysine fixable, D-1863, Invitrogen) at 50 mM for final concentration, and the bathing medium was HL-6. 10 larvae were analysed for each condition.

Brain explant culture: Brain explants of ALH0 larvae were cultured in Schneider’s medium (Gibco) supplemented with 10% Fetal Calf Serum (Sigma), 2 mM L-Glutamine (25030-032, Gibco) and Pen/Strept 1X (15070-063, Gibco) following published procedures (Britton and Edgar, 1998). The only modification was to use Nunclon 4-well round dishes (Sigma) and to fill the space between the wells with 1 ml H_2O_2 to enrich the atmosphere with oxygen. Freshly dissected third instar fat body was added to trigger NSC reactivation in the corresponding well. Carbenoxolone was added at a final concentration of 0.1 mM.

dIlp6 secretion experiments. For assessing dIlp6 secretion during BBB hyperpolarisation, the control condition was *tub-GAL80^{ts}, UASmCD8-GFP; mdr65-GAL4, dIlp6-FLAG* and the hyperpolarisation condition was *tub-GAL80^{ts}, UAS-kir2.1-GFP; mdr65-GAL4, dIlp6-FLAG*. We previously checked that kir2.1-GFP and mCD8-GFP were localising the same way in the BBB

membrane, by coexpressing each of them with mCD8-RFP. Retained dIlp6 intensity was measured as the dIlp6 signal colocalising with the BBB membrane (GFP signal). The measure was then conducted as described in Experimental procedures.

Calcium imaging: Fed/older larvae proved to be more sensitive to mechanical pressure and it was difficult to achieve a perfect and harmless immobilisation for a long period of time. This was the cause of the different recording lengths between control and *inx1* mutant at ALH7-10, versus control at ALH0 or after starvation. We decided to stop the movie at a maximum of 15 min for these conditions. Unfortunately, none of the chemical (CO_2, tricaïne, L-glutamate) or other mechanical immobilisation (slow melting agarose) methods tested seemed to work on young larvae.

Calcium tracking: As *moody-GAL4* also drives in the tracheal system, we were careful to exclude from our analysis calcium signals coming from this tissue.

Supplementary References

Awasaki, T., Lai, S.-L., Ito, K., and Lee, T. (2008). Organization and postembryonic development of glial cells in the adult central brain of Drosophila. J. Neurosci. *28*, 13742–13753.

Bauer, R., Löer, B., Ostrowski, K., Martini, J., Weimbs, A., Lechner, H., and Hoch, M. (2005). Intercellular communication: the Drosophila innexin multiprotein family of gap junction proteins. Chem. Biol. *12*, 515–526.

Betschinger, J., Mechtler, K., and Knoblich, J. a (2006). Asymmetric segregation of the tumor suppressor brat regulates self-renewal in Drosophila neural stem cells. Cell *124*, 1241–1253.

Brand, A.H., and Perrimon, N. (1993). Targeted gene expression as a means of altering cell fates and generating dominant phenotypes. Development *118*, 401–415.

Britton, J.S., and Edgar, B. a (1998). Environmental control of the cell cycle in Drosophila: nutrition activates mitotic and endoreplicative cells by distinct mechanisms. Development *125*, 2149–2158.

Chell, J.M., and Brand, A.H. (2010). Nutrition-responsive glia control exit of neural stem cells from quiescence. Cell *143*, 1161–1173.

Chotard, C., Leung, W., and Salecker, I. (2005). glial cells missing and gcm2 cell autonomously regulate both glial and neuronal development in the visual system of Drosophila. Neuron *48*, 237–251.

Doherty, J., Logan, M. a, Taşdemir, O.E., and Freeman, M.R. (2009). Ensheathing glia function as phagocytes in the adult Drosophila brain. J. Neurosci. *29*, 4768–4781.

Grönke, S., Clarke, D.-F., Broughton, S., Andrews, T.D., and Partridge, L. (2010). Molecular evolution and functional characterization of Drosophila insulin-like peptides. PLoS Genet. *6*, e1000857.

Hatan, M., Shinder, V., Israeli, D., Schnorrer, F., and Volk, T. (2011). The Drosophila blood brain barrier is maintained by GPCR-dependent dynamic actin structures. J. Cell Biol. *192*, 307–319.

Hukriede, N.A., Gu, Y., and Fleming, R.J. (1997). A dominant-negative form of Serrate acts as a general antagonist of Notch activation. Development *124*, 3427–3437.

Hummel, T., Attix, S., Gunning, D., and Zipursky, S.L. (2002). Temporal control of glial cell migration in the Drosophila eye requires gilgamesh, hedgehog, and eye specification genes. Neuron *33*, 193–203.

Ito, K., Urban, J., and Technau, G. (1995). Distribution, classification, and development ofDrosophila glial cells in the late embryonic and early larval ventral nerve cord. Dev. Genes Evol. *204*, 284–307.

Kambadur, R., Koizumi, K., Stivers, C., Nagle, J., Poole, S.J., and Odenwald, W.F. (1998). Regulation of POU genes by castor and hunchback establishes layered compartments in the Drosophila CNS. Genes Dev. *12*, 246–260.

Kamimura, K., Koyama, T., Habuchi, H., Ueda, R., Masu, M., Kimata, K., and Nakato, H. (2006). Specific and flexible roles of heparan sulfate modifications in Drosophila FGF signaling. J. Cell Biol. *174*, 773–778.

Lee, T., and Luo, L. (1999). Mosaic analysis with a repressible cell marker for studies of gene function in neuronal morphogenesis. Neuron *22*, 451–461.

Lin, D.M., and Goodman, C.S. (1994). Ectopic and increased expression of Fasciclin II alters motoneuron growth cone guidance. Neuron *13*, 507–523.

Manseau, L., Baradaran, A., Brower, D., Budhu, A., Elefant, F., Phan, H., Philp, A. V, Yang, M., Glover, D., Kaiser, K., et al. (1997). GAL4 enhancer traps expressed in the embryo, larval brain, imaginal discs, and ovary of Drosophila. Dev. Dyn. *209*, 310–322.

Pfaffl, M.W. (2001). A new mathematical model for relative quantification in real-time RT-PCR. Nucleic Acids Res. *29*, e45.

Pfeiffer, B.D., Jenett, A., Hammonds, A.S., Ngo, T.-T.B., Misra, S., Murphy, C., Scully, A., Carlson, J.W., Wan, K.H., Laverty, T.R., et al. (2008). Tools for neuroanatomy and neurogenetics in Drosophila. Proc. Natl. Acad. Sci. U. S. A. *105*, 9715–9720.

Sanson, B., White, P., and Vincent, J.P. (1996). Uncoupling cadherin-based adhesion from wingless signalling in Drosophila. Nature *383*, 627–630.

Schwabe, T., Bainton, R.J., Fetter, R.D., Heberlein, U., and Gaul, U. (2005). GPCR signaling is required for blood-brain barrier formation in drosophila. Cell *123*, 133–144.

Sepp, K.J., Schulte, J., and Auld, V.J. (2001). Peripheral glia direct axon guidance across the CNS/PNS transition zone. Dev. Biol. *238*, 47–63.

Sousa-Nunes, R., Yee, L.L., and Gould, A.P. (2011). Fat cells reactivate quiescent neuroblasts via TOR and glial insulin relays in Drosophila. Nature *471*, 508–512.

Sun, B., Xu, P., and Salvaterra, P.M. (1999). Dynamic visualization of nervous system in live Drosophila. Proc. Natl. Acad. Sci. U. S. A. *96*, 10438–10443.

Watanabe, T., and Kankel, D.R. (1992). The l(1)ogre gene of Drosophila melanogaster is expressed in postembryonic neuroblasts. Dev. Biol. *152*, 172–183.
