# Supplementary material for: Gap Junction Proteins in the Blood-Brain Barrier Control Nutrient-Dependent Reactivation of Drosophila Neural Stem Cells
Source: Dev Cell. 2014 Aug 11;30(3):309–21. doi: 10.1016/j.devcel.2014.05.021 (PMC4139190; doi:10.1016/j.devcel.2014.05.021)
Supplement: Document S2. Article plus Supplemental Information [file mmc6.pdf]

Developmental Cell
Article

CellPress

Gap Junction Proteins in the Blood-Brain Barrier Control Nutrient-Dependent Reactivation of *Drosophila* Neural Stem Cells

Pauline Spéder[1] and Andrea H. Brand[1,*]
[1]The Gurdon Institute and Department of Physiology, Development and Neuroscience, University of Cambridge, Tennis Court Road, Cambridge CB2 1QN, UK
*Correspondence: a.brand@gurdon.cam.ac.uk
http://dx.doi.org/10.1016/j.devcel.2014.05.021
This is an open access article under the CC BY license (http://creativecommons.org/licenses/by/3.0/).

SUMMARY

Neural stem cells in the adult brain exist primarily in a quiescent state but are reactivated in response to changing physiological conditions. How do stem cells sense and respond to metabolic changes? In the *Drosophila* CNS, quiescent neural stem cells are reactivated synchronously in response to a nutritional stimulus. Feeding triggers insulin production by blood-brain barrier glial cells, activating the insulin/insulin-like growth factor pathway in underlying neural stem cells and stimulating their growth and proliferation. Here we show that gap junctions in the blood-brain barrier glia mediate the influence of metabolic changes on stem cell behavior, enabling glia to respond to nutritional signals and reactivate quiescent stem cells. We propose that gap junctions in the blood-brain barrier are required to translate metabolic signals into synchronized calcium pulses and insulin secretion.

INTRODUCTION

Changes in environmental conditions can have a significant impact on the development and function of the brain. Neural stem cells (NSCs) integrate both local and systemic signals to modulate their rate and extent of proliferation to meet the needs of the organism (Kokovay et al., 2008). Most NSCs in the vertebrate adult brain exist in a mitotically dormant state. These quiescent NSCs are reactivated in response to a variety of metabolic stimuli (Rafalski and Brunet, 2011). Understanding how systemic and metabolic signals are sensed by the brain and converted into specific neural stem cell behaviors is essential to deciphering how the brain adapts to a changing environment.

In *Drosophila*, NSCs enter quiescence at the end of embryogenesis and are reactivated during early larval life in response to feeding (Britton and Edgar, 1998; Truman and Bate, 1988; Figure 1A). Amino acid availability is sensed by the fat body, the functional equivalent of the mammalian liver and adipose tissue (Andersen et al., 2013; Colombani et al., 2003). The fat body sends an as-yet-unidentified signal, or signals, to the brain to induce the production and secretion of insulin-like peptides (dIlps) by blood-brain barrier (BBB) glial cells. dIlps act locally to trigger the insulin/insulin-like growth factor receptor pathway in underlying NSCs (Chell and Brand, 2010; Sousa-Nunes et al., 2011). Consequently, the NSCs enlarge and re-enter the cell cycle.

NSC reactivation occurs synchronously in all neurogenic zones of the CNS, suggesting that BBB glial cells and/or NSCs are linked by an intercellular signaling mechanism. Gap junctions are intercellular channels formed by the juxtaposition of connexin hexamers (Segretain and Falk, 2004). They enable the propagation and amplification of signals within or between cell populations. Gap junctions are found throughout the mammalian brain and are important regulators of stem cell behavior, controlling self-renewal, survival, and aging (Kar et al., 2012; Wong et al., 2008). Here we show that gap junction proteins play a key role in the nutrient-dependent reactivation of dormant neural stem cells in the *Drosophila* brain. Interestingly, gap junction proteins are required in the BBB glia, but not in neural stem cells, for reactivation. We show that gap junction proteins coordinate nutrient-dependent calcium oscillations within the BBB and are required for the production and secretion of insulin-like peptides. Gap junction proteins thus enable the synchronous reactivation of quiescent stem cells throughout the CNS.

RESULTS

Inx1 and Inx2 Are Required for Normal Brain Development

To assess whether gap junctions play a role in NSC reactivation, we systematically targeted each of the eight members of the innexin (Inx) family (Bauer et al., 2005), the *Drosophila* functional equivalents of connexins and pannexins (Phelan, 2005; Figure S1A available online), by RNAi in either NSCs or glia (Table S1). Interestingly, we observed no detectable phenotype when innexins were knocked down in NSCs. However, knockdown of *innexin 1* (*inx1*) or *innexin 2* (*inx2*) in glia gave a striking phenotype in which brain size is dramatically reduced (Figures 1B–1D′) without affecting overall body size (data not shown). This suggests that the *inx* phenotype is not the result of a systemic growth defect but that *inx1* and *inx2* have a specific role in the brain. We checked the specificity of $inx1^{RNAi}$ and $inx2^{RNAi}$ using in silico methods (Naito et al., 2005), which predict no off-targets. Our data are consistent with the recent results of Holcroft et al., 2013, who showed that targeted RNAi against *inx1* (*ogre*) or *inx2*

CrossMark

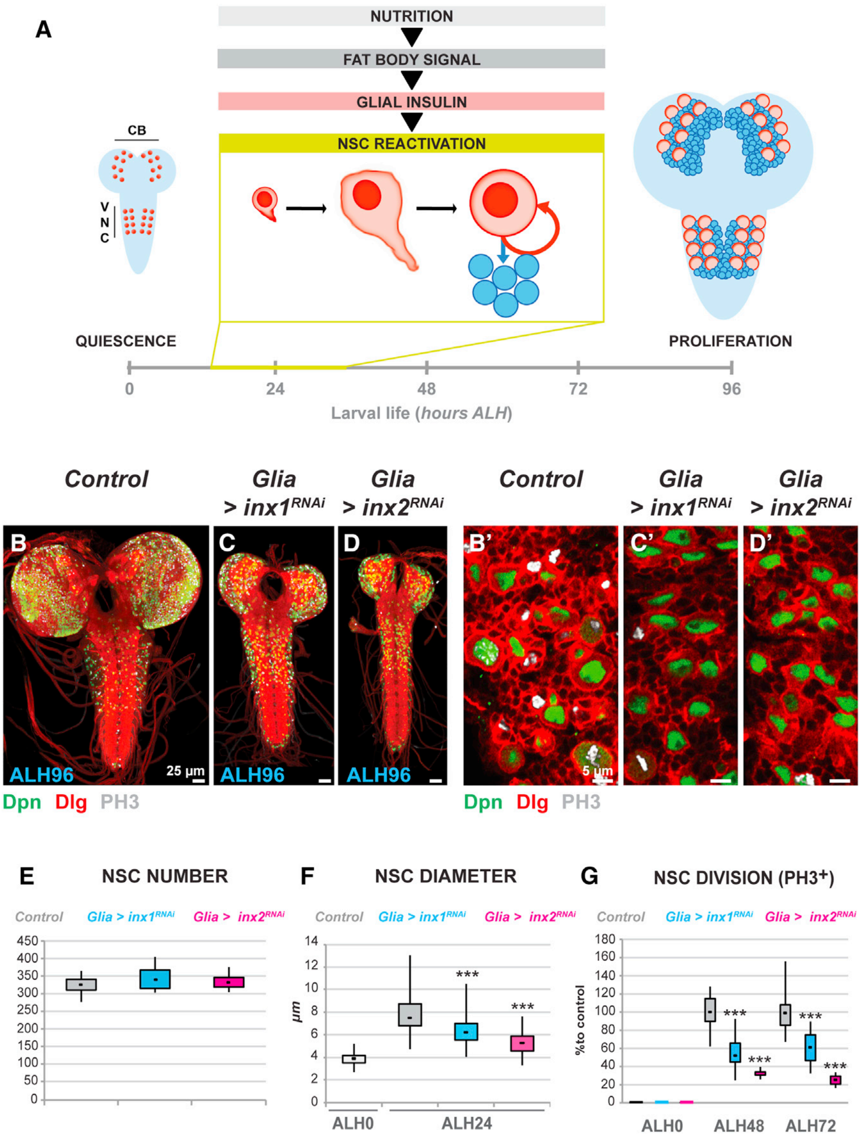

Figure 1. Glial Gap Junctions Are Required for Nutrient-Dependent Reactivation of NSCs

(A) *Drosophila* quiescent NSCs are reactivated within a 24 hr time window in response to nutrition. CB, central brain; VNC, ventral nerve cord; ALH, hours after larval hatching.

(B–D) Total brain size, NSC diameter and NSC proliferation are highly reduced after *inx1* or *inx2* RNAi in glia.

(B′–D′) Higher magnification ventral views of the VNCs in (B–D).

(E–G) Quantification of NSC (E) number, (F) diameter, and (G) proliferation in *inx1* or *inx2* knockdown in glia. (E) ***$p < 0.05$. Two-sided Student's t test. Average and SD were calculated from two biological replicates. Control n = 8 VNCs; *Glia > inx1*^{RNAi} n = 11 VNCs; *Glia > inx2*^{RNAi} n = 7 VNCs. *Glia > inx1*^{RNAi} p = 0.14. *Glia > inx2*^{RNAi} p = 0.53. (F) ***$p < 0.05$. Two-sided Student's t test. Average and SD were calculated from two biological replicates. Control ALH0 n = 224 NSCs (8 VNC); Control ALH24 n = 241 NSCs (15 VNCs); *Glia > inx1*^{RNAi} ALH24 n = 257 NSCs (11 VNCs); *Glia > inx2*^{RNAi} ALH24 n = 264 NSCs (11 VNCs). For ALH24: *Glia > inx1*^{RNAi} p = 1.23×10^{-28}; *Glia > inx2*^{RNAi} p = 2.47×10^{-78}. (G) ***$p < 0.05$. Two-sided Student's t test. Average and SD were calculated from two biological replicates. AH48. Control n = 17 VNCs; *Glia > inx1*^{RNAi} n = 18 VNCs; *Glia > inx2*^{RNAi} n = 8 VNCs. *Glia > inx1*^{RNAi} p = 3.52×10^{-7}. *Glia > inx2*^{RNAi} p = 2.00×10^{-8}. AH72. Control n = 14 VNCs; *Glia > inx1*^{RNAi} n = 13 VNCs; *Glia > inx2*^{RNAi} n = 4 VNCs. *Glia > inx1*^{RNAi} p = 1.11×10^{-4}. *Glia > inx2*^{RNAi} p = 1.40×10^{-5}. All images are anterior up, dorsal view unless stated otherwise. NSC nuclei, green (Deadpan, Dpn); Cell cortices, red (Discs Large, Dlg); PH3, gray (phospho-histone H3).

See also Figure S1 and Table S1.

in glia disrupts development of the larval nervous system and leads to adult behavioral phenotypes. We demonstrate that innexins are not required to link NSCs either to each other or to glial cells. Instead, Inx1 and Inx2 are required within the glial population alone for brain development.

Gap Junction Proteins Are Required in Glia for NSC Exit from Quiescence

To understand how glial gap junctions regulate growth in the CNS, we first examined NSC behavior after *inx1* or *inx2* knockdown at different time points during the process of NSC reactivation. Knockdown of *inx1* or *inx2* in glia did not affect the number of NSCs in the ventral nerve cord (VNC; Figure 1E), demonstrating that the phenotype is not due to the loss of NSCs prior to NSC reactivation (0 hr after larval hatching, ALH0). Next we assessed cell diameter because one of the earliest events in NSC exit from quiescence is cell enlargement (Chell and Brand, 2010; Sousa-Nunes et al., 2011). We found that NSC diameter is markedly reduced (ALH24) after *inx1* or *inx2* knockdown in glia (Figure 1F).

Finally, we assessed NSC proliferation after *inx* knockdown. We assayed the mitotic marker phosphohistone H3 (PH3) before NSC reactivation (ALH0), just after reactivation (ALH48) and at a time when wild-type NSCs are cycling actively (ALH72). Knockdown of either *inx1* or *inx2* in glial cells resulted in a severe reduction in the number of dividing NSCs at all times (Figure 1G). We found that NSC enlargement and entry into mitosis were also dramatically impaired in *inx1* and *inx2* mutants (*inx1*^{ogrejNL3} and *inx2*^{G0036}, respectively, see Figures S1B and S1C), and that reactivation could be rescued in *inx2* mutants by glial expression of *inx2* (Figures S1D–S1E′). We conclude that *inx1* and *inx2* are required in the glia for NSC exit from quiescence.

Inx1 and Inx2 Form Heteromeric Complexes

Gap junction proteins (connexins, pannexins, or innexins) are classically involved in forming intercellular channels or hemichannels, which enable exchange between the cytoplasm and the extracellular medium. Evidence also exists for channel-independent roles, such as cell adhesion and direct gene regulation (reviewed in Dbouk et al., 2009; Elias and Kriegstein, 2008). To test if channel function is important for NSC reactivation, we

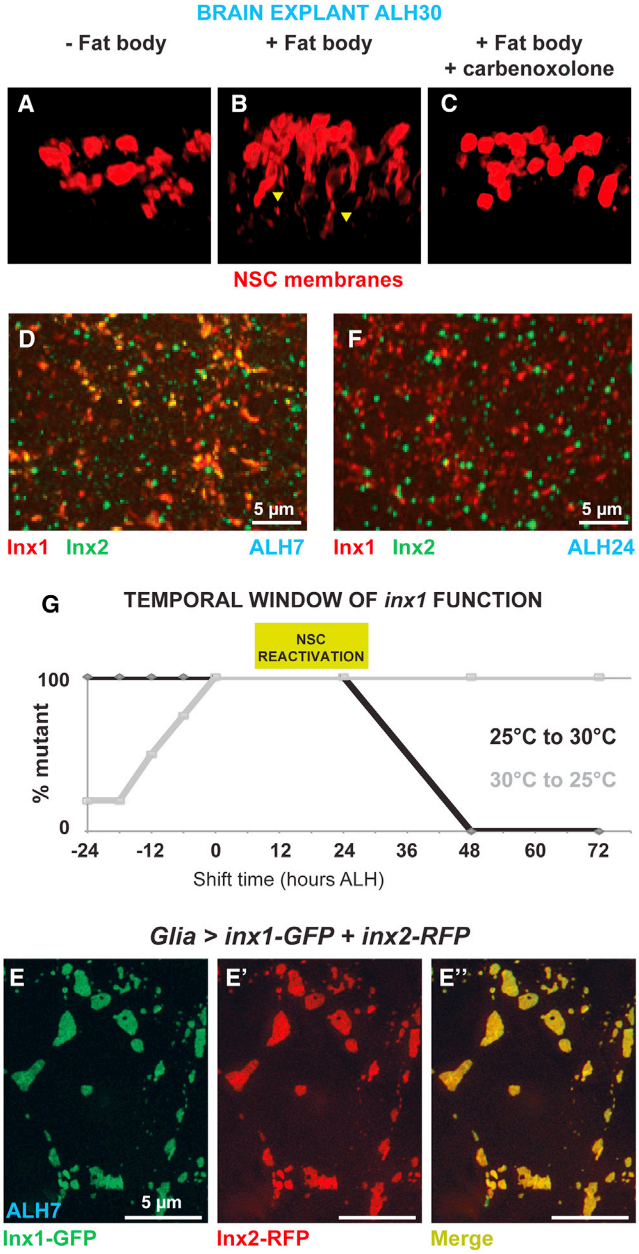

Figure 2. Inx1 and Inx2 Form Heteromeric and Temporally Regulated Channels in Glia

(A–C) Brain explant culture experiment. Brains from ALH0 larvae were cultured either on standard medium (A, – fat body), supplemented with fat body extract (B, + fat body) or supplemented with fat body extract and carbenoxolone (C, + fat body + carbenoxolone). Each image represents a three-dimensional reconstruction of a part of the VNC. Arrowheads indicate processes that NSCs extend during reactivation. See text and Supplemental Experimental Procedures for details of the protocol. NSC membrane, red (*grh-GAL4, UAS-myrRFP*).

(D) Confocal images showing colocalization of Inx1 (red) and Inx2 (green) in the VNC before NSCs enlarge. Extended projection.

(E–E″) Inx1-GFP (green) and Inx2-RFP (red), colocalize in plaques in glia (super resolution image).

(F) After NSCs exit quiescence Inx1 (red) and Inx2 (green) no longer colocalize.

(G) Temporal requirement of *inx1* activity. Temperature shifts of *tub-Gal80^{ts}, UAS-inx1^{RNAi}; repo-GAL4* flies, from the permissive (25°C) to the restrictive temperature (30°C; black) and vice versa (gray) identify the temperature sensitive period as between 0 and 24 hr ALH. The mutant phenotype was scored based on a reduction in brain volume. For each time point, 12 brains were analyzed. ALH, after larval hatching (at 29°C).

See also Figure S2 and Table S2.

treated brains in culture with carbenoxolone, a classic blocker of gap junction channels and hemi-channels (Giaume and Theis, 2010; see Supplemental Experimental Procedures). Carbenoxolone completely blocked NSC reactivation (Figures 2A–2C), implying a channel role for Inx1 and/or Inx2, the only innexins required for NSC reactivation. We also found that protein fusions that interfere with the folding of the innexin N-terminal domain (GFP-Inx1 and RFP-Inx2), which is essential for channel formation (Nakagawa et al., 2010), act as dominant-negative mutants (Figures S2A–S2C; data not shown for Inx2). This suggests that the function of Inx1 and Inx2 in glia is channel-based.

Inx1 and Inx2 could be part of the same channel or form two distinct channels, performing different functions that are both required for NSC reactivation. Gap junction channels are formed by the apposition of connexons (innexons in *Drosophila*) on adjacent cells. Connexons can be homomeric, formed from six molecules of a single subtype of connexin, or heteromeric, formed from different subtypes. In the larval VNC prior to NSC reactivation (ALH7), Inx1 and Inx2 were strongly expressed in glia and colocalized in plaques typical of gap junctions (Segretain and Falk, 2004; Figure 2D). Super-resolution microscopy (Dobbie et al., 2011) further demonstrated the tight association of Inx1 and Inx2, using tagged fusion proteins (Figures 2E–2E″). This close association is present from hatching and is not lost under starvation conditions (Figures S2D and S2E), demonstrating that formation of the complex is not driven by nutrition.

We found that most Inx1 staining was lost after knockdown of *inx2* in glia, and vice versa. This suggests that Inx1 and Inx2 localization is interdependent (Figures S2F–S2H) and that Inx1 and Inx2 form heteromeric innexons (Segretain and Falk, 2004) rather than independent gap junction channels (Lehmann et al., 2006). Inx1 and Inx2 have been shown to form functional heteromeric channels in paired *Xenopus* oocytes (Holcroft et al., 2013). We conclude, therefore, that Inx1 and Inx2 form heteromeric channels or hemi-channels in the glia.

Inx1 and Inx2 Channels Are Developmentally Regulated

Interestingly, we observed a change in Inx1/Inx2 colocalization over time. By ALH24, when reactivation has taken place, Inx1 and Inx2 are still expressed but they no longer colocalize (Figure 2F), suggesting that formation of Inx1/Inx2 channels is temporally regulated. Consistent with this observation, we discovered that the temporal requirement for *inx1* and *inx2* function in NSC reactivation is between ALH0 and ALH24 (Figure 2G, Table S2, and data not shown). Therefore, the formation and maintenance of Inx1 and Inx2 heteromeric channels are developmentally regulated and coincide with the time when the innexins are required for NSC reactivation.

Inx1/Inx2 Gap Junctions Are Required in the BBB Glia for NSC Reactivation

Inx1/Inx2 channels are required in glia to transmit nutritional stimuli to quiescent NSCs, They are likely to be found, therefore,

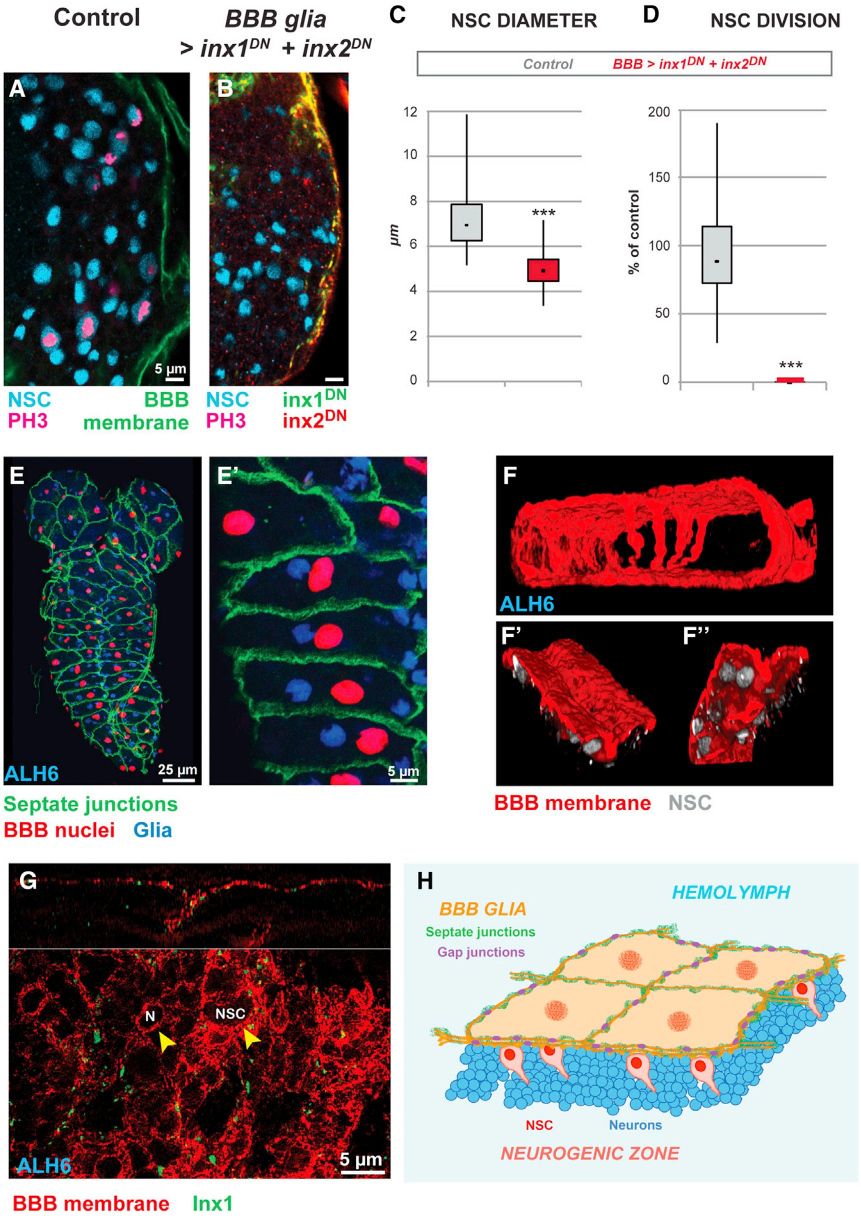

Figure 3. Inx1 and Inx2 Are Required in the BBB Glia for NSC Reactivation

(A–D) Blocking *inx1* and *inx2* function in BBB glia phenocopies pan-glial knockdown. Confocal images of VNC NSCs in (A) control brain (*mdr65-GAL4, UAS-mCD8-GFP*) and (B) brain where dominant-negative forms of Inx1 and Inx2 have been driven in the subperineurial glia only (*mdr65-GAL4* driving $inx1^{DN}$ and $inx2^{DN}$). NSC, cyan (Dpn) ; Phospho-histone H3, magenta; BBB membrane, green (GFP) or $inx1^{DN}$ (GFP-Inx1), green and $inx2^{DN}$ (RFP-Inx2), red. Quantification of NSC (C) diameter and (D) proliferation. (C) ***$p < 0.05$. Two-sided Student's t test. Average and standard deviation were calculated from two biological replicates. Control n = 148 NSCs (11 VNC); *BBB Glia* > $inx1^{DN}$ + $inx2^{DN}$ n = 143 NSCs (10 VNCs). $p = 4.19 \times 10^{-53}$. (D) ***$p < 0.05$. Two-sided Student's t test. Average and standard deviation were calculated from two biological replicates. Control n = 11 VNCs; *BBB Glia* > $inx1^{DN}$ + $inx2^{DN}$ n = 10 VNCs. $p = 4.44 \times 10^{-4}$.

(E and E′) Organization of early BBB glial cells. (E′) Close up of (E). Septate junctions, green (Lachesin-GFP) ; BBB nuclei, red (*moody-GAL4, UAS-Histone-RFP*); glial nuclei, blue (Repo). Anterior up, ventral view.

(F–F′′) Three-dimensional reconstruction of a part of the BBB membrane, red (*moody-GAL4, UAS-mCD8-RFP*). Ventral up, dorsal down. (F′ and F′′) Apical and basal views of ventral BBB membrane, respectively. NSC, gray (Dpn).

(G) Super resolution image of BBB glial membrane. Inx1, green; membrane, red (*Moody-GAL4, UAS-mCD8-RFP*). Top: orthogonal section; bottom panel, single focal plane of the ventral BBB glia. The "holes" (see arrowheads) in the BBB membrane are created by the NSCs or neurons (N) upon which the BBB glia rest like a sheet, resulting in small invaginations.

(H) Schematics of the BBB.

See also Figure S3 and Table S3.

in cells situated between the NSCs and the exterior of the brain. To determine in which glial cells Inx1/Inx2 are required, we knocked down Inx1/Inx2 in different glial populations using subtype-restricted GAL4 drivers to drive RNAi or express dominant-negative constructs (Table S3). We found that *inx* function is necessary within the subperineurial glia because knockdown in this glial subtype alone phenocopies knockdown in the entire glial population (Table S3 and Figures S3A and S3B), preventing NSC reactivation (Figures 3A–3D).

The subperineurial glia and the perineurial glia constitute the *Drosophila* BBB (Stork et al., 2008). In vertebrates, the BBB consists of a single layer of vascular endothelium closely associated with astrocytic glia (Daneman, 2012). The BBB shields the brain from the external environment owing to tight junctions between endothelial cells. It acts as a selective sieve to reject potentially neurotoxic factors but allow the passage of nutrients, ions, or other signals to maintain brain homeostasis (Daneman, 2012).

The *Drosophila* BBB exhibits similar neuroprotective strategies to its vertebrate counterpart, including a layer that limits the diffusion of neurotoxic factors, and an array of conserved transporters that regulates BBB permeability (Daneman and Barres, 2005; DeSalvo et al., 2011; Mayer et al., 2009). The subperineurial glia are large, flat polyploid (Unhavaithaya and Orr-Weaver, 2012) cells (Figures 3E–3E′) that envelop the brain (Figure 3F) and are closely apposed to the NSCs (Figures 3F′ and 3F′′). They isolate the brain from the hemolymph (the *Drosophila* equivalent of blood) by virtue of lateral septate junctions (Figures 3E and 3E′; Schwabe et al., 2005; Stork et al., 2008).

Knockdown of *inx* did not disrupt the septate junctions (Figures S3C and S3D), and we were not able to see a change in Dextran dye penetration (data not shown; Pinsonneault et al., 2011; Schwabe et al., 2005). Although weak permeability defects are difficult to detect at this stage and cannot be excluded, they do not prevent NSC reactivation ($moody^{\Delta 17}$

mutant (Bainton et al., 2005), data not shown). These data suggest that the *inx* mutant phenotype is not due to an impaired, leaky BBB. Using super-resolution microscopy, we detected Inx1 (and Inx2, data not shown) along the BBB membranes and the septate junctions lining the lateral cell membranes (Figures 3G and S3E). We conclude that Inx1/Inx2 channels are required autonomously in the BBB glial cells for NSC reactivation (Figure 3H).

Inx1/Inx2 Gap Junctions Are Required for Insulin Signaling

NSC reactivation requires the expression and secretion of insulin-like peptides, dIlps, by BBB glial cells (Chell and Brand, 2010). Of the eight identified insulin-like peptides in *Drosophila*, *dIlp6* transcription was shown to increase dramatically in the CNS upon feeding. Furthermore, when larvae were starved, forced expression of *dIlp6* in the glia was able to rescue NSC reactivation (Chell and Brand, 2010). dIlp6 binds to the insulin receptor (InR) on NSCs, activating the PI3K/Akt pathway and inducing exit from quiescence (Chell and Brand, 2010).

We assayed whether gap junction proteins within the BBB are required for insulin signaling. We found a significant decrease in *dIlp6* transcription after knocking down both *inx1* and *inx2* in the glia (Figure S4A). We next assayed dIlp6 secretion. In the absence of an effective dIlp6 antiserum, we expressed a tagged, functional version of dIlp6 (dIlp6-FLAG) in the BBB glia. We found that dIlp6 secretion from the BBB glia was strongly impaired in *inx1* loss-of-function mutants (Figures 4A, 4B, and 4D). Secretion of dIlp6 was similarly impaired upon starvation (Figures 4A, 4C, and 4E). Therefore, both the expression and secretion of dIlp6 are regulated by nutrition and depend on gap junction proteins.

NSC reactivation in *inx* mutants was rescued by forced expression of *dIlp6* in glia (Figures 4F–4H′, see arrowheads), as shown by the recovery of brain volume (80% of the brains, Figure 4I) and of NSC diameter (Figure 4J). Direct activation of the PI3K/Akt pathway in NSCs also resulted in rescue of brain volume and NSC enlargement and entry into mitosis (Figures S4B–S4C′). We conclude that gap junction proteins in the BBB glia are required to activate insulin signaling and induce NSC reactivation.

Gap Junctions Coordinate Calcium Oscillations in the BBB Glia

Secretion of insulin by the pancreas is induced by glucose, leading to synchronized calcium oscillations within gap junction-coupled beta cells and insulin exocytosis (MacDonald and Rorsman, 2006). Gap junctions enable the passage of secondary messengers that either trigger the release of calcium from intracellular stores or the influx of calcium from the extracellular environment (Laude and Simpson, 2009; Leybaert and Sanderson, 2012; Orellana et al., 2012; Skupin and Thurley, 2012). Blocking gap junctions inhibits coordinated intercellular calcium signaling (Leybaert and Sanderson, 2012; Orellana et al., 2012). Gap junction proteins are thus an important means of transmitting calcium waves.

To investigate whether calcium signaling plays a role in gap junction-mediated NSC reactivation, we expressed a calcium sensor, GCaMP3 (Tian et al., 2009), in the BBB glia (Figure 5A; Figures S5A–S5C) of living larvae. Before reactivation (ALH7) the BBB glia of feeding larvae exhibited clear calcium oscillations (Movie S1; Figure 5B). The BBB glia pulsed simultaneously, suggesting that calcium oscillations are coordinated across the entire CNS. Individual cell tracking showed that glial calcium oscillations exhibited striking synchrony (Figures 5B′ and 5B″) in all brains analyzed (n = 15, six are displayed; Figure S5D).

To further assess the extent of calcium oscillation coordination within the BBB glia under these conditions, we performed correlation analysis (see Experimental Procedures) for 20 regions of interest (ROI) chosen at random within the BBB glial layer (Figures 5C, S5E, and S5E′). In fed larvae before reactivation, the central correlation peak (coefficient 1) demonstrates the synchronicity of calcium oscillations within the BBB layer. Additional peaks on each side reveal that this synchronicity repeats (see Experimental Procedures for details).

Next, we monitored calcium dynamics in *inx1* mutants. None of the mutant brains (n = 11) showed coordinated calcium oscillations (Movie S2; Figure 5D; Figure S5F). Instead, BBB glial cells pulse independently, with no coordination between neighboring cells (Figures 5D′ and 5D″). We conclude that Inx1/Inx2 gap junctions are required to coordinate synchronous calcium oscillations within the BBB glia. In accordance with our observation, the graph of correlation coefficient for *inx1* mutants established the total absence of synchronicity between BBB glial cells (Figures 5E, S5G, and S5G′), showing that gap junctions are required for propagating calcium oscillations within the BBB.

Calcium Oscillations in the BBB Glia Respond to Nutritional Status

To assess whether calcium oscillations in the BBB are induced by a nutritional stimulus, we first assayed calcium dynamics in the BBB glia of newly hatched larvae (ALH0), before they started to feed. The calcium oscillations differed both in extent and frequency from those seen in fed larvae (n = 9; Movie S3; Figures 6A–6A″; Figure S6A). Correlation analysis revealed a partial coordination within the BBB glia (central correlation peak with a coefficient of 0.5, Figures 6B, S6B, and S6B′), strengthening the idea that nutrition is important for extending and establishing robust calcium synchronicity in the BBB.

Next, we assessed calcium dynamics after starvation, specifically the absence of essential amino acids. Calcium dynamics in the BBB of starved larvae resembled *inx1* mutant brains (Movie S4; Figure 6C). Synchronous calcium oscillations were completely abolished in all brains examined (n = 19, Figure S6C). Individual BBB cells displayed some calcium pulses (Figures 6C′ and 6C″), but with different profiles to those seen in fed larvae. In addition, correlation analysis of starved larvae showed very weak synchronicity (Figures 6D, S6D, and S6D′). This suggests that the synchronous, nutrition-dependent, calcium oscillations are lost upon starvation. Importantly, neither Inx1 nor Inx2 is lost under starvation conditions (Figure S2E). We conclude that nutrition, in particular essential amino acids, shape calcium dynamics. Upon feeding, calcium oscillations are amplified and synchronized across the BBB.

The IP3 Pathway and BBB Membrane Polarization Control Neural Stem Cell Reactivation

We assayed whether glial calcium oscillations arise from the release of intracellular calcium, or from the influx of extracellular

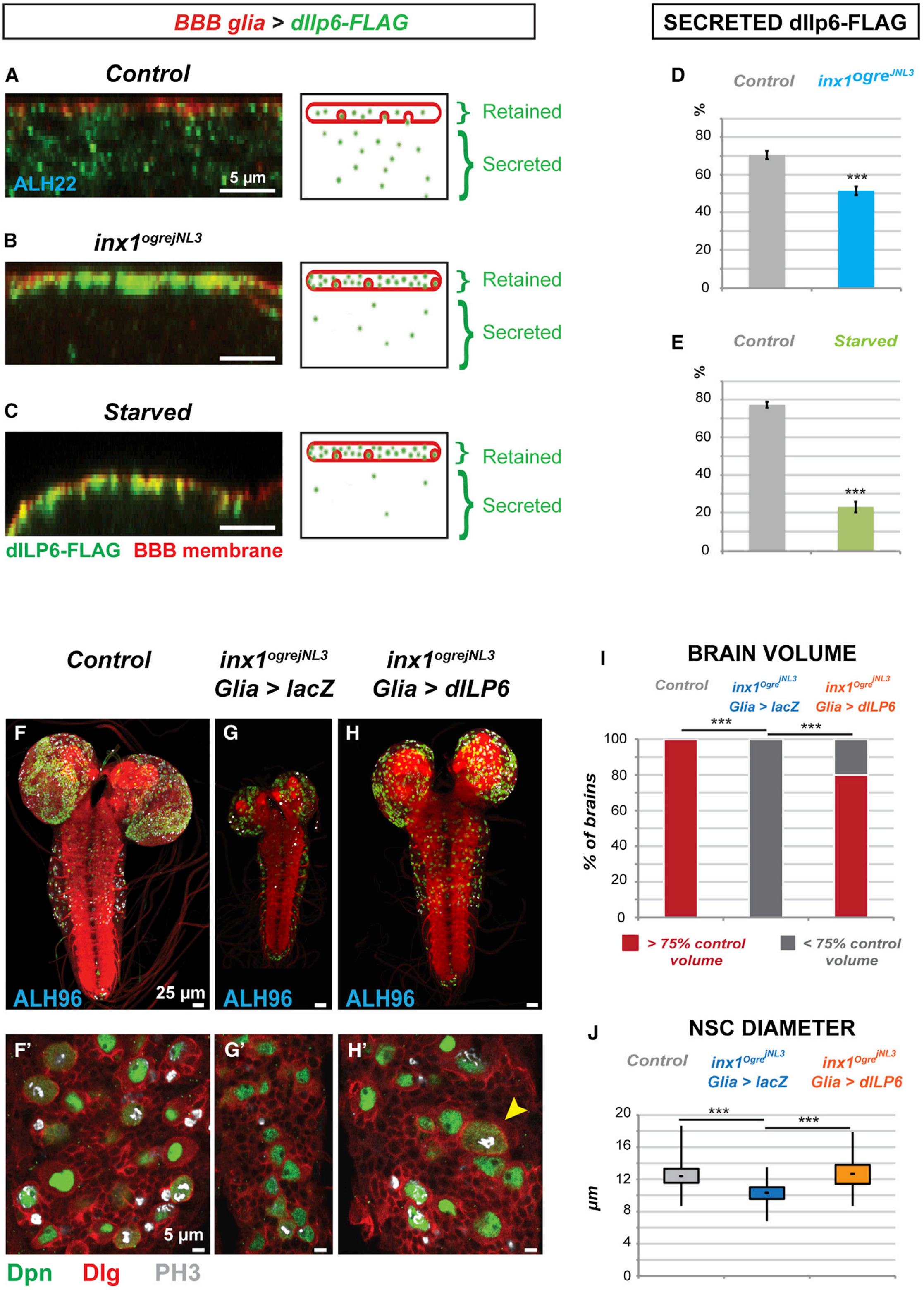

Figure 4. Gap Junctions Control NSC Reactivation through the Insulin Pathway

(A–D) dIlp6 secretion depends on gap junction and nutrition. dIlp6 secretion was assayed by expressing a tagged version in the BBB glia only (*moody-GAL4, UAS-mCD8-RFP, UAS-dIlp6-FLAG,*) and assessing what is found out of the BBB glia. Cross-section of one BBB glial cell and its schematic for (A) control, (B) $inx1^{ogrejNL3}$ mutant, and (C) starved. dIlp6-FLAG, green (FLAG); BBB membrane, red (RFP). (D and E) Secreted dIlp6-FLAG (%) was measured as the ratio

(legend continued on next page)

calcium, and how these influence NSC reactivation. First, we assessed the importance of the inositol-triphosphate (IP3) pathway. The IP3 pathway triggers calcium release from intracellular stores (Berridge, 2009). Stimulation of G-coupled receptors by a wide range of signals activates phospholipase C, leading to the production of IP3 from cleaved PIP2. IP3 then binds to its receptor (Ins3PR), a ligand-gated Ca^{2+} channel found on the surface of the ER, releasing intracellular calcium. We knocked down Ins3PR (*itpr* in *Drosophila*) in the BBB glia by RNAi (Chorna and Hasan, 2012). Both NSC enlargement and proliferation were strongly impaired (Figures 7A–7D). We conclude that NSC reactivation depends on IP3-mediated release of calcium from intracellular stores.

Next, we assessed the importance of calcium influx. Membrane depolarization triggers the entry of extracellular calcium via voltage-gated calcium channels (Catterall, 2011; Leybaert and Sanderson, 2012), whereas hyperpolarization prevents it. We hyperpolarized BBB membranes by expressing the inward-rectifying potassium channel, kir2.1 (Baines et al., 2001). BBB hyperpolarization blocks NSC reactivation dramatically, as revealed by the complete failure of both enlargement and mitotic re-entry (Figures 7E–7H). Interestingly, the mushroom body neuroblasts, a small group of central brain NSCs that do not undergo quiescence and reactivation, are not affected by BBB hyperpolarization, suggesting that nutrition-dependent NSC reactivation is specifically affected (Figures S7A and S7B, see arrowheads). BBB hyperpolarization both decreases *dIlp6* mRNA levels (Figure S7C) and dIlp6 secretion (Figures S7D and S7E), similar to what is seen during starvation or gap junction loss-of-function (see Figure S4A and Figures 4A–4E). In support of the role of calcium oscillations in reactivating NSCs, we found that overexpressing the calcium-binding protein, calmodulin, prevents NSC reactivation (Figures S7C–S7F). Our results show that intracellular and extracellular calcium both contribute to NSC reactivation.

DISCUSSION

Gap Junctions Are Required in the BBB for NSC Reactivation

The nutrient-dependent reactivation of NSCs in the *Drosophila* brain demonstrates how NSCs can adapt to environmental changes to fulfil the needs of the organism. Here, we show that gap junction proteins within the BBB glia are required for insulin expression and secretion, a prerequisite for NSC exit from quiescence (Figure 7I). We demonstrate that gap junction proteins coordinate glial calcium oscillations that are required for NSC reactivation. Both intracellular calcium stores and calcium influx contribute to reactivation. Membrane depolarization is known to regulate exocytosis via calcium signaling (Fridlyand and Philipson, 2011; Stojilkovic, 2012; Südhof, 2012), which controls stimulus-secretion coupling in secretory cells, such as in endocrine cells (Dolenšek et al., 2011; Géminard et al., 2009; Stojilkovic, 2012; Südhof, 2012). We show that conditions that block calcium oscillation in the BBB glia (the loss of gap junction proteins, starvation) also impair insulin secretion.

Mechanisms of Insulin Production and Secretion in *Drosophila* and Vertebrates

The sequence of events leading to glial secretion of insulin bears a striking resemblance to the diet-induced release of insulin by the beta cells of the pancreas (MacDonald and Rorsman, 2006). In the pancreas, a nutritional stimulus is sensed by gap junction-coupled beta cells, inducing depolarization resulting in synchronized calcium oscillation and insulin secretion. Loss of gap junction coupling results in uncoordinated calcium pulses. In *Drosophila inx* loss of function mutants, individual subperineurial BBB glial cells oscillate independently of one another. Compared to starvation, in which the nutritional signal is absent and NSC reactivation cannot occur, the scattered signals from individual BBB cells are able to induce delayed, asynchronous, reactivation in a small number of NSCs (see PH3 positive NSCs in Figures 1C′–1D′). We propose that gap junction function within the BBB enables glial insulin release to reach a threshold high enough to trigger NSC reactivation throughout the central nervous system.

In both beta cells and BBB cells, membrane depolarization is crucial for generating calcium oscillations. Failure to depolarize or an active block to depolarization prevents insulin release. However, sustained depolarization of β cells can lead to desensitization and a decline in insulin release (Willenborg et al., 2012). Interestingly, we find that forced depolarization of BBB glia only mildly enhances NSC reactivation (data not shown). This could be due to desensitization or it may be that the system is already maximally active.

Insulin mRNA levels are decreased after gap junction knockdown, both in pancreatic islets (Bosco et al., 2011) and in the BBB glia. In both cases it remains to be determined if calcium oscillations can directly affect gene expression, as has been shown in other systems (Alonso and García-Sancho, 2011).

Insulin produced by the pancreas is distributed via the circulatory system, whereas glial insulin is secreted locally, directly to underlying NSCs. Glial insulin signaling is thus contained within the brain, enabling local, differential regulation of this organ. The BBB acts both as a niche and as a protective barrier, providing specific factors directly to the stem cell while shielding the brain from unwelcome systemic regulation. In the context of NSC reactivation, these two roles are conveniently

between secreted dIlp6-FLAG and total dIlp6-FLAG. See Experimental Procedures for details. ***$p < 0.05$. Two-sided Student's test. (D) Control n = 38 VNCs; $inx1^{ogrejNL3}$ n = 35 VNCs. $p = 7.21 \times 10^{-8}$. (E) Fed n = 19 VNCs; starved n = 18 VNCs. $p = 4.56 \times 10^{-18}$. Bar graphs represent mean ± SEM.

(F–J) Rescue of NSC reactivation in *inx1* mutants by glial expression of *dIlp6*. Anterior up, dorsal view. (F′–H′) Higher magnification. Ventral views. NSC nuclei, green (Deadpan); Cell cortices, red (Discs Large); Phosphohistone H3, gray. (I) Quantification of brain volume rescue. A one-way Anova test was performed. ***$p < 0.05$. Control n = 10 brains; $inx1^{ogrejNL3}$; *glia > lacZ* n = 12 brains; $inx1^{ogrejNL3}$; *glia > dIlp6* n = 10 brains. For control versus mutant, $p = 8.68 \times 10^{-12}$. For mutant versus rescue, $p = 2.01 \times 10^{-2}$. For control versus rescue, p = 0.12. (J) Quantification of NSC diameter rescue. A one-way Anova test was performed. ***$p < 0.05$. Control n = 182 NSCs (10 VNCs); $inx1^{ogrejNL3}$; *glia > lacZ*; n = 198 NSCs (12 VNCs); $inx1^{ogrejNL3}$; *glia > dIlp6*; n = 219 NSCs (10 VNCs). For control versus mutant, $p = 2.30 \times 10^{-42}$. For mutant versus rescue, $p = 1.84 \times 10^{-42}$. For control versus rescue, p = 0.21.
See also Figure S4.

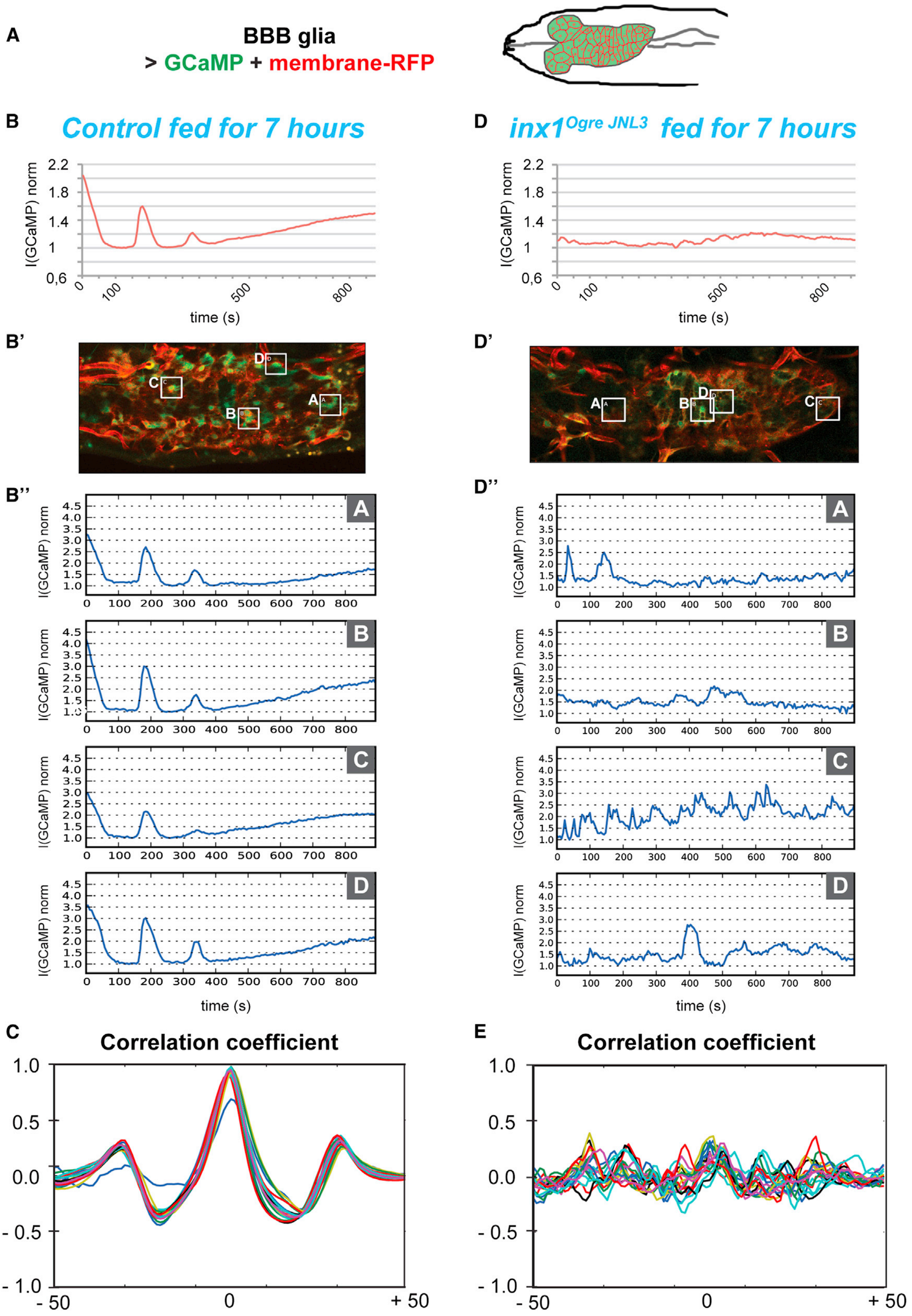

(legend on next page)

complementary. In the vertebrate BBB, similar functions may be split between endothelial cells and astrocytic glia. The vascular endothelium provides the barrier function, while astrocytic glia have a regulatory role in sensing and adjusting barrier permeability to various stimuli (Daneman, 2012). BBB endothelial cells can secrete cytokines, chemokines, and prostaglandins, suggesting that the BBB behaves like an endocrine tissue (Banks, 2012). Interestingly, calcium oscillations have been observed in cultured vertebrate BBB endothelial cells, but their function is largely unknown (De Bock et al., 2013).

Gap Junction Function in Neural Stem Cells

Gap junction communication can influence stem cell behavior by directly coupling stem cells to each other or to supporting cells, such as found in a stem cell niche. In the brain, connexon-mediated communication has been reported to occur between progenitor cells, within astrocytic networks, and between radial glia and neurons or progenitor cells and astrocytes (Giaume et al., 2010; Lacar et al., 2011; Nakase and Naus, 2004; Lo Turco and Kriegstein, 1991). The proliferation of neural progenitors and the formation of cortical layers in the mouse brain depend on an intercellular gap junction network (Malmersjö et al., 2013), and grafted human NSCs integrate into organotypic cultures through connexin coupling (Jäderstad et al., 2010). Here, we show that gap junction function within a niche, the BBB, can also influence NSC behavior.

The *Drosophila* BBB is a protective and selective barrier as well as a signaling center that orchestrates major developmental and physiological events. Here we show that gap junction communication enables cells within the BBB to act as a concerted unit, leading to coordinated calcium signaling and insulin release. Similarities between the BBB in vertebrates and invertebrates suggest that our findings are likely to have broader significance.

EXPERIMENTAL PROCEDURES

Genetics

The RNAi and GAL4 drivers used in this study are listed in Tables S1 and S3. *inx1*^{ogrejNL3} and *moody-GAL4* were kind gifts of P. Phelan and R. Bainton, respectively. All RNAi experiments were conducted at 29°C. All glial knockdowns were performed with *repo-GAL4* as driver, unless stated otherwise. The BBB driver was either *moody-GAL4* or *mdr65-GAL4* (see for each experiment).

Statistics

Bar graphs were generated using the mean and SEM for each sample. Error bars represent SEM. Center values are averages. Student's t test was performed using a threshold of $p < 0.05$ (confidence interval of 95%), represented by ***. All tests performed in this study were two-sided and with samples of same variance, unless stated otherwise.

For rescue of *inx1* mutant by glial expression of *dIlp6*, we used a one-way Anova test, with $p < 0.05$ represented by ***. Whisker plots were drawn using the minimum, quartile 1, median, quartile 3, and maximum of each condition's sample. In addition, a Student's t test based on a sample's average and SD was used to generate p values with the same criteria as above. *n* refers to the total number of samples for all biological or technical replicates. NSC numbers were determined using measurement scripts in Volocity software (PerkinElmer).

Temperature Shift Experiments

We used *tub-Gal80*^{ts}*, inx1*^{RNAi} *; repo-GAL4* to achieve conditional expression of *inx1*. At 25°C (permissive temperature for *Gal80*^{ts}), larvae showed a wild-type phenotype, but at 30°C (restrictive temperature for *Gal80*^{ts}), all larvae showed a mutant phenotype: lack of NSC reactivation and clear reduction in brain volume. In further experiments, we scored mutant phenotypes by brain volume only in third instar larvae. Twelve larvae were scored for each condition. In single temperature-shift experiments, 1 hr embryos or 1 hr first instar larvae were grown at a given temperature and shifted once. We tested 6 or 24 hr temporal windows.

dIlp6 Secretion Experiments

We coexpressed a tagged version of dIlp6 (dIlp6-FLAG) and a membrane marker (mCD8-RFP) in the BBB glia alone, in controls (genotype *moody-GAL4, UAS-mCD8-RFP, UAS-dIlp6-FLAG*) and *inx1* mutants (genotype *inx1*^{ogrejNL3}*/Y; moody-GAL4, UAS-mCD8-RFP, UAS-dIlp6-FLAG*). Larvae were starved as described in the Supplemental Experimental Procedures. All brains were imaged using the same confocal settings for dIlp6-FLAG. For each VNC, the total dIlp6 intensity (T) was calculated using Volocity software, using a fixed intensity threshold. Retained dIlp6 intensity (R) was then measured as the dIlp6 signal (same threshold as previously) colocalizing with the BBB membrane (RFP signal). The threshold for the RFP signal was determined manually for each brain, to ensure selection of the whole membrane. Due to the flatness of the BBB glia, the membrane signal represents most of the cells, although some cytoplasmic signal could be missed. The percentage of secreted dIlp6 was calculated as percent secreted dIlp6 = $(1 - R/T) \times 100$.

Membrane Polarization Experiments

moody-GAL4, *UAS-kir2.1-GFP; tubulin-GAL80*^{ts} flies were grown at permissive temperature until hatching, then switched to 30°C from ALH0 to ALH24.

Calcium Imaging

Two different conditions were analyzed: control (genotype *FM7*, *Dfd GMR YFP/Y; moody-GAL4*, *UAS-mCD8-RFP*, *UAS-GCaMP3*) and *inx1* mutant (genotype *inx1*^{ogrejNL3}*/Y; moody-GAL4*, *UAS-mCD8-RFP*, *UAS-GCaMP3*). Larvae were raised and staged at 29°C, then fed or starved as described in the Supplemental Experimental Procedures. Larvae were placed in a drop of Voltalef oil on a 22 mm diameter Welco dish, ventral side down. A 13 mm diameter round coverslip was placed tilted on top and lowered until the larva was immobilized.

Mounted larvae were imaged with a 40× oil immersion objective on an Olympus inverted FV1000. One focal plane encompassing as much as possible of the GCaMP signal was imaged. GCaMP and mCD8-RFP were imaged simultaneously because we did not detect any bleed-through.

Figure 5. Coordinated Calcium Oscillations in the BBB Glia Depend on Gap Junctions

(A) GCaMP3 and a membrane marker were driven specifically in the BBB glia (*moody-Gal4*, *UAS-mCD8-RFP*, *UAS-GCaMP3*).

(B–B′′) Calcium oscillation in BBB glia at ALH7 in one control brain. (B) GCaMP3 normalized mean intensity over time for the entire plane. (B′) Regions of interest (ROI, A–D) at time 0. (B′′) GCaMP3 mean intensity over time for four different ROIs across the VNC.

(C) Graph of the correlation coefficient for a fed larva before NSC enlargement. Twenty ROIs were chosen at random within the BBB glia and the plots of their normalized calcium intensity were correlated against each other. A correlation coefficient of 1 indicates perfect correlation.

(D–D′′) Calcium oscillation in BBB glia of *inx1* mutant (*inx1*^{ogrejNL3}) in one brain. (D) GCaMP3 normalized mean intensity over time for the entire plane. (D′) ROIs (A–D) at time 0. (D′′) GCaMP3 mean intensity over time for four different ROIS.

(E) Graph of the correlation coefficient for an *inx1*^{ogrejNL3} mutant. Twenty ROIs were chosen at random within the BBB glia. See Experimental Procedures for details of the imaging and data processing. Anterior to the left.

See also Figure S5, and Movies S1 and S2.

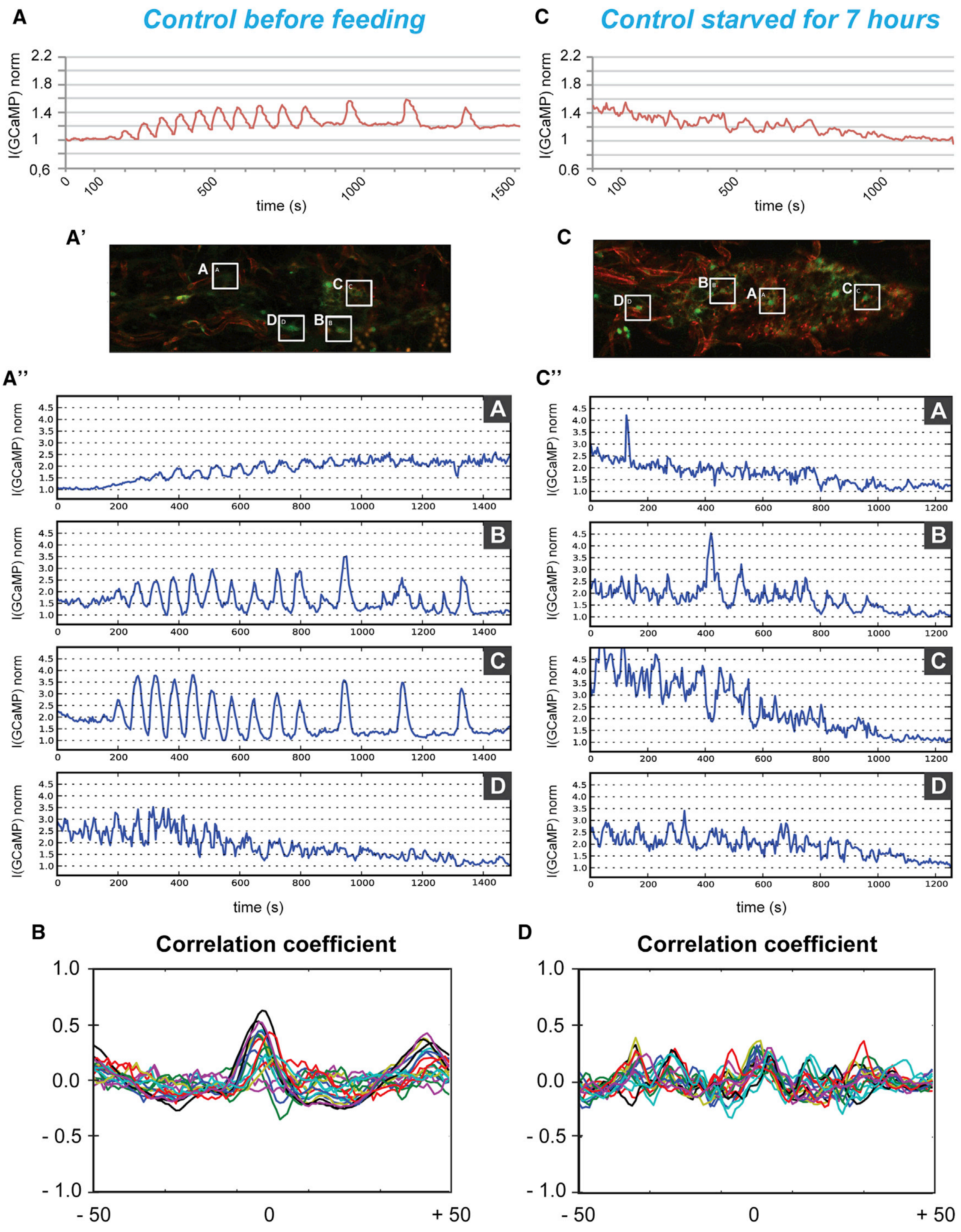

Figure 6. Coordinated Calcium Oscillations in the BBB Glia Depend on Nutritional State

(A–A′′) Calcium oscillation in BBB glia of a newly hatched control larva (ALH0). (A) GCaMP3 normalized mean intensity over time for the entire plane. (A′) ROIs (A–D) at time 0. (A′′) GCaMP3 mean intensity over time for four different ROIs.

(B) Graph of the correlation coefficient for a newly hatched control larva (ALH0). Twenty ROIs were chosen at random within the BBB glia.

(C–C′′) Calcium oscillation in BBB glia in the brain of a starved larva. (C) GCaMP3 normalized mean intensity over time for the entire plane. (C′) ROIs (A–D) at time 0. (C′′) GCaMP3 mean intensity over time for four different ROIs.

(D) Graph of the correlation coefficient for a starved larva. Twenty ROIs were chosen at random within the BBB glia. See Experimental Procedures for details of the imaging and data processing. Anterior to the left.

See also Figure S6, and Movies S3 and S4.

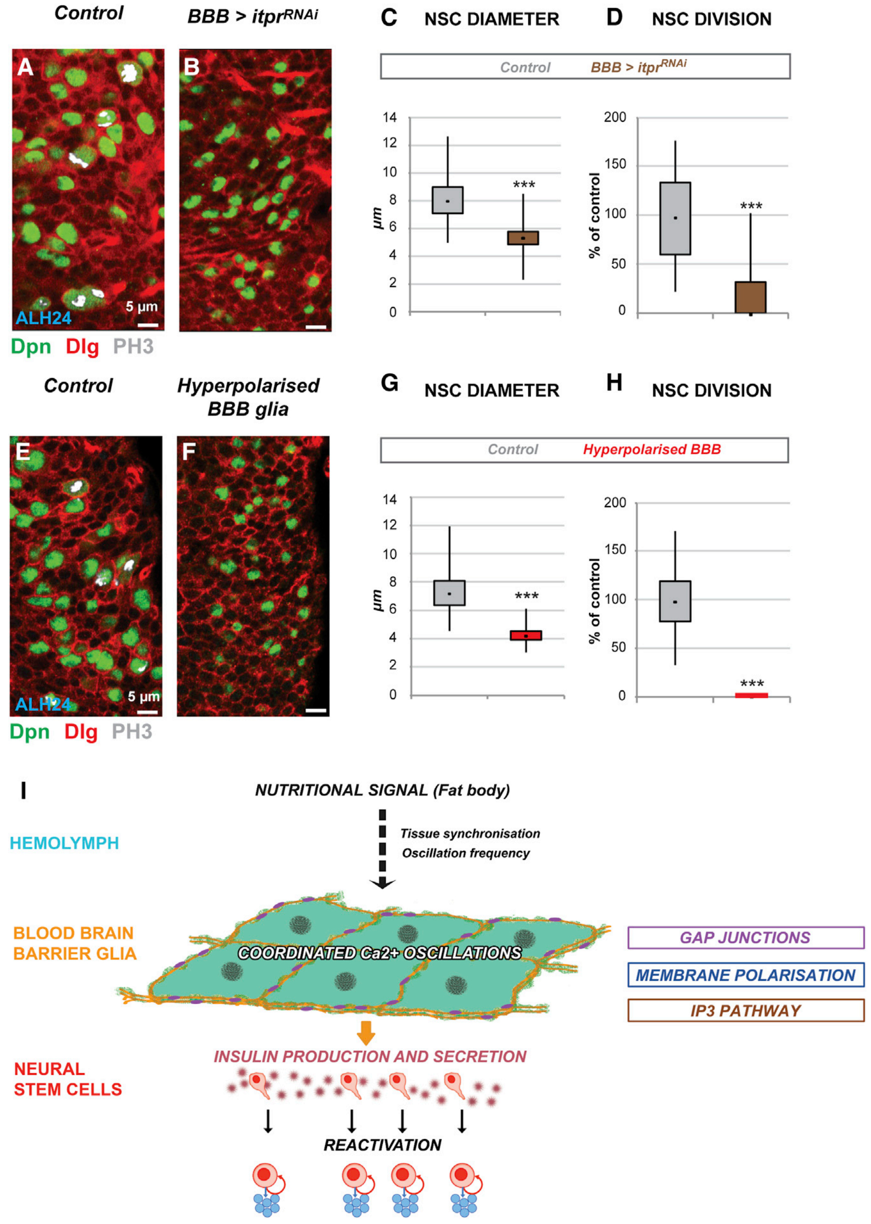

Figure 7. Calcium Signaling in the BBB Glia Is Required for NSC Reactivation

(A–D) NSC reactivation depends on the insitol-triphosphate pathway. Knocking down the insitol-triphosphate receptor in the BBB glia only (*mdr65-GAL4 > itpr^{RNAi}*) impairs NSC enlargement and proliferation (compare B to A). (C and D) Quantification of NSC (C) diameter and (D) proliferation; whisker plots. (C) ***p < 0.05. Two-sided Student's test. Average and standard deviation were calculated from two biological replicates. Control n = 352 NSCs (22 VNC); *BBB glia > itpr^{RNAi}* n = 358 NSCs (22 VNC); p = 2.31 × 10^{-116}. (D) ***p < 0.05. Two-sided Student's t test. Average and SD were calculated from two biological replicates. Control n = 22 VNCs; *BBB glia > itpr^{RNAi}* n = 22 VNCs. p = 1.03 × 10^{-7} (unequal variance test).

(E–H) NSC enlargement and proliferation are blocked upon BBB membrane hyperpolarization (*BBB glia > UAS-kir2.1*.) (compare F to E). (G and H) Quantification of NSC (G) diameter and (H) proliferation; whisker plots. (G) ***p < 0.05. Two-sided Student's t test. Average and SD were calculated from two biological replicates. Control n = 466 NSCs (22 VNC); BBB hyperpolarization n = 366 NSCs (16 VNC). p = 3.30 × 10^{-110}. (H) ***p < 0.05. Two-sided Student's test. Average and SD were calculated from three biological replicates. Control n = 33 VNCs; BBB hyperpolarization n = 26 VNCs. p = 7.88 × 10^{-23} (unequal variance test). All images are anterior up, ventral view. NSC nuclei, green (Deadpan, Dpn); Cell cortices, red (Discs Large, Dlg); PH3, gray (phosphohistone H3).

(I) Model of the role of the BBB glia in nutrition-dependent NSC reactivation. Nutrition induces calcium oscillations, which encode the nutritional message required for NSC reactivation, in the BBB. Inx1/Inx2 heteromeric channels are necessary to synchronize calcium oscillations throughout the BBB and regulate insulin production and secretion.

See also Figure S7.

Calcium Data Processing

To measure the mean intensity of the whole plane over time, movies were analyzed in Volocity. The mean intensity was measured for each point both for GCAMP3 (channel 1, C1) and mCD8-RFP signals (channel 2, C2). We normalized changes in GCaMP3 intensity against changes in mCD8-RFP intensities (accounting mainly for loss of focal plane due to larval movement), and then normalized the ratio by its minimum over the whole movie, so that the baseline for all movies was always 1. The normalized GCaMP3 signal is then: $I(GCaMP)\ norm = (C1/C2) / (C1/C2)_{min}$. This normalized ratio was plotted over time using spreadsheet software.

Calcium Tracking

To follow the behavior of calcium in different regions of the brain to check for oscillation coordination, we tracked selected ROIs independently.

For this specific purpose, a tailored python script was developed (F.N. Murphy). ROIs were selected manually in the first frame of the movie, and their central coordinates recorded. The script then tracked the ROIs as they moved during the movie. A 64 × 64 pixel tracking window was centered on each ROI, and a 32 × 32 pixel subwindow in the middle of each tracking window was used for measuring intensity values. The tracking operations were performed on the relatively stable red channel (cell membrane) rather than the more dynamic green channel (the calcium signal to be measured). The script moves the tracking window by calculating the correlation between the window region from one frame to the next. The location of the peak of the correlation function indicates how far and in which direction to move the tracking window to keep it centered on the ROI. To reduce tracking drift due to rounding errors, times-two upsampling was used. To further improve tracking accuracy, the image was low pass filtered to remove noise. The upsampling and filtering were accomplished simultaneously by taking the product of the fast Fourier transforms of the tracking window and extracting the low-frequency coefficients then zero padding before taking the inverse fast Fourier transforms. The intensity measurements were performed by summing the pixel values within the 32 × 32 pixel measuring window for both the red and green channels. Normalization was then applied as described above.

Correlation Analysis

The intensity signal for each ROI (computed and normalized as described above) is processed to remove baseline wander. The baseline is calculated using a 50 sample running average filter and then subtracted out from the signal.

The remaining signal is then scaled so that the sum of the squares of the samples is one. The signal with the largest positive excursion is selected as a reference. Each remaining ROI signal is correlated with the reference for shifts of −50 to +50 samples. The result is a correlation coefficient indicating the similarity of the signals. If a signal is identical to the reference, the correlation coefficient will peak at +1 for a shift of 0. Multiple peaks will be observed if the signal is periodic. If a signal is uncorrelated with the reference, then the correlation coefficient will take small random values.

SUPPLEMENTAL INFORMATION

Supplemental Information includes Supplemental Experimental Procedures, seven figures, three tables, and four movies and can be found with this article online at http://dx.doi.org/10.1016/j.devcel.2014.05.021.

ACKNOWLEDGMENTS

We thank R. Bainton, M. Hoch, C. Klämbt, M. Landgraf, L. Looger, M. Freeman, P. Phelan, the DSHB, the VDRC, the Kyoto DGRC, and the Bloomington Drosophila Stock Center for reagents. We thank N. Lawrence for help with the OMX microscope, F.N. Murphy for writing the calcium tracking and correlation analysis scripts, J.M. Chell and E. Viré for helpful advice, and K. Gold and P. Fox for comments on the manuscript. P.S. was funded by an EMBO long-term fellowship and a Sir Henry Wellcome postdoctoral fellowship. This work was funded by Wellcome Trust Programme grants 068055 and 092545 to A.H.B. A.H.B acknowledges core funding to the Gurdon Institute from the Wellcome Trust (092096) and CRUK (C6946/A14492).

Received: February 27, 2014
Revised: April 15, 2014
Accepted: May 24, 2014
Published: July 24, 2014

REFERENCES

Alonso, M.T., and García-Sancho, J. (2011). Nuclear Ca(2+) signalling. Cell Calcium *49*, 280–289.

Andersen, D.S., Colombani, J., and Léopold, P. (2013). Coordination of organ growth: principles and outstanding questions from the world of insects. Trends Cell Biol. *23*, 336–344.

Baines, R.A., Uhler, J.P., Thompson, A., Sweeney, S.T., and Bate, M. (2001). Altered electrical properties in Drosophila neurons developing without synaptic transmission. J. Neurosci. *21*, 1523–1531.

Bainton, R.J., Tsai, L.T.-Y., Schwabe, T., DeSalvo, M., Gaul, U., and Heberlein, U. (2005). moody encodes two GPCRs that regulate cocaine behaviors and blood-brain barrier permeability in Drosophila. Cell *123*, 145–156.

Banks, W.A. (2012). Brain meets body: the blood-brain barrier as an endocrine interface. Endocrinology *153*, 4111–4119.

Bauer, R., Löer, B., Ostrowski, K., Martini, J., Weimbs, A., Lechner, H., and Hoch, M. (2005). Intercellular communication: the Drosophila innexin multiprotein family of gap junction proteins. Chem. Biol. *12*, 515–526.

Berridge, M.J. (2009). Inositol trisphosphate and calcium signalling mechanisms. Biochim. Biophys. Acta *1793*, 933–940.

Bosco, D., Haefliger, J.-A., and Meda, P. (2011). Connexins: key mediators of endocrine function. Physiol. Rev. *91*, 1393–1445.

Britton, J.S., and Edgar, B.A. (1998). Environmental control of the cell cycle in Drosophila: nutrition activates mitotic and endoreplicative cells by distinct mechanisms. Development *125*, 2149–2158.

Catterall, W.A. (2011). Voltage-gated calcium channels. Cold Spring Harb. Perspect. Biol. *3*, a003947.

Chell, J.M., and Brand, A.H. (2010). Nutrition-responsive glia control exit of neural stem cells from quiescence. Cell *143*, 1161–1173.

Chorna, T., and Hasan, G. (2012). The genetics of calcium signaling in Drosophila melanogaster. Biochim. Biophys. Acta *1820*, 1269–1282.

Colombani, J., Raisin, S., Pantalacci, S., Radimerski, T., Montagne, J., and Léopold, P. (2003). A nutrient sensor mechanism controls Drosophila growth. Cell *114*, 739–749.

Daneman, R. (2012). The blood-brain barrier in health and disease. Ann. Neurol. *72*, 648–672.

Daneman, R., and Barres, B.A. (2005). The blood-brain barrier—lessons from moody flies. Cell *123*, 9–12.

Dbouk, H.A., Mroue, R.M., El-Sabban, M.E., and Talhouk, R.S. (2009). Connexins: a myriad of functions extending beyond assembly of gap junction channels. Cell Commun. Signal. *7*, 4.

De Bock, M., Wang, N., Decrock, E., Bol, M., Gadicherla, A.K., Culot, M., Cecchelli, R., Bultynck, G., and Leybaert, L. (2013). Endothelial calcium dynamics, connexin channels and blood-brain barrier function. Prog. Neurobiol. *108*, 1–20.

DeSalvo, M.K., Mayer, N., Mayer, F., and Bainton, R.J. (2011). Physiologic and anatomic characterization of the brain surface glia barrier of Drosophila. Glia *59*, 1322–1340.

Dobbie, I.M., King, E., Parton, R.M., Carlton, P.M., Sedat, J.W., Swedlow, J.R., and Davis, I. (2011). OMX: a new platform for multimodal, multichannel widefield imaging. Cold Spring Harb Protoc *2011*, 899–909.

Dolenšek, J., Skelin, M., and Rupnik, M.S. (2011). Calcium dependencies of regulated exocytosis in different endocrine cells. Physiol. Res. *60* (*Suppl 1*), S29–S38.

Elias, L.A., and Kriegstein, A.R. (2008). Gap junctions: multifaceted regulators of embryonic cortical development. Trends Neurosci. *31*, 243–250.

Fridlyand, L.E., and Philipson, L.H. (2011). Coupling of metabolic, second messenger pathways and insulin granule dynamics in pancreatic beta-cells: a computational analysis. Prog. Biophys. Mol. Biol. *107*, 293–303.

Géminard, C., Rulifson, E.J., and Léopold, P. (2009). Remote control of insulin secretion by fat cells in Drosophila. Cell Metab. *10*, 199–207.

Giaume, C., and Theis, M. (2010). Pharmacological and genetic approaches to study connexin-mediated channels in glial cells of the central nervous system. Brain Res. Brain Res. Rev. *63*, 160–176.

Giaume, C., Koulakoff, A., Roux, L., Holcman, D., and Rouach, N. (2010). Astroglial networks: a step further in neuroglial and gliovascular interactions. Nat. Rev. Neurosci. *11*, 87–99.

Holcroft, C.E., Jackson, W.D., Lin, W.-H., Bassiri, K., Baines, R.A., and Phelan, P. (2013). Innexins Ogre and Inx2 are required in glial cells for normal postembryonic development of the Drosophila central nervous system. J. Cell Sci. *126*, 3823–3834.

Jäderstad, J., Jäderstad, L.M., Li, J., Chintawar, S., Salto, C., Pandolfo, M., Ourednik, V., Teng, Y.D., Sidman, R.L., Arenas, E., et al. (2010). Communication via gap junctions underlies early functional and beneficial interactions between grafted neural stem cells and the host. Proc. Natl. Acad. Sci. USA *107*, 5184–5189.

Kar, R., Batra, N., Riquelme, M.A., and Jiang, J.X. (2012). Biological role of connexin intercellular channels and hemichannels. Arch. Biochem. Biophys. *524*, 2–15.

Kokovay, E., Shen, Q., and Temple, S. (2008). The incredible elastic brain: how neural stem cells expand our minds. Neuron *60*, 420–429.

Lacar, B., Young, S.Z., Platel, J.-C., and Bordey, A. (2011). Gap junction-mediated calcium waves define communication networks among murine postnatal neural progenitor cells. Eur. J. Neurosci. *34*, 1895–1905.

Laude, A.J., and Simpson, A.W.M. (2009). Compartmentalized signalling: Ca2+ compartments, microdomains and the many facets of Ca2+ signalling. FEBS J. *276*, 1800–1816.

Lehmann, C., Lechner, H., Lo, B., Knieps, M., Herrmann, S., Famulok, M., Bauer, R., and Hoch, M. (2006). Heteromerization of innexin gap junction proteins regulates epithelial tissue organization in Drosophila. Mol. Biol. Cell *17*, 1676–1685.

Leybaert, L., and Sanderson, M.J. (2012). Intercellular Ca(2+) waves: mechanisms and function. Physiol. Rev. *92*, 1359–1392.

Lo Turco, J.J., and Kriegstein, A.R. (1991). Clusters of coupled neuroblasts in embryonic neocortex. Science *252*, 563–566.

MacDonald, P.E., and Rorsman, P. (2006). Oscillations, intercellular coupling, and insulin secretion in pancreatic beta cells. PLoS Biol. *4*, e49.

Malmersjö, S., Rebellato, P., Smedler, E., Planert, H., Kanatani, S., Liste, I., Nanou, E., Sunner, H., Abdelhady, S., Zhang, S., et al. (2013). Neural progenitors organize in small-world networks to promote cell proliferation. Proc. Natl. Acad. Sci. USA *110*, E1524–E1532.

Mayer, F., Mayer, N., Chinn, L., Pinsonneault, R.L., Kroetz, D., and Bainton, R.J. (2009). Evolutionary conservation of vertebrate blood-brain barrier chemoprotective mechanisms in Drosophila. J. Neurosci. *29*, 3538–3550.

Naito, Y., Yamada, T., Matsumiya, T., Ui-Tei, K., Saigo, K., and Morishita, S. (2005). dsCheck: highly sensitive off-target search software for double-stranded RNA-mediated RNA interference. Nucleic Acids Res. *33*, W589–W591.

Nakagawa, S., Maeda, S., and Tsukihara, T. (2010). Structural and functional studies of gap junction channels. Curr. Opin. Struct. Biol. *20*, 423–430.

Nakase, T., and Naus, C.C.G. (2004). Gap junctions and neurological disorders of the central nervous system. Biochim. Biophys. Acta *1662*, 149–158.

Orellana, J.A., Sánchez, H.A., Schalper, K.A., Figueroa, V., and Sáez, J.C. (2012). Regulation of intercellular calcium signaling through calcium interactions with connexin-based channels. Adv. Exp. Med. Biol. *740*, 777–794.

Phelan, P. (2005). Innexins: members of an evolutionarily conserved family of gap-junction proteins. Biochim. Biophys. Acta *1711*, 225–245.

Pinsonneault, R.L., Mayer, N., Mayer, F., Tegegn, N., and Roland, J. (2011). The Blood-Brain and Other Neural Barriers. *686*, 357–369.

Rafalski, V.A., and Brunet, A. (2011). Energy metabolism in adult neural stem cell fate. Prog. Neurobiol. *93*, 182–203.

Schwabe, T., Bainton, R.J., Fetter, R.D., Heberlein, U., and Gaul, U. (2005). GPCR signaling is required for blood-brain barrier formation in Drosophila. Cell *123*, 133–144.

Segretain, D., and Falk, M.M. (2004). Regulation of connexin biosynthesis, assembly, gap junction formation, and removal. Biochim. Biophys. Acta *1662*, 3–21.

Skupin, A., and Thurley, K. (2012). Calcium signaling: from single channels to pathways. Adv. Exp. Med. Biol. *740*, 531–551.

Sousa-Nunes, R., Yee, L.L., and Gould, A.P. (2011). Fat cells reactivate quiescent neuroblasts via TOR and glial insulin relays in Drosophila. Nature *471*, 508–512.

Stojilkovic, S.S. (2012). Molecular mechanisms of pituitary endocrine cell calcium handling. Cell Calcium *51*, 212–221.

Stork, T., Engelen, D., Krudewig, A., Silies, M., Bainton, R.J., and Klämbt, C. (2008). Organization and function of the blood-brain barrier in Drosophila. J. Neurosci. *28*, 587–597.

Südhof, T.C. (2012). Calcium control of neurotransmitter release. Cold Spring Harb. Perspect. Biol. *4*, a011353.

Tian, L., Hires, S.A., Mao, T., Huber, D., Chiappe, M.E., Chalasani, S.H., Petreanu, L., Akerboom, J., McKinney, S.A., Schreiter, E.R., et al. (2009). Imaging neural activity in worms, flies and mice with improved GCaMP calcium indicators. Nat. Methods *6*, 875–881.

Truman, J.W., and Bate, M. (1988). Spatial and temporal patterns of neurogenesis in the central nervous system of Drosophila melanogaster. Dev. Biol. *125*, 145–157.

Unhavaithaya, Y., and Orr-Weaver, T.L. (2012). Polyploidization of glia in neural development links tissue growth to blood-brain barrier integrity. Genes Dev. *26*, 31–36.

Willenborg, M., Belz, M., Schumacher, K., Paufler, A., Hatlapatka, K., and Rustenbeck, I. (2012). Ca(2+)-dependent desensitization of insulin secretion by strong potassium depolarization. Am. J. Physiol. Endocrinol. Metab. *303*, E223–E233.

Wong, R.C.B., Pera, M.F., and Pébay, A. (2008). Role of gap junctions in embryonic and somatic stem cells. Stem Cell Rev. *4*, 283–292.

Developmental Cell, Volume *30*

Supplemental Information

Gap Junction Proteins in the Blood-Brain Barrier Control Nutrient-Dependent Reactivation of *Drosophila* Neural Stem Cells

Pauline Spéder and Andrea H. Brand

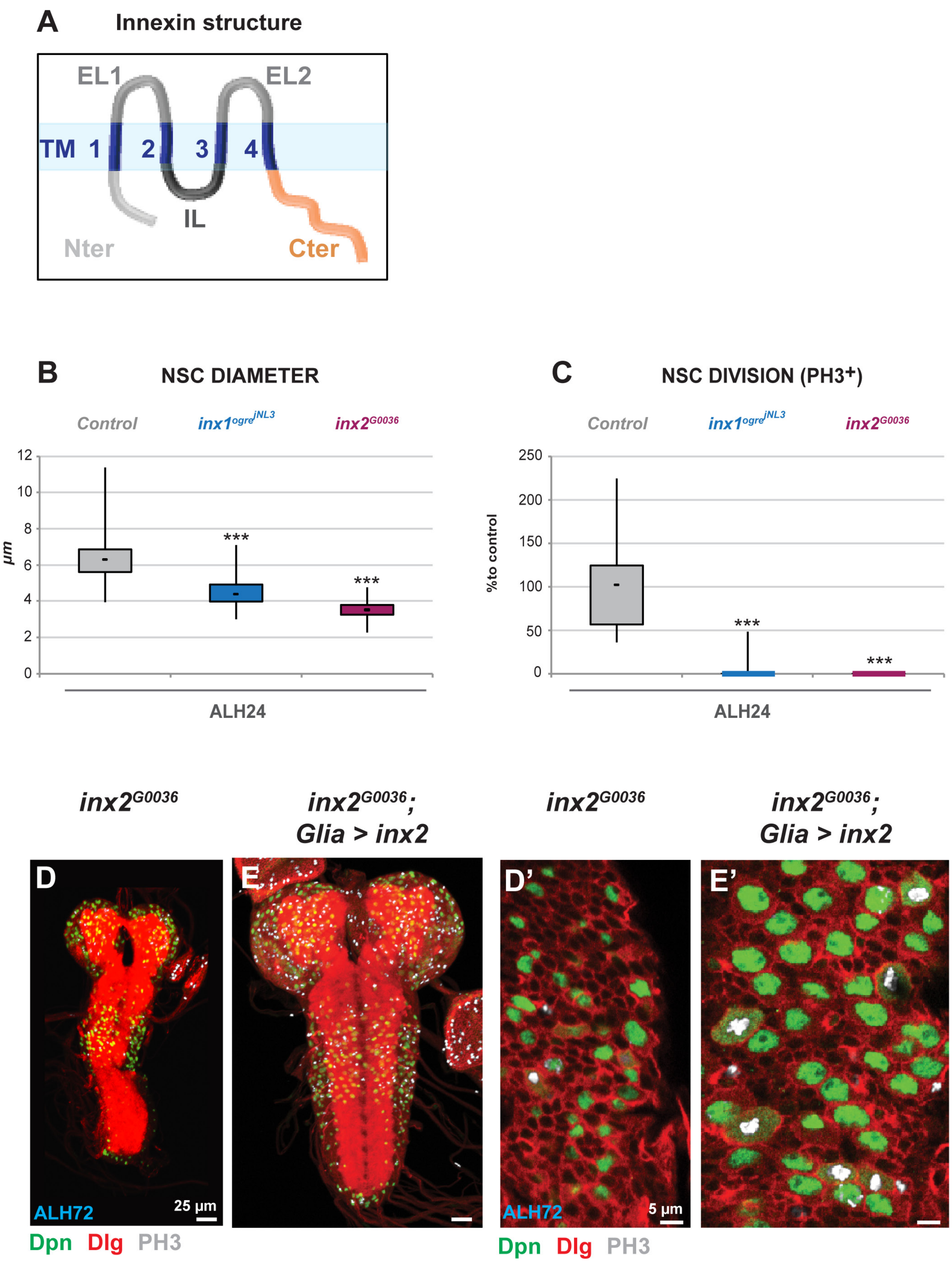
A
Innexin structure
EL1
EL2
TM 1
2
3
4
IL
Nter
Cter
B
NSC DIAMETER
Control
inx1ogre jNL3
inx2G0036
μm
***
***
ALH24
C
NSC DIVISION (PH3+)
Control
inx1ogre jNL3
inx2G0036
%to control
***
***
ALH24
inx2G0036
inx2G0036; Glia > inx2
inx2G0036
inx2G0036; Glia > inx2
D
E
D'
E'
ALH72
25 μm
ALH72
5 μm
Dpn Dlg PH3
Dpn Dlg PH3

Figure S1, related to Figure 1. Innexins are gap junction proteins required for NSC reactivation. (A) Schematic of Innexins. Inxs are comprised of a cytoplasmic N-terminal (Nter) tail, a C-terminal (Cter) tail, four transmembrane domains (TM1 to 4), two extracellular loops (EL1 and EL2) and one intracellulare loop (IL). **(B-C)** Quantification of NSC (B) diameter and (C) proliferation in *inx1* and *inx2* mutants. (B) ••• $p < 0.05$. Two-sided Student's test. Average and standard deviation were calculated from two biological replicates. Control n = 306 NSCs (16 VNC); $inx1^{ogrejNL3}$ n= 305 NSCs (16 VNCs); $inx2^{G0036}$ n = 214 NSCs (10 VNCs). $inx1^{ogrejNL3}$ $p = 1.23 \times 10^{-28}$; $inx2^{G0036}$ $p = 2.47 \times 10^{-78}$. (C) ••• $p < 0.05$. Two-sided Student's test. Average and standard deviation were calculated from two biological replicates. Control n = 16 VNCs; $inx1^{ogrejNL3}$ n =16 VNCs *;* $inx2^{G0036}$ n = 10 VNCs*.* $inx1^{ogrejNL3}$ $p = 7.78 \times 10^{-7}$. $inx2^{G0036}$ $p = 7.65 \times 10^{-7}$. (**D-E'**) Rescue of NSC reactivation in $inx2^{G0036}$ mutants by glial expression of *inx2*. (B-C) Anterior up, dorsal view. (B'-C') Higher magnification. Ventral views. NSC nuclei, green (Deadpan); Cell cortices, red (Discs Large); Phospho-histone H3, grey.

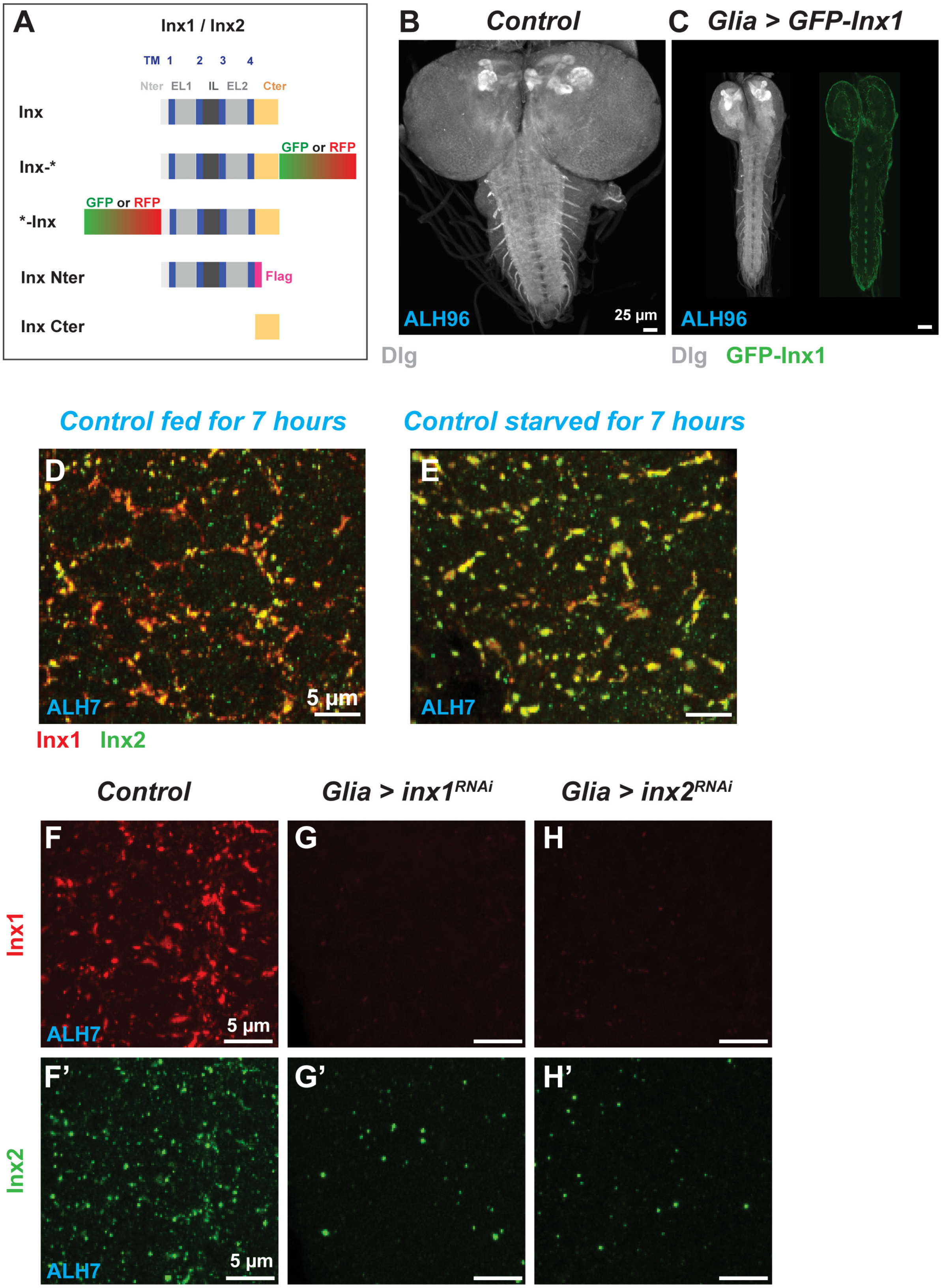
A
Inx1 / Inx2
TM 1 2 3 4
Nter EL1 IL EL2 Cter
Inx
Inx-*
GFP or RFP
*-Inx
GFP or RFP
Inx Nter
Flag
Inx Cter
B
Control
ALH96
25 µm
Dlg
C
Glia > GFP-Inx1
ALH96
Dlg GFP-Inx1
Control fed for 7 hours
Control starved for 7 hours
D
ALH7
5 µm
E
ALH7
Inx1 Inx2
Control
Glia > inx1RNAi
Glia > inx2RNAi
F
G
H
Inx1
ALH7
5 µm
F'
G'
H'
Inx2
ALH7
5 µm

Figure S2, related to Figure 2. Inx1 and Inx2 form heteromeric channels. (**A)** Five different constructs have been generated for Inx1 and Inx2. * represents GFP for Inx1 and RFP for Inx2. (**B-C**) Nter fusion acts as a dominant negative. Membrane, grey (Dlg); GFP-Inx1 , green. (**D-E**) Confocal images showing that Inx1 and Inx2 co-localisation is not lost under starvation. Inx1, red; Inx2 , green. Extended projection. (**F-H'**) Localisation of Inx1 (red) and Inx 2 (green) were assayed in glia when the reciprocal *inx* was knocked down by RNAi. Compare G and H with F and between them, and compare G' and H' with F' and between them. Notice the similarity between G and H, as well as between G' and H'.

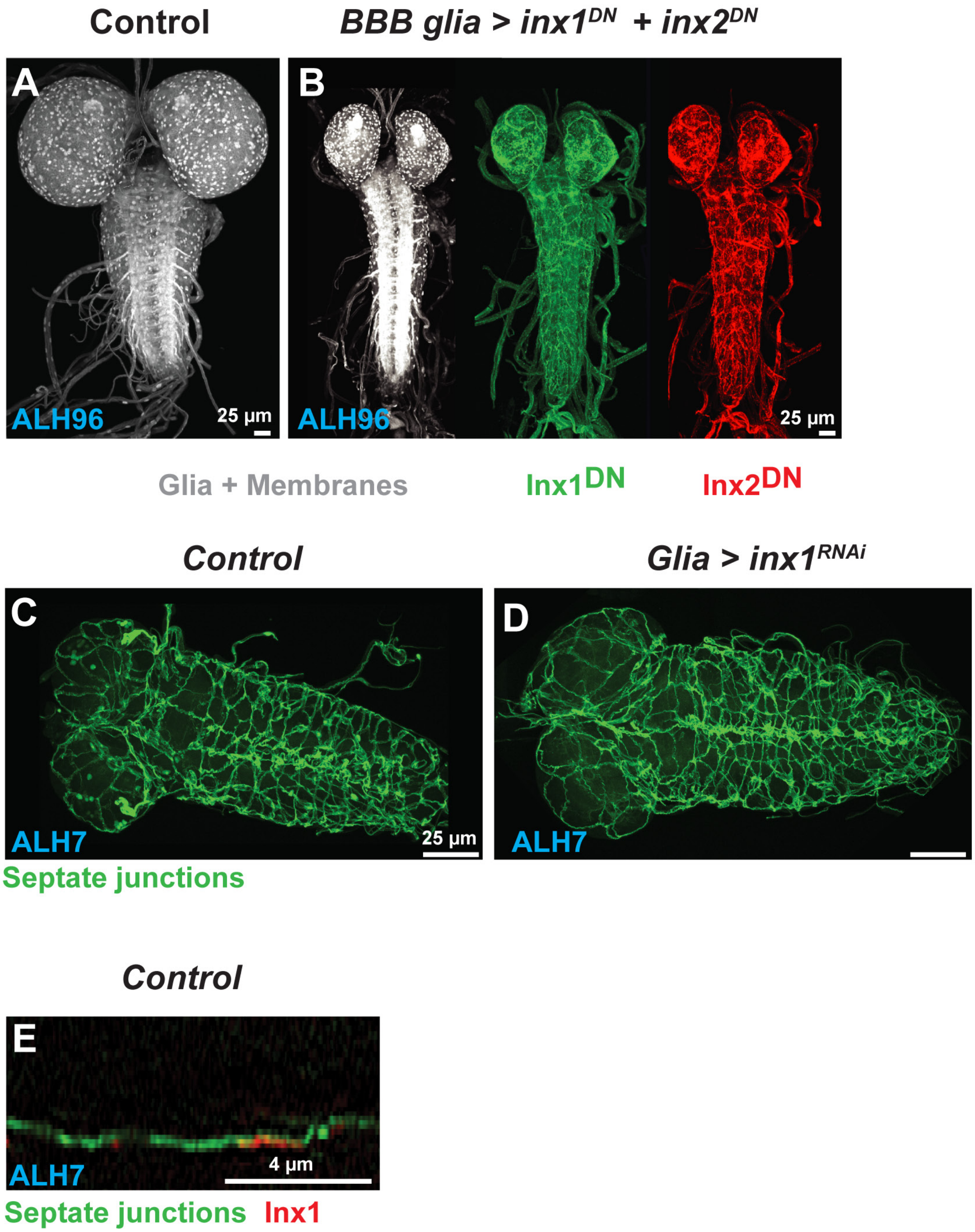

Control
BBB glia > inx1DN + inx2DN
A
B
ALH96
25 µm
ALH96
25 µm
Glia + Membranes
Inx1DN
Inx2DN
Control
Glia > inx1RNAi
C
D
ALH7
25 µm
ALH7
Septate junctions
Control
E
ALH7
4 µm
Septate junctions Inx1

Figure S3, related to Figure 3. Inx1/Inx2 channels are required in the BBB glia for NSC reactivation. (A-B) Blocking *inx1* and *inx2* function in blood-brain barrier (BBB) glia phenocopies pan-glial knockdown. Glial nuclei, grey (Repo) ; Cell cortices, grey (Dlg); $Inx1^{DN}$ (GFP-Inx1), green ; $Inx2^{DN}$ (RFP-Inx2), red. **(C-D)** Septate junctions of the BBB glia are not affected in *inx1* knock down. Septate junction stainings (green) in the whole brain of control (**C**, *Lachesin ::GFP ; repo-GAL4*) and RNAi knockdown conditions (**D**, *Lachesin ::GFP,* $inx1^{RNAi}$*; repo-GAL4*). Maximal projection. (**E**) High-resolution (OMX) close-up of a septate junction in a control brain. Orthogonal section. Septate junction, green (*Lachesin::GFP*); Inx1, red.

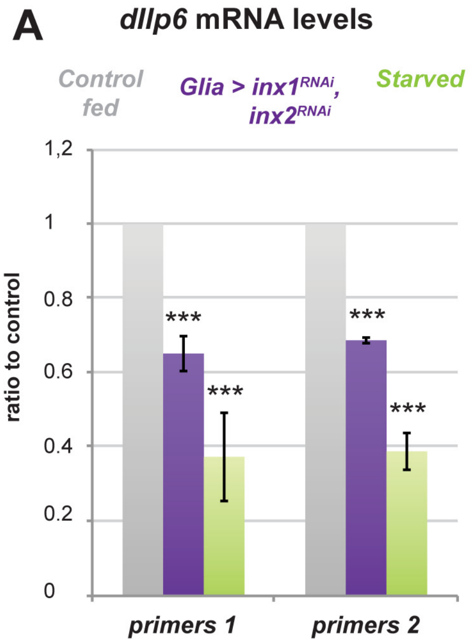
A
dIlp6 mRNA levels
Control fed
Glia > inx1RNAi, inx2RNAi
Starved
ratio to control
1,2
1
0.8
0.6
0.4
0.2
0
***
***
***
***
primers 1
primers 2

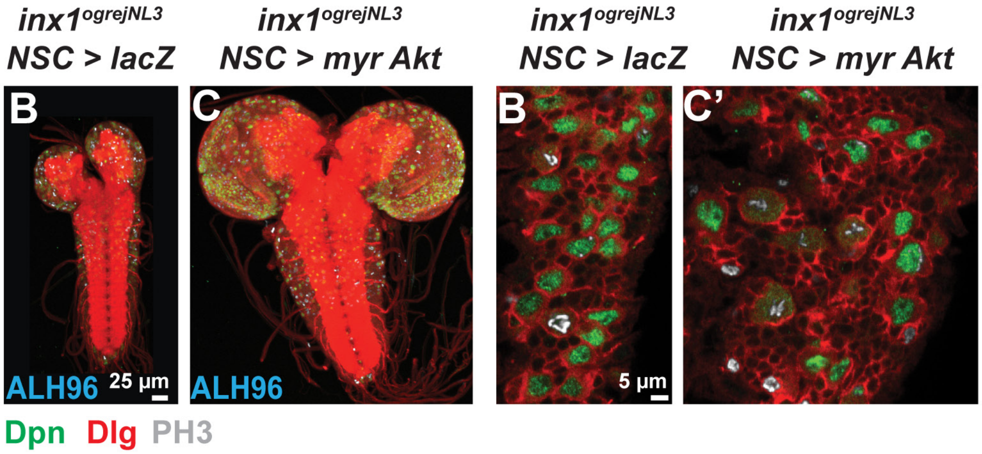
inx1ogrejNL3
NSC > lacZ
inx1ogrejNL3
NSC > myr Akt
inx1ogrejNL3
NSC > lacZ
inx1ogrejNL3
NSC > myr Akt
B
C
B
C'
ALH96
25 µm
ALH96
5 µm
Dpn
Dlg
PH3

Figure S4, related to Figure 4. Gap junctions control NSC reactivation through the insulin pathway. (**A**) Quantification of *dIlp6* transcript levels by q-pcr in glial knockdown of both *inx1* and *inx2*, compared to fed or starved controls. Two pairs of *dIlp6* primers were used. Three biological replicates. See Extended Experimental procedures for details. ••• $p < 0.05$. Two-sided Student's test. Primers 1: *Glia* > $inx1^{RNAi}$, $inx2^{RNAi}$: $p = 1.74 \times 10^{-3}$; control starved : $p = 7.96 \times 10^{-4}$. Primers 2: *Glia* > $inx1^{RNAi}$, $inx2^{RNAi}$: $p = 2.08 \times 10^{-6}$; control starved : $p = 2.83 \times 10^{-5}$. Bar graphs represent mean ± SEM. (**B-C**) Rescue of NSC reactivation in *inx1* mutants by ectopic expression of a constitutive activated form of Akt (myr-Akt) in NSCs. Anterior up, dorsal view (**B'-C'**) Higher magnification. Ventral views. NSC nuclei, green (Deadpan); Cell cortices, red (Discs Large); Phospho-histone H3, grey.

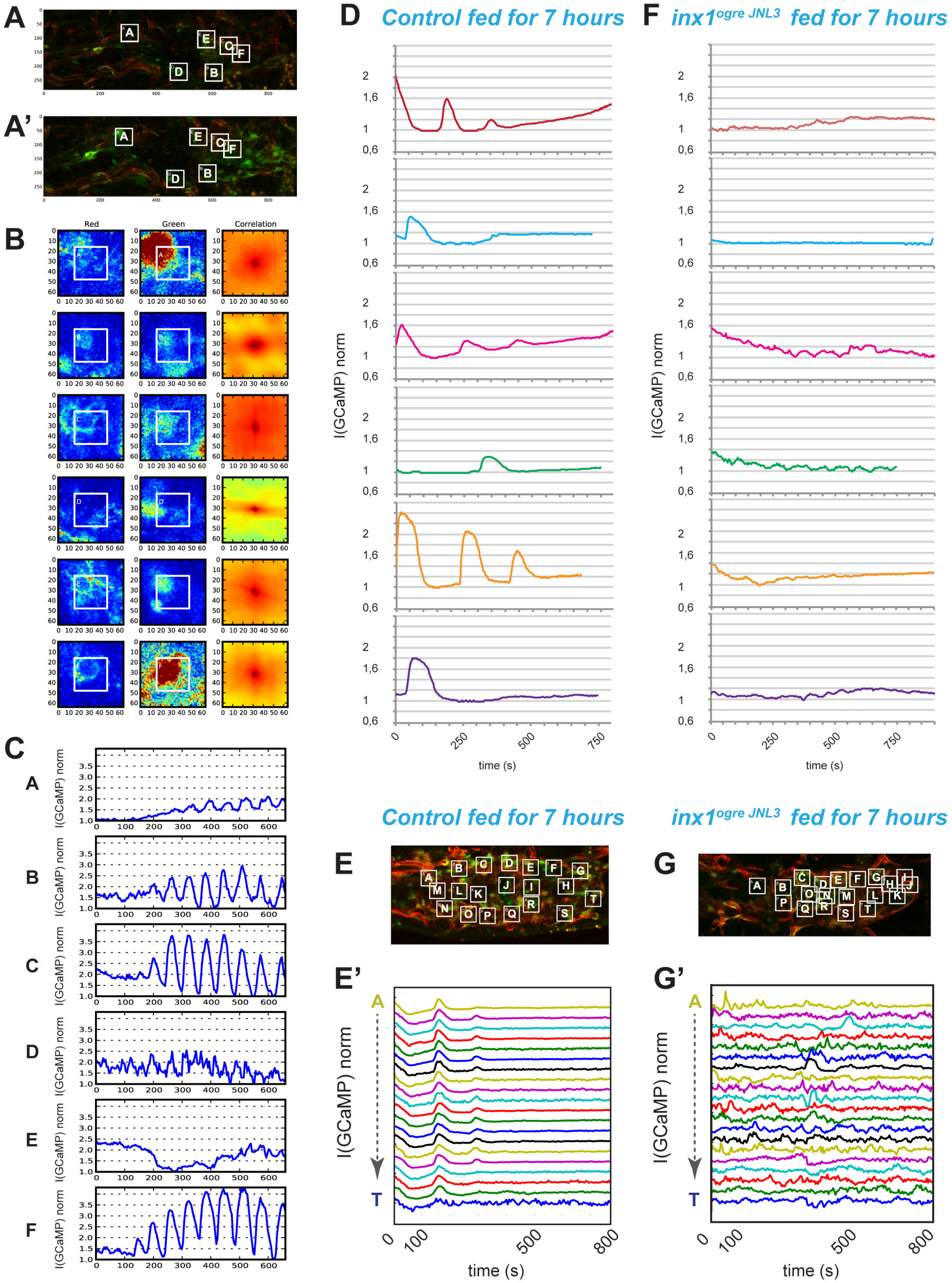

A
A'
B
Red
Green
Correlation
C
D
Control fed for 7 hours
F
inx1ogre JNL3 fed for 7 hours
I(GCaMP) norm
time (s)
E
E'
G
G'

Figure S5, related to Figure 5. Analysis of calcium oscillations in control and *inx1* mutant conditions. (A-C) Calcium tracking script. (A-A'), Snapshots of the ROI selector at time 0 (A) and 160 frames after (A'). Each selected ROI is represented by a square. (B) Snapshot of the correlation window. (C) Snapshot of live plots of normalised GCaMP3 intensities over time. (**D**) Normalised GCaMP3 intensities plotted over time for 6 brains of control fed for 7 hours. **(E-E')** Correlation analysis for a fed larva before NSC enlargement. (E) Map of the 20 ROIs at time 0. (E') Normalised calcium intensity over time for the 20 ROIs. **(F)** Normalised GCaMP3 intensities plotted over time for 6 brains of *inx1*^{ogrejNL3} fed for 7 hours. **(G-G')** Correlation analysis for an *inx1*^{ogrejNL3} mutant. (G) Map of the 20 ROIs at time 0. (G') Normalised calcium intensity over time for the 20 ROIs.

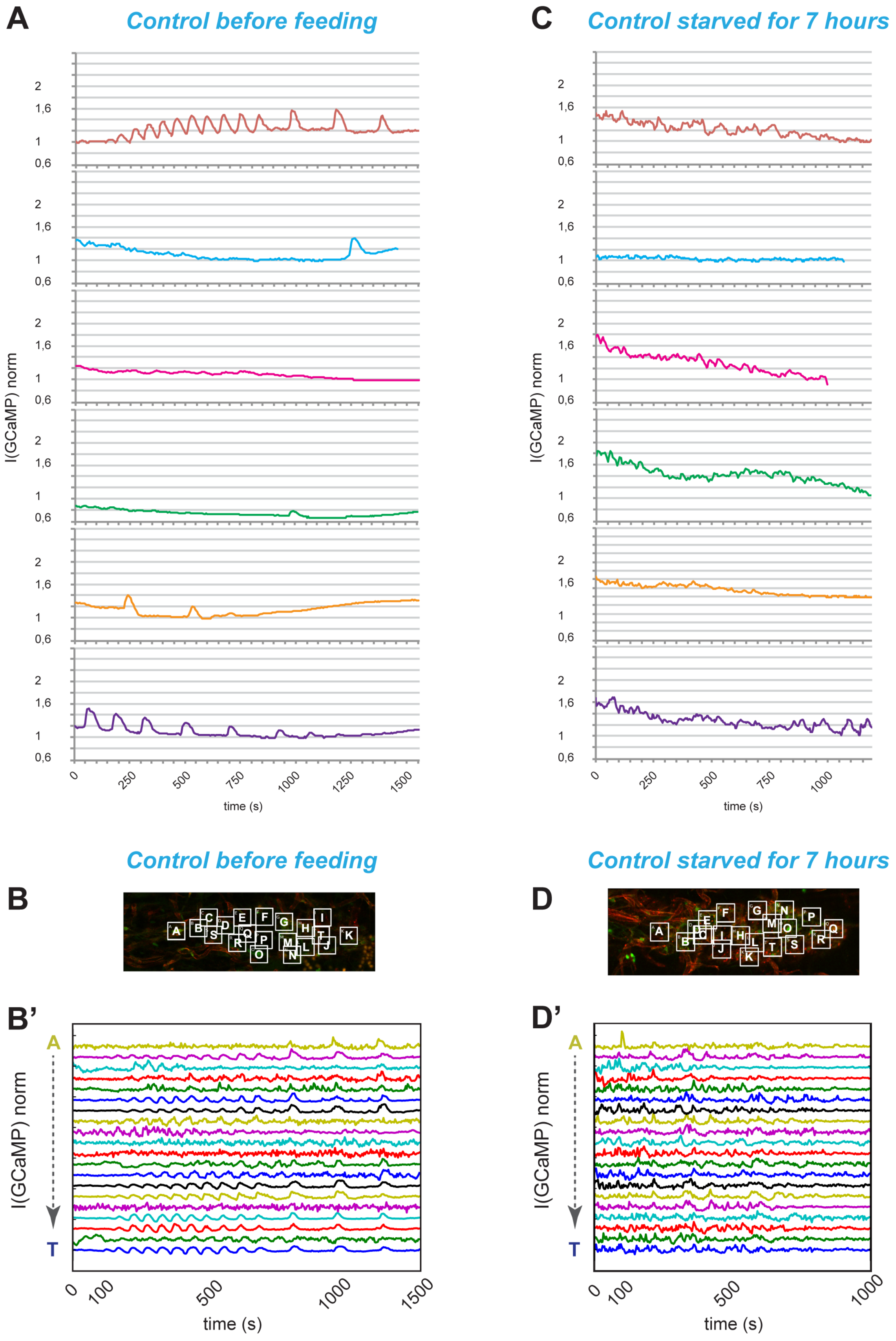
A
Control before feeding
C
Control starved for 7 hours
I(GCaMP) norm
2
1,6
1
0,6
0
250
500
750
1000
1250
1500
time (s)
Control before feeding
Control starved for 7 hours
B
D
A
B
C
D
E
F
G
H
I
J
K
L
M
N
O
P
Q
R
S
T
B'
D'
I(GCaMP) norm
A
T
0
100
500
1000
1500
time (s)

Figure S6, related to Figure 6. Analysis of calcium oscillations in control conditions before feeding and after starvation. (**A**) Normalised GCaMP3 intensities plotted over time for 6 brains of control before feeding. **(B-B')** Correlation analysis for a newly hatched control larva (ALH0). (B) Map of the 20 ROIs at time 0. (B') Normalised calcium intensity over time for the 20 ROIs. **(C)** Normalised GCaMP3 intensities plotted over time for 6 brains of control starved for 7 hours. **(D-D')** Correlation analysis for a starved larva. (D) Map of the 20 ROIs at time 0. (D') Normalised calcium intensity over time for the 20 ROIs.

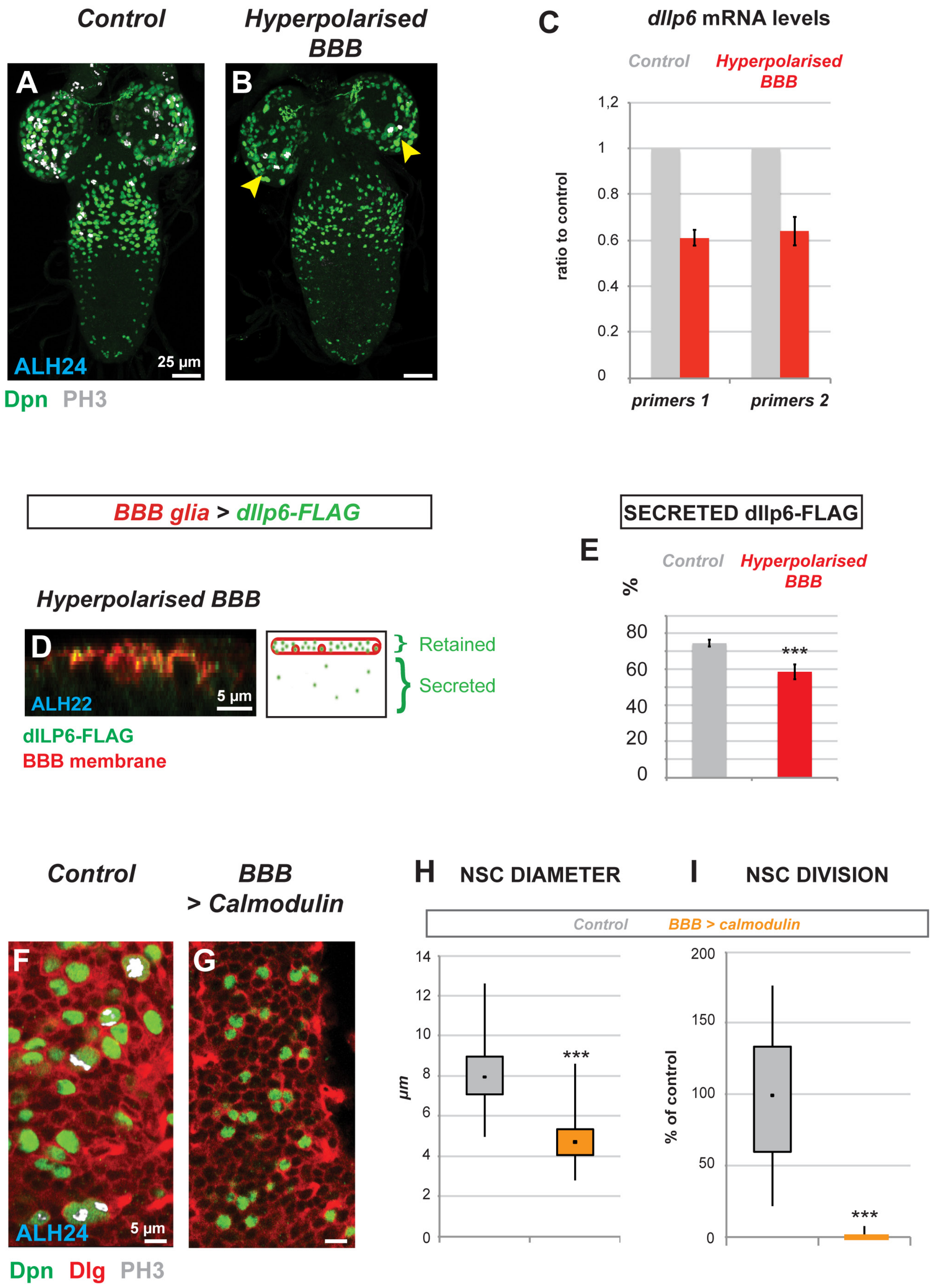
Control
Hyperpolarised BBB
A
B
ALH24
25 µm
Dpn PH3
C
dIlp6 mRNA levels
Control
Hyperpolarised BBB
ratio to control
1,2
1
0.8
0.6
0.4
0.2
0
primers 1
primers 2
BBB glia > dIlp6-FLAG
SECRETED dIlp6-FLAG
Hyperpolarised BBB
D
ALH22
5 µm
Retained
Secreted
dILP6-FLAG
BBB membrane
E
%
Control
Hyperpolarised BBB
80
60
40
20
0
***
Control
BBB > Calmodulin
F
G
ALH24
5 µm
Dpn Dlg PH3
H NSC DIAMETER
I NSC DIVISION
Control BBB > calmodulin
µm
14
12
10
8
6
4
2
0
***
% of control
200
150
100
50
0
***

Figure S7, related to Figure 7. Calcium signalling is required for NSC reactivation. (**A-B**) Nutrient-dependent NSC enlargement and division are blocked when the BBB glia is hyperpolarised, but the mushroom body neuroblasts, which do not enter quiescence, still divide (see arrowheads in B). NSC nuclei, green (Deadpan); Phospho-histone H3, grey. (**C**) Quantification of *dIlp6* transcript levels by q-pcr under BBB hyperpolarisation. Two pairs of *dIlp6* primers were used. Two biological replicates. See Extended Experimental procedures for details. ••• $p < 0.05$. Two-sided Student's test. Primers 1: $p=3.42 \times 10^{-4}$. Primers 2: $p = 4.40 \times 10^{-3}$. Bar graphs represent mean ± SEM. (**D-E**) Measure of dIlp6-FLAG secretion from the BBB glia upon BBB glia hyperpolarisation (*UAS-kir2.1-GFP; mdr65-GAL4, dIlp6-FLAG*), compared to control (*UAS-mCD8-GFP; mdr65-GAL4, dIlp6-FLAG*). (D) Cross section of one BBB glial cell and its schematic for hyperpolarisation condition. dIlp6-FLAG, green (FLAG); BBB membrane, red (RFP). (E) Measure of secreted dIlp6-FLAG (%). See Experimental procedures for details. ••• $p < 0.05$. Two-sided Student's test. Control n = 16 VNCs; hyperpolarised BBB n= 18 VNCs. $p = 1.73 \times 10^{-3}$ (unequal variance). Bar graphs represent mean ± SEM. (**F-I**) Calmodulin overexpression (*mdr65-GAL4, UAS-calmodulin*) blocks NSC reactivation (compare G to F). All images are anterior up, ventral view. NSC nuclei, green (Deadpan, Dpn); Cell cortices, red (Discs Large, Dlg); PH3, grey (phospho-histone H3). (H-I) Quantification of NSC (H) diameter and (I) proliferation; whisker plots. (H) ••• $p < 0.05$. Two-sided Student's test. Average and standard deviation were calculated from two biological replicates. Control n = 352 NSCs (22 VNC); *BBB glia* > *calmodulin* n = 328 NSCs (21 VNC); $p = 4.91 \times 10^{-163}$. (I) ••• $p < 0.05$. Two-sided Student's test. Average and standard deviation were calculated from two biological replicates. Control n = 22 VNCs; *BBB glia* > *calmodulin* n= 21 VNCs. $p = 2.19 \times 10^{-9}$ (unequal variance test).

Supplementary ables

**Gene**	**RNAi lines**	**Brain phenotype with *tub-GAL80^{ts}; insc-GAL4* (NSC knockdown)**	**Brain phenotype with *repo-GAL4* (glial knockdown)**
inx1 (ogre)	VDRC 7136 (GD library)	-	+++
	VDRC 103816 (KK library)	-	+++
	TRiP JF02595 (BL-27283)	-	++
inx2	***TRiP JF02446 (BL-29306)***	?	+++
inx3	VDRC 44767 (GD library)	-	-
	VDRC 39094(GD library)	-	-
	VDRC 39095(GD library)	-	-
	TRiP HM05245 (BL-30501)	-	-
inx4 (zpg)	VDRC 33277 (GD library)	?	-
inx5	VDRC 6950 (GD library)	-	-
	VDRC 6951 (GD library)	-	-
inx6	VDRC 8638 (GD library)	-	-
	VDRC 8639 (GD library)	-	-
	VDRC 46398 (GD library)	-	-
	VDRC 46399 (GD library)	-	-
inx7	VDRC 22948 (GD library)	-	-
	VDRC 22949 (GD library)	-	-
inx8 (ShakB)	VDRC 26801 (GD library)	-	-
	VDRC 26802 (GD library)	-	-

Table S1, related to Figure 1. *inxs* knockdown experiments. Neural defects were screened for overall brain volume and defects in an anti-Chaoptin staining. -, no obvious phenotype detected ; +, phenotype detected (the number of crosses represents the intensity of the phenotype); ?, small defects, not investigated further. The RNAi lines for *inx1* and *inx2* used in this study are shown as $inx1^{RNAi}$ and $inx2^{RNAi}$, in bold and italic.

	Shift 25 to 30°C		Shift 30 to 25°C	
ALH	% of brains with volume < 75% of control average	% of brains with volume > 75% of control average	% of brains with volume < 75% of control average	% of brains with volume > 75% of control average
-24	100	0	25	75
-18	100	0	25	75
-12	100	0	50	50
-6	100	0	75	25
0	100	0	100	0
24	100	0	100	0
48	0	100	100	0
72	0	100	100	0

No shift, 30°C from egg laying to analysis
No shift, 25°C from egg laying to analysis

Table S2, related to Figure 2. Temporal mapping of *inx1* function. For each temperature shift, brains with volume either under or over 75% of average volume of control were counted towards mutant or wild-type phenotype, respectively.

GAL4 driver	Reference	Brain expression	X *UAS-inx1*^{RNAi}, *UAS-inx2*^{RNAi}		X *UAS-inx1*^{DN}, *UAS-inx2*^{DN}	
			Brain phenotype	Other	Brain phenotype	Other
repo-GAL4	(Sepp et al., 2001)	all glia	+++	lethal 3rd instar	+++	
gcm-GAL4	(Chotard et al., 2005)	all glia	+++	lethal 3rd instar	+++	
moody-GAL4	(Schwabe et al., 2005)	subperineurial glia	++	phenotype at 29°C (elongated VNC)	+++	no phenotype per se at 25°C
moody-GAL4 ; UAS-GAL4	This study	subperineurial glia	++	phenotype at 29°C (elongated VNC)	ND	no phenotype per se at 25°C
NP2276	(Awasaki et al., 2008)	subperineurial glia	-		ND	
mdr65-GAL4	(Pfeiffer et al., 2008)	subperineurial glia	+++	phenotype at 29°C (elongated VNC)	+++	phenotype at 29°C (elongated VNC)
NP5606 (svp-GAL4)	(Awasaki et al., 2008)	subperineurial glia + neurons	++		+++	
Mz820-GAL4	(Ito et al., 1995)	channel glia	-		ND	
nrv2-GAL4	(Sun et al., 1999)	cortex glia	-		-	
NP2222-GAL4	(Awasaki et al., 2008)	cortex glia	-		-	
NP0577-GAL4	(Awasaki et al., 2008)	cortex glia	-		-	
moody-GAL4, nrv2-GAL4	This study	cortex + subperineurial glia	-		ND	
dILP6-GAL6	(Chell and Brand, 2010)	dILP6 glia	-		ND	
NP1079-GAL4	(Sousa-Nunes et al., 2011)	dILP6 glia	+		ND	
bnl-GAL4	(Kamimura et al., 2006)	glial subpopulation	-		ND	
serrate-GAL4	(Hukriede et al., 1997)	glial subpopulation	-		ND	
NP6293-GAL4	(Awasaki et al., 2008)	perineurial glia	-		ND	
c527-GAL4	(Hummel et al., 2002)	perineurial + subperineurial glia	-		ND	
NP3233-GAL4	(Awasaki et al., 2008)	astrocyte-like glia	-		ND	
NP1243-GAL4	(Awasaki et al., 2008)	astrocyte-like glia	-		ND	
alrm-GAL4	(Doherty et al., 2009)	astrocyte-like glia	-		ND	
NP6520-GAL4	(Awasaki et al., 2008)	ensheathing glia	-		ND	
Mz97-GAL4	(Hummel et al., 2002)	wrapping glia	-		ND	
c855a-GAL4	(Manseau et al., 1997)	neuroepithelium	-		ND	
NP6990-GAL4	(Awasaki et al., 2008)	neuroepithelium	-		ND	
NP0076	This study	*inx1* pattern	+		ND	
Mz1407 (inscuteable-GAL4)	(Betschinger et al., 2006)	NSC	-		ND	
grh-GAL4	(Chell and Brand, 2010)	NSC	-		ND	
prospero-GAL4	(Kambadur et al., 1998)	NSC + some GMC/neurons	-		ND	
grh-GAL4; repo-GAL4	This study	NSC + glia	+++	lethal 3rd instard	ND	
c155 (elav-GAL4)	(Lin and Goodman, 1994)	neurons	-		ND	
e22c-GAL4	(Brand and Perrimon, 1993)	epidermis	-	embryonic lethal	ND	
69B-GAL4	(Brand and Perrimon, 1993)	epidermis	-	semi-lethal	ND	
armadillo-GAL4	(Sanson et al., 1996)	ubiquitous	-	viable	ND	
tubulin-GAL4	(Lee and Luo, 1999)	ubiquitous	++	lethal 3rd instar	ND	

Table S3, related to Figure 3. List of all the GAL4 drivers used in this study, and associated phenotypes when driving different *inx* knockdown tools. ND, Not Determined. +++: 100% of the brains examined displayed a clear reduction in brain size. ++: at least 50% of the brains examined displayed a clear reduction in brain size. +: at least 25% of the brains examined displayed a clear reduction in brain size. -: no phenotype. Reductions in brain size were determined by the investigator after visual inspection under a confocal microscope.

Supplementary movie legends

Movie S1, related to Figure 5. The blood-brain barrier glia show spontaneous synchronised calcium oscillations. Live-imaging of the BBB of a young (ALH7) fed control larva. Calcium, green (GCaMP); membrane, red (mCD8-RFP). A duration of 1s in the movie corresponds to 70s during the experiment. The total length of the recording is approximately 15 min.

Movie S2, related to Figure 5. Coordinated calcium oscillations are lost when BBB gap junctions are disrupted. Live-imaging of the BBB of a young (ALH7) $inx1^{ogre\ jNL3}$ larva. Calcium, green (GCaMP); membrane, red (mCD8-RFP). A duration of 1s in the movie corresponds to 70s during the experiment. The total length of the recording is approximately 15 min.

Movie S3, related to Figure 6. Nutrition shapes and synchronises calcium oscillations in the blood-brain barrier glia. Live-imaging of the BBB of a just hatched (ALH0) control larva. Calcium, green (GCaMP); membrane, red (mCD8-RFP). A duration of 1s in the movie corresponds to 70s during the experiment. The total length of the recording is approximately 25 min.

Movie S4, related to Figure 6. Starvation prevents the synchronisation of calcium oscillations in the blood-brain barrier glia. Live-imaging of the BBB of a young (ALH7) starved control larva. Calcium, green (GCaMP); membrane, red (mCD8-RFP). A duration of 1s in the movie corresponds to 70s during the experiment. The total length of the recording is approximately 21 min.

Supplementary Experimental Procedures

Antibodies: Rabbit and guinea pig Inx1 antibodies were made using a C-terminal peptide (EFAKQVEPSKHDRAK), following the procedure described previously (Watanabe and Kankel, 1992).

DNA subcloning: UAS-Inx1 (full length amino acid (aa) 1-362), UAS-Inx1 Nter (aa 1-287), UAS-Inx1 Cter (aa 288-362), UAS-Inx1-GFP (aa 1-362) and UAS-GFP-Inx1 (aa 2-362) were cloned in the Gateway Carnegie collection, as were UAS-Inx2 (aa 1-367), UAS-Inx2 Nter (aa 1-287), UAS-Inx2 Cter (aa 288-367), UAS-Inx2-RFP (aa 1-367) and UAS-RFP-Inx2 (aa 2-367). Domains were determined according to previous studies (Bauer et al., 2005). For each construct, several independent transgenic lines were generated and tested. *UAS-dIlp6-FLAG* was generated by cloning the full length dIlp6 (aa 1-107) in frame with the pTWF vector of the Gateway Carnegie collection.

Larval culture: Larvae that hatched within a 60 min window (defined as after larval hatching 0h, or ALH0) were transferred to fresh yeast on standard fly food. Starvation was achieved by transferring larvae to a solution of 20% sucrose in PBS.

Immunohistochemistry: Larval brains were dissected according to standard procedures. Primary antibodies used were: Chicken anti-GFP (1/2000, 06-896, Upstate), rabbit anti-RFP (1/1000, ab62341, Abcam), rabbit or guinea pig anti-Inx1 (1/200, this study), rabbit anti-Inx2 (1/30, kind gift of M. Hoch and R. Bauer), mouse anti-Discs Large (1/100, 4F3 c, DSHB), Guinea Pig anti-Deadpan (1/5000, Brand lab), mouse anti-Repo (1/100, c 8D12, DSHB), rabbit anti-PH3 (1/100, 06-570, Millipore), mouse anti-FLAG (1/1000, M2, Sigma), mouse anti-Chaoptin (1/10, 24B10, DSHB). Samples were imaged on an Olympus Upright or Inverted FV1000 confocal microscope, or with a DeltaVision OMX microscope.

Image processing: Volocity or Fiji were used to process confocal data. Adobe Photoshop and Illustrator were used to assemble figures.

Quantitative Real-Time PCR: Total RNA was extracted from 60-75 VNCs per condition using TriZol reagent (Invitrogen). cDNA was prepared using the QuantiTect Reverse Transcription Kit (Qiagen). Q-pcr was performed on an Applied Biosystems StepOnePlus™ Real-Time PCR System machine), using SYBR green (Fast SYBR® Green Master Mix, Qiagen). Results were analysed using the Pfaffl method (Pfaffl, 2001), normalising against the geometric mean of three housekeeping genes: *GAPDH1*, *Act5C* and *rp49*. The primers were all used at 60°C annealing temperature, and their efficiencies were assayed on standard curves. Primers are listed below (F, forward primer; R, reverse primer):

Primers	Sequence 5' to 3'	Amplicon (bp)	Efficiency (60°C)	Design
dILP6 Pair1 F	TGGCCCTTGGCGATGTATTTCC	84	1,87467	Quant Prime
dILP6 Pair1 R	ACTTGCAGCACAAATCGGTTACG			
dILP6 Pair2 F	CGATGTATTTCCCAACAGTTTCG	63	1,85241	(Grönke et al., 2010)
dILP6 Pair2 R	AAATCGGTTACGTTCTGCAAGTC			
GAPDH F	ATTTCGCTGAACGATAAGTTCGT	76	1,87403	(Chell and Brand, 2010)
GAPDH R	CGATGACGCGGTTGGAGTA			
Act5c F	AAGTTGCTGCTCTGGTTGTCG	280	1,83709	Quant Prime
Act5c R	GCCACACGCAGCTCATTGTAG			
rp49 F	CCAAGATCGTGAAGAAGCG	143	1,85149	Quant Prime
rp49 R	GTTGGGCATCAGATACTGTC			

Each condition was assayed with three or two biological replicates, each of them subjected to two independent reverse transcription reactions (RT). Each RT was tested in triplicate.

Dextran permeability: We followed previously published procedures (Hatan et al., 2011), with the following modifications: the permeant dye was 10-kDa Dextran (Texas Red, lysine fixable, D-1863, Invitrogen) at 50 mM for final concentration, and the bathing medium was HL-6. 10 larvae were analysed for each condition.

Brain explant culture: Brain explants of ALH0 larvae were cultured in Schneider's medium (Gibco) supplemented with 10% Fetal Calf Serum (Sigma), 2 mM L-Glutamine (25030-032, Gibco) and Pen/Strept 1X (15070-063, Gibco) following published procedures (Britton and Edgar, 1998). The only modification was to use Nunclon 4-well round dishes (Sigma) and to fill the space between the wells with 1 ml H_2O_2 to enrich the atmosphere with oxygen. Freshly dissected third instar fat body was added to trigger NSC reactivation in the corresponding well. Carbenoxolone was added at a final concentration of 0.1 mM.

dIlp6 secretion experiments. For assessing dIlp6 secretion during BBB hyperpolarisation, the control condition was *tub-GAL80^{ts}, UASmCD8-GFP; mdr65-GAL4, dIlp6-FLAG* and the hyperpolarisation condition was *tub-GAL80^{ts}, UAS-kir2.1-GFP; mdr65-GAL4, dIlp6-FLAG*. We previously checked that kir2.1-GFP and mCD8-GFP were localising the same way in the BBB

membrane, by coexpressing each of them with mCD8-RFP. Retained dIlp6 intensity was measured as the dIlp6 signal colocalising with the BBB membrane (GFP signal). The measure was then conducted as described in Experimental procedures.

Calcium imaging: Fed/older larvae proved to be more sensitive to mechanical pressure and it was difficult to achieve a perfect and harmless immobilisation for a long period of time. This was the cause of the different recording lengths between control and *inx1* mutant at ALH7-10, versus control at ALH0 or after starvation. We decided to stop the movie at a maximum of 15 min for these conditions. Unfortunately, none of the chemical (CO_2, tricaïne, L-glutamate) or other mechanical immobilisation (slow melting agarose) methods tested seemed to work on young larvae.

Calcium tracking: As *moody-GAL4* also drives in the tracheal system, we were careful to exclude from our analysis calcium signals coming from this tissue.

Supplementary References

Awasaki, T., Lai, S.-L., Ito, K., and Lee, T. (2008). Organization and postembryonic development of glial cells in the adult central brain of Drosophila. J. Neurosci. *28*, 13742–13753.

Bauer, R., Löer, B., Ostrowski, K., Martini, J., Weimbs, A., Lechner, H., and Hoch, M. (2005). Intercellular communication: the Drosophila innexin multiprotein family of gap junction proteins. Chem. Biol. *12*, 515–526.

Betschinger, J., Mechtler, K., and Knoblich, J. a (2006). Asymmetric segregation of the tumor suppressor brat regulates self-renewal in Drosophila neural stem cells. Cell *124*, 1241–1253.

Brand, A.H., and Perrimon, N. (1993). Targeted gene expression as a means of altering cell fates and generating dominant phenotypes. Development *118*, 401–415.

Britton, J.S., and Edgar, B. a (1998). Environmental control of the cell cycle in Drosophila: nutrition activates mitotic and endoreplicative cells by distinct mechanisms. Development *125*, 2149–2158.

Chell, J.M., and Brand, A.H. (2010). Nutrition-responsive glia control exit of neural stem cells from quiescence. Cell *143*, 1161–1173.

Chotard, C., Leung, W., and Salecker, I. (2005). glial cells missing and gcm2 cell autonomously regulate both glial and neuronal development in the visual system of Drosophila. Neuron *48*, 237–251.

Doherty, J., Logan, M. a, Taşdemir, O.E., and Freeman, M.R. (2009). Ensheathing glia function as phagocytes in the adult Drosophila brain. J. Neurosci. *29*, 4768–4781.

Grönke, S., Clarke, D.-F., Broughton, S., Andrews, T.D., and Partridge, L. (2010). Molecular evolution and functional characterization of Drosophila insulin-like peptides. PLoS Genet. *6*, e1000857.

Hatan, M., Shinder, V., Israeli, D., Schnorrer, F., and Volk, T. (2011). The Drosophila blood brain barrier is maintained by GPCR-dependent dynamic actin structures. J. Cell Biol. *192*, 307–319.

Hukriede, N.A., Gu, Y., and Fleming, R.J. (1997). A dominant-negative form of Serrate acts as a general antagonist of Notch activation. Development *124*, 3427–3437.

Hummel, T., Attix, S., Gunning, D., and Zipursky, S.L. (2002). Temporal control of glial cell migration in the Drosophila eye requires gilgamesh, hedgehog, and eye specification genes. Neuron *33*, 193–203.

Ito, K., Urban, J., and Technau, G. (1995). Distribution, classification, and development ofDrosophila glial cells in the late embryonic and early larval ventral nerve cord. Dev. Genes Evol. *204*, 284–307.

Kambadur, R., Koizumi, K., Stivers, C., Nagle, J., Poole, S.J., and Odenwald, W.F. (1998). Regulation of POU genes by castor and hunchback establishes layered compartments in the Drosophila CNS. Genes Dev. *12*, 246–260.

Kamimura, K., Koyama, T., Habuchi, H., Ueda, R., Masu, M., Kimata, K., and Nakato, H. (2006). Specific and flexible roles of heparan sulfate modifications in Drosophila FGF signaling. J. Cell Biol. *174*, 773–778.

Lee, T., and Luo, L. (1999). Mosaic analysis with a repressible cell marker for studies of gene function in neuronal morphogenesis. Neuron *22*, 451–461.

Lin, D.M., and Goodman, C.S. (1994). Ectopic and increased expression of Fasciclin II alters motoneuron growth cone guidance. Neuron *13*, 507–523.

Manseau, L., Baradaran, A., Brower, D., Budhu, A., Elefant, F., Phan, H., Philp, A. V, Yang, M., Glover, D., Kaiser, K., et al. (1997). GAL4 enhancer traps expressed in the embryo, larval brain, imaginal discs, and ovary of Drosophila. Dev. Dyn. *209*, 310–322.

Pfaffl, M.W. (2001). A new mathematical model for relative quantification in real-time RT-PCR. Nucleic Acids Res. *29*, e45.

Pfeiffer, B.D., Jenett, A., Hammonds, A.S., Ngo, T.-T.B., Misra, S., Murphy, C., Scully, A., Carlson, J.W., Wan, K.H., Laverty, T.R., et al. (2008). Tools for neuroanatomy and neurogenetics in Drosophila. Proc. Natl. Acad. Sci. U. S. A. *105*, 9715–9720.

Sanson, B., White, P., and Vincent, J.P. (1996). Uncoupling cadherin-based adhesion from wingless signalling in Drosophila. Nature *383*, 627–630.

Schwabe, T., Bainton, R.J., Fetter, R.D., Heberlein, U., and Gaul, U. (2005). GPCR signaling is required for blood-brain barrier formation in drosophila. Cell *123*, 133–144.

Sepp, K.J., Schulte, J., and Auld, V.J. (2001). Peripheral glia direct axon guidance across the CNS/PNS transition zone. Dev. Biol. *238*, 47–63.

Sousa-Nunes, R., Yee, L.L., and Gould, A.P. (2011). Fat cells reactivate quiescent neuroblasts via TOR and glial insulin relays in Drosophila. Nature *471*, 508–512.

Sun, B., Xu, P., and Salvaterra, P.M. (1999). Dynamic visualization of nervous system in live Drosophila. Proc. Natl. Acad. Sci. U. S. A. *96*, 10438–10443.

Watanabe, T., and Kankel, D.R. (1992). The l(1)ogre gene of Drosophila melanogaster is expressed in postembryonic neuroblasts. Dev. Biol. *152*, 172–183.
